# TSC/mTORC1 mediates mTORC2/AKT1 signaling in c-MYC–induced murine hepatocarcinogenesis via centromere protein M

**DOI:** 10.1172/JCI174415

**Published:** 2024-09-26

**Authors:** Yi Zhou, Shu Zhang, Guoteng Qiu, Xue Wang, Andrew Yonemura, Hongwei Xu, Guofei Cui, Shanshan Deng, Joanne Chun, Nianyong Chen, Meng Xu, Xinhua Song, Jingwen Wang, Zijing Xu, Youping Deng, Matthias Evert, Diego F. Calvisi, Shumei Lin, Haichuan Wang, Xin Chen

**Affiliations:** 1 Department of Infectious Diseases, the First Affiliated Hospital of Xi’an Jiaotong University, Xi’an, China.; 2 Department of Bioengineering and Therapeutic Sciences and Liver Center, UCSF, San Francisco, California, USA.; 3 Department of Head and Neck Oncology, Cancer Center,; 4 Department of Radiation Oncology, Cancer Center,; 5 Division of Liver Surgery, Department of General Surgery, and; 6 Laboratory of Liver Surgery, West China Hospital, Sichuan University, Chengdu, China.; 7 Cancer Biology Program, University of Hawaii Cancer Center, Honolulu, Hawaii, USA.; 8 Laboratory of Single Cell Research and Liquid Biopsy, Cancer Center, West China Hospital, Sichuan University, Chengdu, China.; 9 Department of General Surgery, the Second Affiliated Hospital of Xi’an Jiaotong University, Xi’an, China.; 10 School of Traditional Chinese Medicine, Laboratory for Clinical Medicine, Capital Medical University, Beijing, China.; 11 Department of Quantitative Health Sciences, John A. Burns School of Medicine, Honolulu, Hawaii, USA.; 12 Institute of Pathology, University of Regensburg, Regensburg, Germany.

**Keywords:** Hepatology, Oncology, Liver cancer, Mouse models, Signal transduction

## Abstract

Activated mTORC2/AKT signaling plays a role in hepatocellular carcinoma (HCC). Research has shown that TSC/mTORC1 and FOXO1 are distinct downstream effectors of AKT signaling in liver regeneration and metabolism. However, the mechanisms by which these pathways mediate mTORC2/AKT activation in HCC are not yet fully understood. Amplification and activation of c-MYC are key molecular events in HCC. In this study, we explored the roles of tuberous sclerosis complex/mTORC1 (TSC/mTORC1) and FOXO1 as downstream effectors of mTORC2/AKT1 in c-MYC–induced hepatocarcinogenesis. Using various genetic approaches in mice, we found that manipulating the FOXO pathway had a minimal effect on c-MYC–induced HCC. In contrast, loss of mTORC2 inhibited c-MYC–induced HCC, an effect that was completely reversed by ablation of TSC2, which activated mTORC1. Additionally, we discovered that p70/RPS6 and 4EBP1/eIF4E acted downstream of mTORC1, regulating distinct molecular pathways. Notably, the 4EBP1/eIF4E cascade is crucial for cell proliferation and glycolysis in c-MYC–induced HCC. We also identified centromere protein M (CENPM) as a downstream target of the TSC2/mTORC1 pathway in c-MYC–driven hepatocarcinogenesis, and its ablation entirely inhibited c-MYC–dependent HCC formation. Our findings demonstrate that the TSC/mTORC1/CENPM pathway, rather than the FOXO cascade, is the primary signaling pathway regulating c-MYC–driven hepatocarcinogenesis. Targeting CENPM holds therapeutic potential for treating c-MYC–driven HCC.

## Abstract 2

TSC/mTORC1, but not the FOXO cascade, is the major signaling pathway regulating c-MYC-driven hepatocarcinogenesis. CENPM is a downstream target of TSC2/mTORC1 pathway in c-MYC HCC

## Graphical abstract

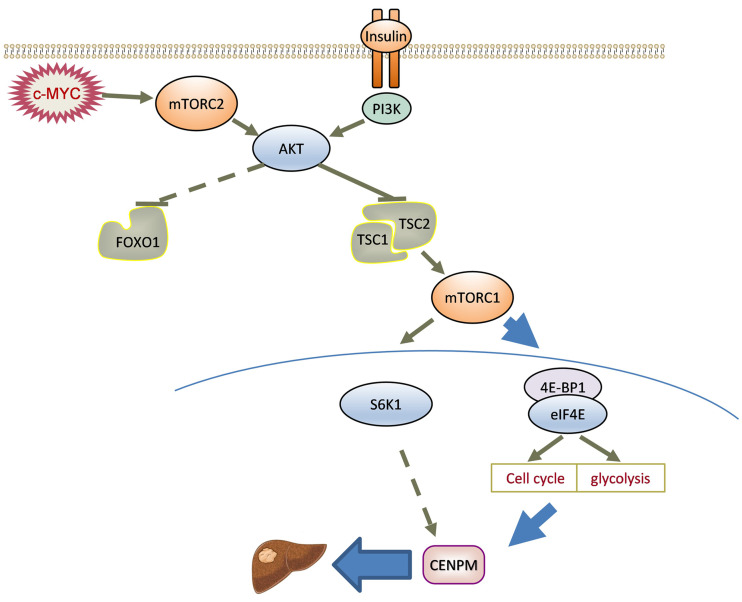

## Introduction

Hepatocellular carcinoma (HCC), the most common type of primary liver cancer, is the second leading cause of cancer death worldwide (1). Several risk factors for HCC development have been identified, including hepatitis B and hepatitis C virus infection, chronic alcohol abuse, and metabolic diseases (2). HCC is amenable to surgery and other potentially curative treatments when diagnosed early. However, effective treatment options for patients with advanced HCC are still limited. Targeted therapy with multitargeted kinase inhibitors such as sorafenib did not result in a reasonable survival benefit (3, 4), probably because of the activation of alternative pathways leading to treatment evasion. Recently, immune checkpoint inhibitor–based immunotherapy has become the first-line treatment for advanced HCC. However, a notable proportion of patients with HCC do not respond to this therapy (5, 6). Thus, there is an urgent need to investigate the molecular mechanisms leading to HCC development and progression to design novel therapies against HCC.

Activated v-akt murine thymoma viral oncogene homolog/mTOR (AKT/mTOR) signaling plays a pivotal role in human hepatocarcinogenesis (7). mTOR consists of 2 functionally distinct protein complexes: mTORC1 and mTORC2, which are distinguished by 2 unique accessory proteins, the regulatory-associated protein of mTOR (RAPTOR) and the rapamycin-insensitive companion of mTOR (RICTOR). RAPTOR and RICTOR define mTORC1 and mTORC2, respectively (8, 9). mTORC1 promotes protein synthesis by phosphorylating 2 downstream effectors, eukaryotic translation initiation factor 4E–binding protein 1 (4EBP1) and ribosomal protein S6 kinase 1 (S6K1), whereas mTORC2 phosphorylates AKT at the Ser473 site. Once activated, AKT induces mTORC1 by inhibiting its negative regulators tuberous sclerosis complex 1 (TSC1) and TSC2. In addition, AKT phosphorylates FOXO transcription factors, especially FOXO1, leading to cell growth, survival, and proliferation. Previous studies have revealed distinct TSC/mTORC1 and FOXO1 functions as downstream effectors of AKT signaling in liver regeneration and metabolism (10–12). For example, in liver regeneration, it has been shown that FOXO1 is the major downstream effector downstream of AKT (11). FOXO1 is also the key molecule regulating the AKT-mediated insulin response in the liver (13). However, how these cascades mediate mTORC2/AKT activation in HCC remains to be defined.

As a well-characterized oncogene, c-MYC activation is a critical genetic event in human HCC. Deregulated c-MYC expression triggers the selective gene expression responsible for cell growth, proliferation, metabolism, and tumorigenesis (14). Investigation of the biochemical crosstalk between c-MYC and mTOR pathways during tumor development has shown that mTORC2/AKT1 is required for c-MYC–driven HCC. Specifically, Rictor (mTORC2) or AKT1 ablation inhibits c-MYC HCC formation in mice (15, 16). In the present study, we characterized the functional role of the major downstream effectors of AKT in c-MYC–driven hepatocarcinogenesis. We discovered that TSC/mTORC1, but not the FOXO cascade, is the pivotal signaling pathway regulating c-MYC–driven HCC development. Mechanistically, we identified the centromere protein M (CENPM) as a critical downstream target of the TSC2/mTORC1 pathway in c-MYC HCC.

## Results

### Deletion of Foxo1 fails to rescue the loss of Rictor’s tumor inhibition effects in c-MYC HCC.

Our previous study demonstrated that c-MYC–driven HCC is mTORC2/AKT1 dependent. Indeed, ablation of *Rictor* or *Akt1* completely suppresses c-MYC–induced HCC formation in the mouse (15). As the first step to investigating the pathways regulated by mTORC2/AKT, we tested the hypothesis that FOXO1 is a transcription factor downstream of mTORC2/AKT1 in murine c-MYC HCC formation. Thus, we analyzed the activation status of FOXO1 in murine c-MYC HCC. While strong nuclear immunoreactivity for total and phosphorylated/inactivated FOXO1 characterized the tumor compartment and the adjacent nontumorous surrounding liver tissues, only the tumor lesions displayed robust cytoplasmic immunolabeling for the 2 proteins (Supplemental Figure 1A; supplemental material available online with this article; https://doi.org/10.1172/JCI174415DS1). In addition, Western blot analysis demonstrated increased levels of phosphorylated FOXO1 (p-FOXO1) in the mouse c-MYC tumors (Supplemental Figure 1B). Moreover, microarray analysis of mouse c-MYC HCC (17) revealed that expression of FOXO1 downstream genes was suppressed (Supplemental Figure 1C). Overall, these data indicate that FOXO1 signaling was inactivated in c-MYC HCC.

**Figure 1:**
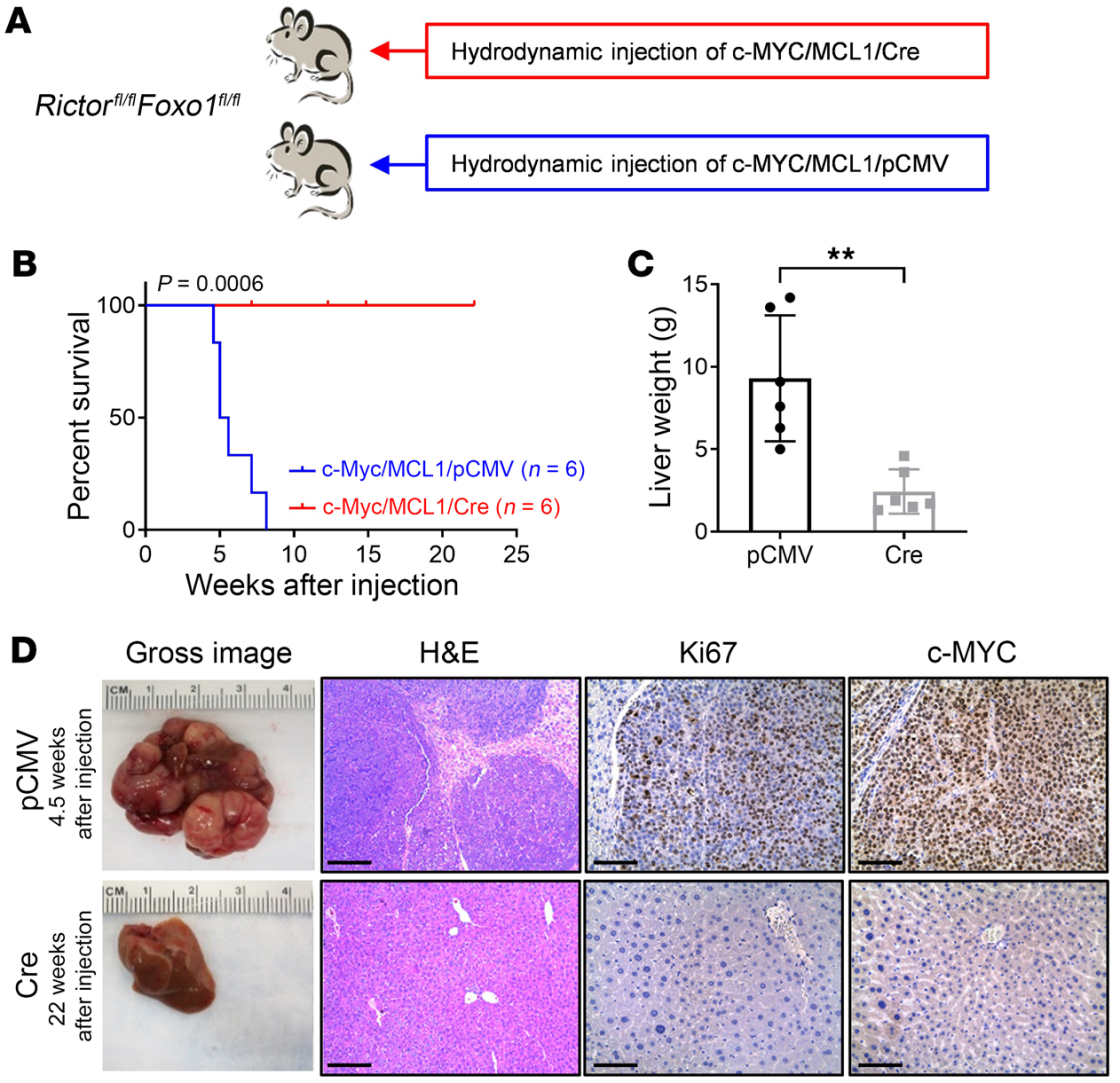
*FoxO1* deletion fails to rescue the loss of the tumor-inhibitory effects mTORC2. (**A**) Study design. *Rictor^fl/fl^* *Foxo1^fl/fl^* conditional-KO mice were hydrodynamically injected with plasmid mixtures of c-MYC/ MCL1 and Cre recombinase in a pCMV backbone (c-MYC/MCL1/Cre, *n* = 6). Control mice were hydrodynamically injected with c-MYC/MCL1 and pCMV empty vector (c-MYC/MCL1/pCMV, *n* = 6) constructs. Mice were monitored for tumor development and euthanized when moribund tumors developed or until the end of the observation period. (**B**) Survival curve for mice in both groups. A Kaplan-Meier comparison was performed; *P* = 0.0006. (**C**) Comparison of liver weights between the 2 groups. Data are presented as the mean ± SD. **P < 0.01, by 2-tailed Student’s *t* test. (**D**) Representative macroscopic images of livers, H&E stainings, and immunohistochemical staining for Ki67 and c-MYC. Scale bars: 200 μm (H&E); 100 μm (Ki67 and c-MYC).

We reasoned that if FOXO1 is the major effector, ablation of *Foxo1* would rescue the loss of *Rictor*’s tumor growth–inhibitory effects. While c-MYC could not induce HCC formation in the *Rictor*-KO genetic background, c-MYC could drive HCC development on the *Foxo1* *Rictor* double-KO background if our hypothesis is correct. Thus, we generated *Rictor^fl/fl^* *Foxo1^fl/fl^* mice on the *C57BL/6J* background. As c-MYC alone cannot induce HCC on the *C57BL/6J* genetic background, we coinjected the myeloid cell leukemia 1 (*MCL1*) oncogene into these mice, as reported before (17). In brief, *Rictor^fl/fl^* *Foxo1^fl/fl^* mice were coinjected with c-MYC, MCL1, and Cre plasmids, allowing the expression of c-MYC/MCL1 in *Rictor* *Foxo1* double-KO hepatocytes (c-MYC/MCL1/Cre). Additional *Rictor^fl/fl^* *Foxo1^fl/fl^* mice were coinjected with c-MYC, MCL1, and pCMV empty vector as controls (c-MYC/MCL1/pCMV) (Figure 1A). None of the c-MYC/MCL1/Cre–injected *Rictor^fl/fl^* *Foxo1^fl/fl^* mice developed liver tumors, even 20 weeks after injection, whereas all c-MYC/MCL1/pCMV–injected mice developed lethal tumors and required euthanasia between 4 and 8 weeks after the injection (Figure 1, B and C). The c-MYC/MCL1/Cre mouse livers appeared completely normal in gross and histological images. In contrast, poorly differentiated and highly proliferative HCC lesions were observed throughout the livers of the control group mice (Figure 1D). The phenotype recapitulated what we observed when c-MYC/MCL1/Cre plasmids were injected into *Rictor^fl/fl^* mice. The results indicate that deleting *Foxo1* failed to rescue the loss of the tumor-inhibitory effects of mTORC2.

### Overexpression of a constitutively activated FOXO1 or FOXO3 does not affect c-MYC–driven liver tumorigenesis in vivo.

**Figure 2:**
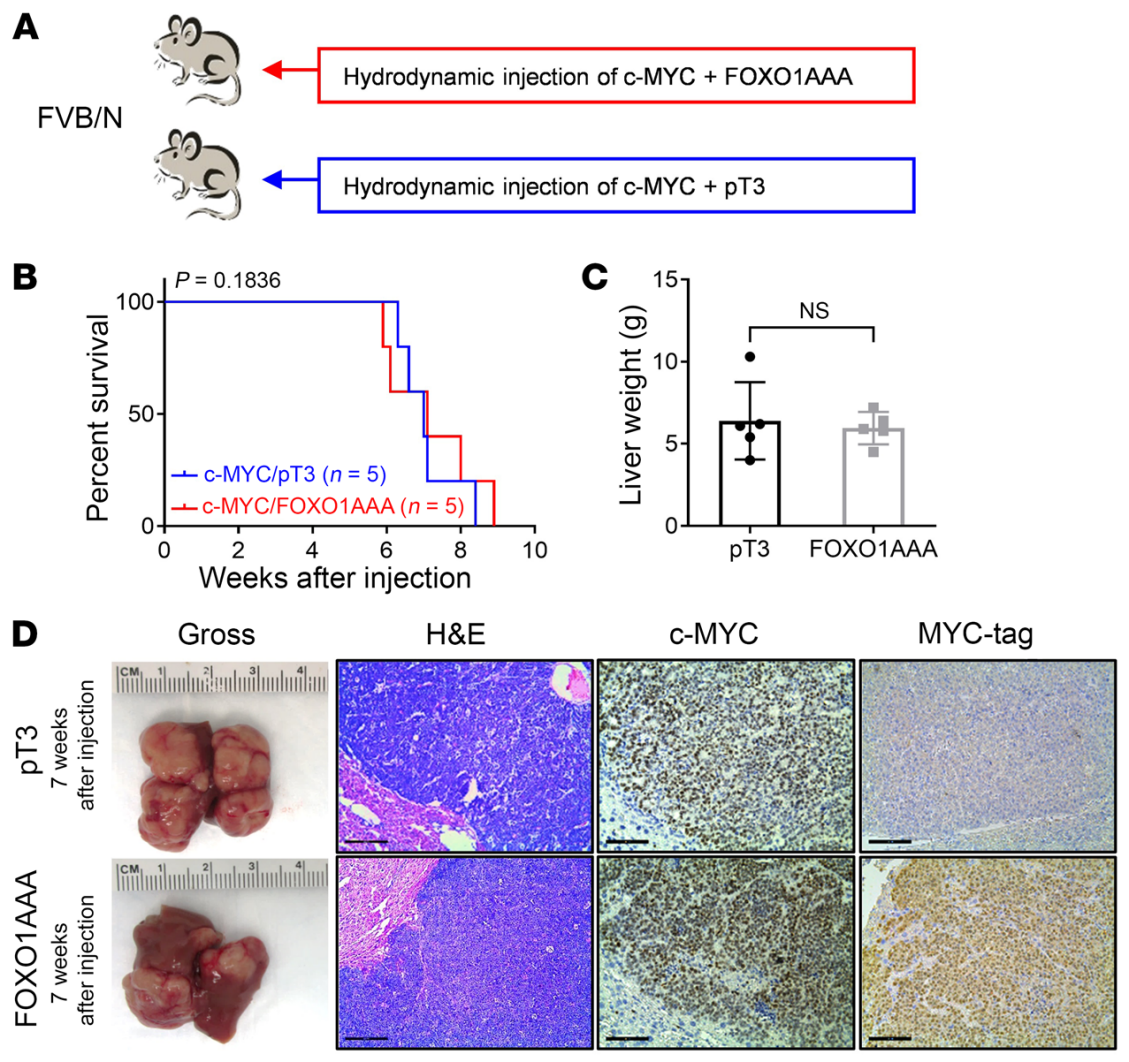
Lack of effect of FOXO1 activation on c-MYC–induced hepatocarcinogenesis. (**A**) Study design. *FVB/N* mice were hydrodynamically injected with plasmid mixtures of c-MYC and a constitutively active mutant of FoxO1 (FoxO1AAA) in a pT3-EF1α backbone with MYC-tag (c-MYC/FoxO1AAA, *n* = 5). Control mice were hydrodynamically injected with c-MYC/MCL1 and pT3-EF1α empty vector (c-MYC/pT3, *n* = 5). Mice were monitored for tumor development and euthanized when moribund tumors developed or until the end of the observation period. (**B**) Survival curve for mice in both groups. A Kaplan-Meier comparison was performed; *P* = 0.1836. (**C**) Comparison of liver weights between the 2 groups. Data are presented as the mean ± SD. A 2-tailed Student’s *t* test was used to determine significance. (**D**) Representative macroscopic images of livers, H&E stainings, and immunohistochemical staining for c-MYC and MYC-tag. Scale bars: 200 μm (H&E); 100 μm (c-MYC and MYC-tag).

As there are multiple FOXO family isoforms and FOXO3 and -4 were also inactivated in the c-MYC tumors (Supplemental Figure 1, A and B), we could not exclude the possibility that other FOXOs compensated for the loss of *Foxo1* tumor suppressor in the double-KO studies. Therefore, we overexpressed the c-MYC oncogene with a MYC-tagged constitutively active form of FOXO1 (FOXO1AAA). Given the common DNA-binding motifs of FOXO family members, it has been suggested that FOXO1AAA activates genes that are regulated by other FOXO members (18, 19). Additional mice were injected with c-MYC and pT3-EF1α empty vector as the control (Figure 2A). Consistent with the results from the *Foxo1* *Rictor* double-KO studies, both c-MYC/FOXO1AAA and c-MYC/pT3-EF1α mice developed a high tumor burden and had to be euthanized by 6–9 weeks after injection, suggesting that activated FoxO1 did not improve the survival of c-MYC mice (Figure 2B). There was no significant difference in tumor burden, as revealed by liver weights between the c-MYC/FOXO1AAA and c-MYC/ pT3-EF1α cohorts (Figure 2C). Gross images and histological analysis showed that c-MYC/FOXO1AAA mice had tumor burdens and histology similar to those of the control group. The staining for MYC-tag confirmed the overexpression of FOXO1AAA in c-MYC/FOXO1AAA HCC (Figure 2D). Western blot analysis also confirmed the expression of FOXO1AAA and c-MYC in liver tumors. Additional downstream components of AKT and mTORC1 signaling (TSC2, p-PRS6, and p-4EBP1) did not differ in protein levels between c-MYC/FOXO1AAA mouse liver tissues and those of controls. The expression of p-AKT^S473^ increased after FOXO1 activation, indicating the feedback activation of the mTORC2 pathway (Supplemental Figure 2A). To further strengthen our findings, we induced overexpression of the constitutively active form of FOXO3 (FOXO3AAA), which is known to regulate cell death and the cell cycle in the liver (20), together with c-MYC (Supplemental Figure 3A). Consistently, we found that FOXO3AAA also had a limited effect on the development of c-MYC tumors (Supplemental Figure 3). Overall, the data show that overexpression of activated FOXOs did not inhibit liver tumor development in c-MYC mice, suggesting a limited role of the FOXO pathway in c-MYC–dependent hepatocarcinogenesis.

While FOXO proteins may possess limited roles in promoting or delaying c-MYC–driven HCC formation, they might still function by modulating molecular features of the tumor cells. To test this hypothesis, we performed RNA-Seq analysis of c-MYC/FOXO1AAA and c-MYC/pT3 tumors as well as WT normal liver tissues. We found that known FOXO1 target genes were constantly upregulated in c-MYC/FOXO1AAA mouse HCC when compared with c-MYC/pT3 tumors (Supplemental Figure 2B), confirming the reliability of the RNA-Seq studies. Further analysis revealed the distinct gene expression patterns of c-MYC/FOXO1AAA and c-MYC/pT3 HCCs (Supplemental Figure 4). Specifically, we identified 735 genes upregulated in the c-MYC/pT3 group compared normal liver, but their expression was downregulated by FOXO1AAA expression (Supplemental Figure 5, A and C and Supplemental Table 1). In addition, 1,206 genes were downregulated in c-MYC/pT3 mouse HCC when compared with the normal liver tissues, but they were upregulated by FOXO1AAA expression (Supplemental Figure 5, B and D and Supplemental Table 1). Intriguingly, many of these genes were related to various metabolic pathways (Supplemental Figure 5), suggesting that FOXO proteins might be critical regulators of HCC metabolism. Thus, the genomic analyses strongly suggest that, while FOXO1AAA might not affect c-MYC cancer development per se, but it could modify the gene expression patterns, especially metabolic pathway genes, of the tumor.

### TSC/mTORC1 is the major downstream effector of AKT along c-MYC–driven hepatocarcinogenesis.

**Figure 3:**
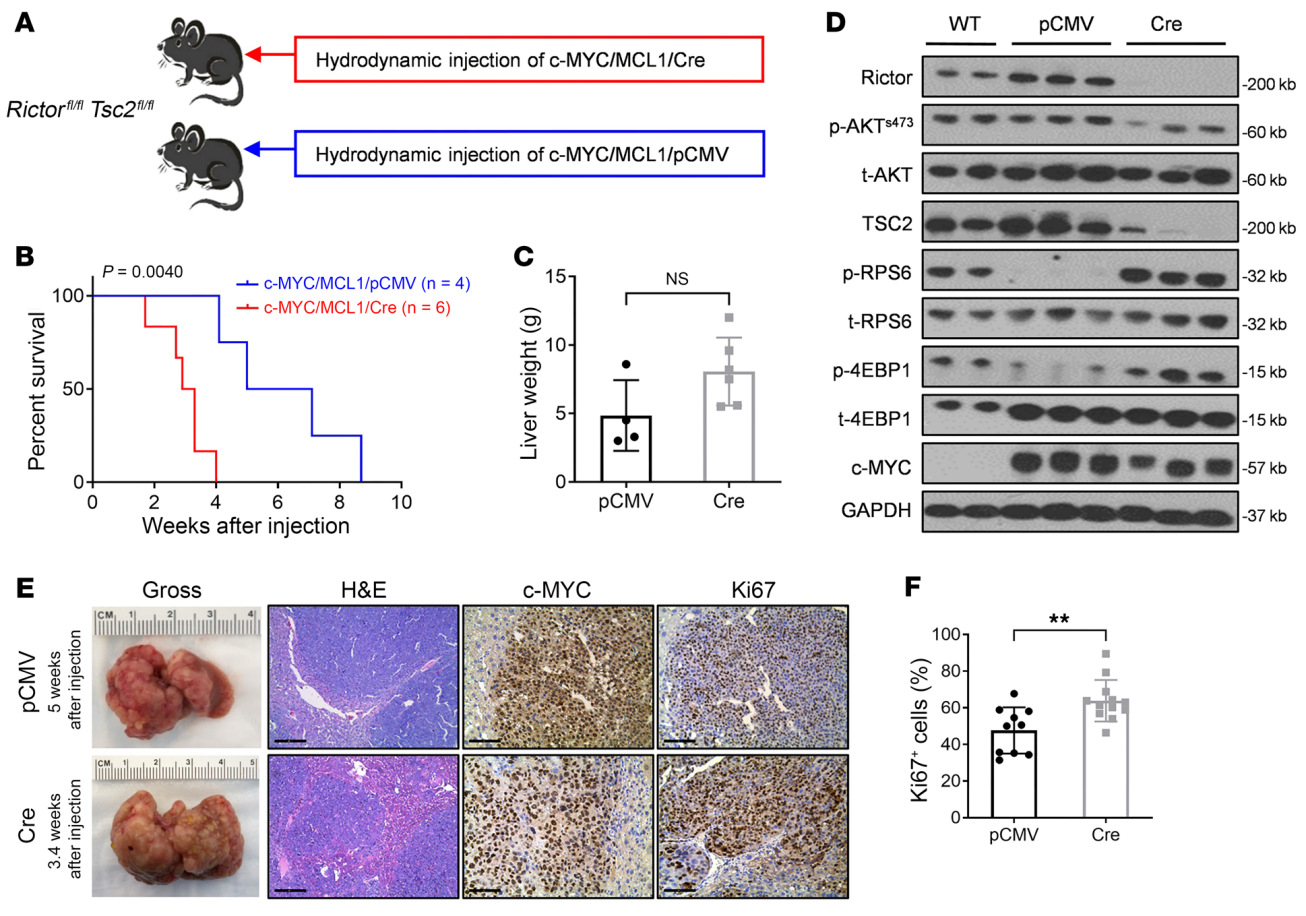
Compensation of the tumor-inhibitory effects of mTORC2 by *Tsc2* deletion. (**A**) Study design. *Rictor^fl/fl^* *Tsc2^fl/fl^* conditional-KO mice were hydrodynamically injected with plasmid mixtures of c-MYC/MCL1 and Cre recombinase in a pCMV backbone (c-MYC/MCL1/Cre, *n* = 4). Control mice were hydrodynamically injected with c-MYC/MCL1 and pCMV empty vector (c-MYC/ MCL1/pCMV, *n* = 6). Mice were monitored for tumor development and euthanized when moribund tumors developed or until the end of the observation period. (**B**) Survival curve for mice in both groups. A Kaplan-Meier comparison was performed; *P* = 0.0040. (**C**) Comparison of liver weights between the 2 groups. Data are presented as the mean ± SD. A 2-tailed Student’s *t* test was used to determine significance. (**D**) Western blot results show expression levels of Rictor, TSC2, and other proteins in the mTORC2/AKT cascades. (**E**) Representative macroscopic images of the liver, H&E stainings, and immunohistochemical staining for Ki67 and c-MYC. Scale bars: 200 μm (H&E); 100 μm (Ki67 and c-MYC). (**F**) Quantification results of the percentage of Ki67^+^ cells in the 2 groups. Data are presented as the mean ± SD. **P < 0.01, by 2-tailed Student’s *t* test.

TSC2 is directly phosphorylated and inactivated by AKT, which results in mTORC1 activation (21, 22). Hence, the ablation of *Tsc2* would lead to persistent activation of the mTORC1 signaling pathway. Our previous study revealed that the mTORC1 pathway was activated and required for c-MYC–induced HCC initiation (16). Here, we aimed to determine whether TSC/mTORC1 is the major downstream effector of AKT along c-MYC–dependent HCC formation. To test this hypothesis, we asked whether loss of *Tsc2* is sufficient to rescue loss of the tumor-inhibiting effects of *Rictor*. We generated *Rictor^fl/fl^* *Tsc2^fl/fl^* double-conditional-KO mice. Subsequently, c-MYC, MCL1, and pCMV-Cre or pCMV plasmids were injected into the mice (Figure 3A). We discovered that injection of c-MYC/MCL1/Cre into *Rictor^fl/fl^* *Tsc2^fl/fl^* mice induced a lethal tumor burden within 1.7–4.0 weeks after injection, whereas in control mice, a fatal tumor burden occurred 4.1–8.7 weeks after injection (Figure 3B). Deletion of *Rictor/Tsc2* also resulted in an increased tumor burden, as shown in liver weight compared with the control group (Figure 3C). At the molecular level, Western blot analysis demonstrated that Rictor and TSC2 were successfully knocked out in Cre-injected mouse liver tissues. Also, p-AKT^S473^ levels were decreased, confirming the inactivation of mTORC2. The downstream effectors of mTORC1, including p-RPS6 and p-4EBP1, were expressed at higher levels, supporting the activation of mTORC1 (Figure 3D). Higher proliferation rates were detected throughout the liver of c-MYC/MCL1/Cre mice, as revealed by Ki67 staining (Figure 3, E and F). The results indicate that the deletion of *Tsc2* fully rescued c-MYC–driven hepatocarcinogenesis in *Rictor*-deficient mice.

The findings above suggest that loss of TSC1 or TSC2 may accelerate c-MYC–driven HCC development. Thus, we investigated whether such phenotypes could be observed in human HCC samples. We retrieved the Cancer Genome Atlas–Liver Hepatocellular Carcinoma (TCGA-LIHC) dataset (23) and analyzed the correlation between the c-MYC activation status and *TSC1/2* mutation status in human HCC. To reflect c-MYC activation status, 30 well-known downstream target genes of c-MYC were used as the c-MYC activation signature (Supplemental Figure 6A). We found that 52.4% of *TSC1/2*-mutant HCC samples had an activated c-MYC status. In contrast, 26.1% of WT samples showed c-MYC activation (Supplemental Figure 6B). HCCs with high c-MYC activation displayed a markedly higher *TSC1/2* mutation rate, suggesting the important role of TSC/mTORC1 during c-MYC tumor development.

Next, we further determined whether the ablation of *Tsc2* alone could also accelerate c-MYC tumor development without MCL1. Thus, we injected c-MYC/Cre or pCMV into *Tsc2^fl/fl^* mice (on the *FVB/N* background) (Supplemental Figure 7A). The c-MYC/pCMV–injected mice developed a lethal tumor burden and were euthanized within 8.9–13.0 weeks after injection. In comparison with the control mice, all c-MYC/Cre–injected mice developed a lethal burden of liver tumors within 3.4 to 6.0 weeks after injection (Supplemental Figure 7B). The c-MYC/Cre mice displayed a higher tumor burden than did the c-MYC/pCMV control mice (Supplemental Figure 7C). Loss of TSC2 protein was confirmed by Western blot analysis. High expression of p-RPS6, the downstream effector of mTORC1, as well as increased expression of cleaved caspase 3, was detected in liver tissues from c-MYC/Cre–injected *Tsc2^fl/fl^* mice (Supplemental Figure 7D). No histopathological alterations were detected in liver sections of c-MYC/Cre–injected mice or control mice (Supplemental Figure 7E).

Our previous study suggests that mTORC1 is necessary for c-MYC–driven HCC initiation (16). We also investigated whether mTORC1 is required for the progression of c-MYC HCC. For this purpose, we used a tamoxifen-inducible CreERT2 system to create a conditional *Raptor*-KO mouse HCC model. We used a transposase-based vector, pT3-TTR-CreERT2, which incorporates the TTR-CreERT2 transgene under the control of hepatocyte-specific transthyretin (TTR) promoter. We induced coexpression of c-MYC, MCL1, and pT3-TTR-CreERT2 in *Raptor^fl/fl^* mouse liver by hydrodynamic injection. Two weeks after injection, tumor nodules were observed, and the tumor-bearing mice were subsequently treated with tamoxifen or a vehicle via intraperitoneal injection (Supplemental Figure 8). Tamoxifen treatment activated Cre recombinase and deleted Raptor in tumor cells, allowing us to investigate the role of mTORC1 in the already formed HCCs. Tamoxifen treatment significantly improved the overall survival rate (Supplemental Figure 9). Interestingly, large areas of necrosis were frequently observed in tamoxifen-treated livers (Supplemental Figure 10). By the end of the observation, the tamoxifen-treated group only developed 3 small individual tumor nodules in this mouse cohort. IHC revealed the expression of RAPTOR protein in all the tumor nodules (Supplemental Figure 11). Since we have previously proved the efficiency of the TTR-Cre-ERT2 system (24), we reasoned that such tumor nodules were escapers. These findings prove that *Raptor* ablation in tumor cells induces notable liver tumor regression in c-MYC mice.

In summary, the mouse and human studies suggest that TSC/mTORC1 is the main effector downstream of mTORC2/AKT1 in c-MYC–driven HCC and that loss of TSC accelerates c-MYC–induced liver tumor formation.

### p70S6K/RPS6 and 4EBP1/eIF4E cascades regulate distinct pathways during c-MYC tumorigenesis.

**Figure 4:**
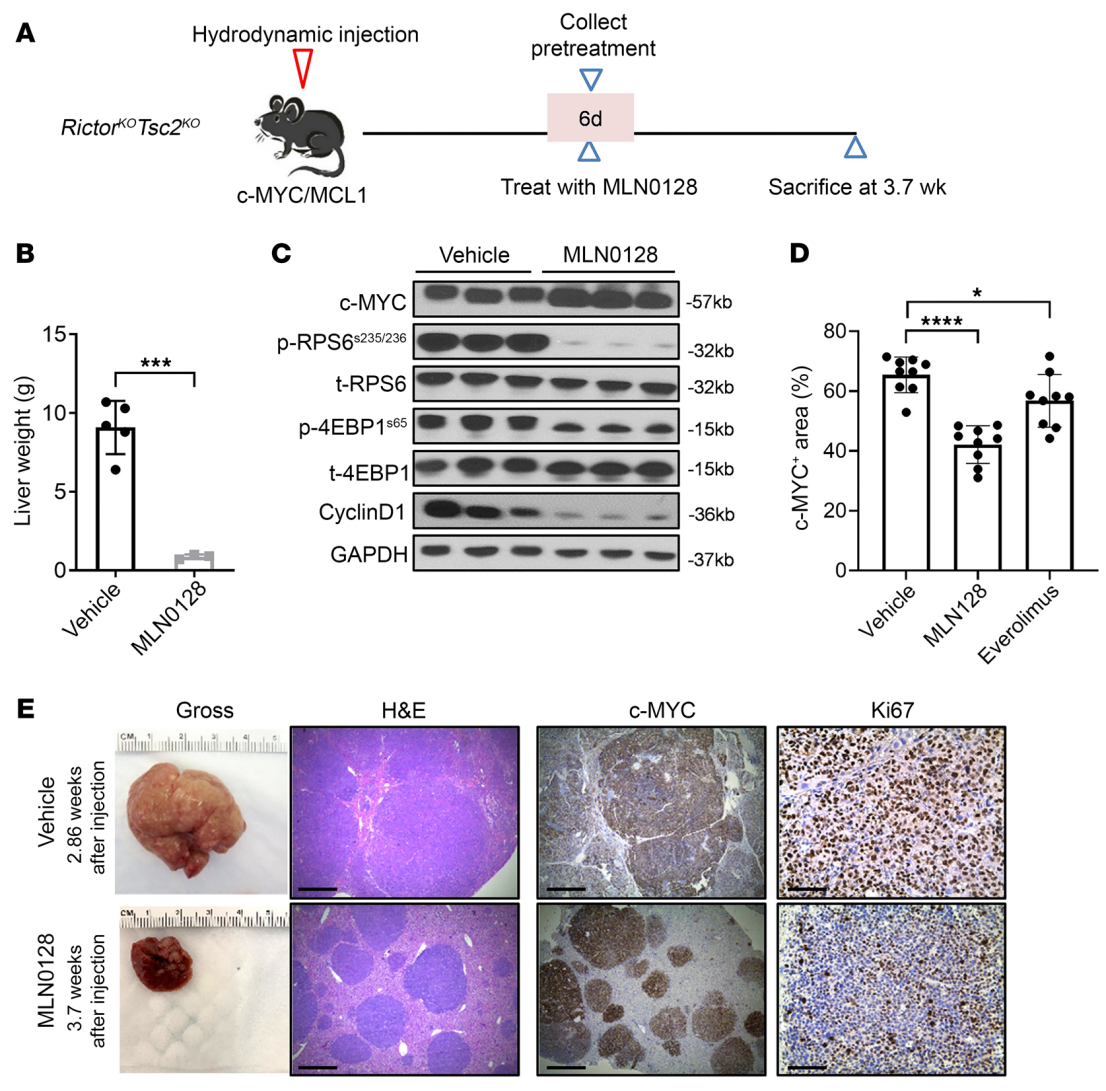
Inhibition of c-MYC/MCL1/*Rictor^KO^* *Tsc2^KO^* tumor growth by MLN0128 treatment. (**A**) Study design. The c-MYC/MCL1/*Rictor^KO^Tsc2^KO^* murine tumor model was established by hydrodynamic injection. Six days after injection, 1 group of mice (*n* = 3) was sacrificed, and mouse livers were harvested for analysis as the pretreatment group. The remaining mice were treated with MLN0128 (*n* = 3) or vehicle (*n* = 5) for 3 weeks. Subsequently, all mice were sacrificed for analysis. (**B**) Comparison of liver weights between the MLN0128 and vehicle-treated groups. (**C**) Western blot analysis showing the levels of c-MYC, cyclin D1, and key molecules downstream of mTORC2 (*n* = 3, 3). GAPDH was used as the loading control. (**D**) Comparison of c-MYC^+^ areas in the MLN0128-, everolimus-, and vehicle-treated groups. (**E**) Representative images of gross views of the liver, H&E stainings, and immunohistochemical staining for Ki67 and c-MYC. Scale bars: 500 μm (H&E and c-MYC); 100 μm (Ki67). Data are presented as the mean ± SD. *P < 0.05, ***P < 0.001, and ****P < 0.0001, by 2-tailed Student’s *t* test.

According to our previous research, both the p70S6K/RPS6 and 4EBP1/eIF4E cascades operate downstream of mTORC1 in HCC (25). These findings were further corroborated in c-MYC/MCL1/*Rictor^KO^* *Tsc2^KO^* liver tumor lesions induced with c-MYC/MCL1/Cre in *Rictor^fl/fl^* *Tsc2^fl/fl^* mice. We observed that the inhibition of p70S6K/RPS6 using everolimus or the use of 4EBP1A4, the unphosphorylatable form of 4EBP1, blocked tumor development in these mice (Supplemental Figures 12 and 13). Subsequently, we investigated the pathways regulated by p70S6K/RPS6 and 4EBP1/eIF4E cascades in c-MYC–driven HCC. However, we discovered that tumors still developed in mice overexpressing 4EBP1A4 (Supplemental Figure 14), probably due to compensatory mechanisms that led to the resistance to 4EBP1/eIF4E pathway inhibition over the long-term course of tumor growth. Consequently, we pursued an alternative approach. We treated *c-MYC/MCL1/Rictor^KO^* *Tsc2^KO^* HCCs with MLN0128 (Figure 4A). MLN0128 is a pan-mTOR inhibitor that suppresses both mTORC1 and mTORC2. In *c-MYC/MCL1/Rictor^KO^* *Tsc2^KO^* mouse tumors, where *Rictor* is lost, mTORC2 is effectively inactivated, making MLN0128 the ideal inhibitor of mTORC1, including both the p70S6K/RPS6 and 4EBP1/eIF4E pathways. Indeed, we found that MLN0128 treatment effectively suppressed *c-MYC/MCL1/Rictor^KO^* *Tsc2^KO^* tumor growth, and it was more effective than the p70S6K/RPS6 inhibitor everolimus (Figure 4).

**Figure 5:**
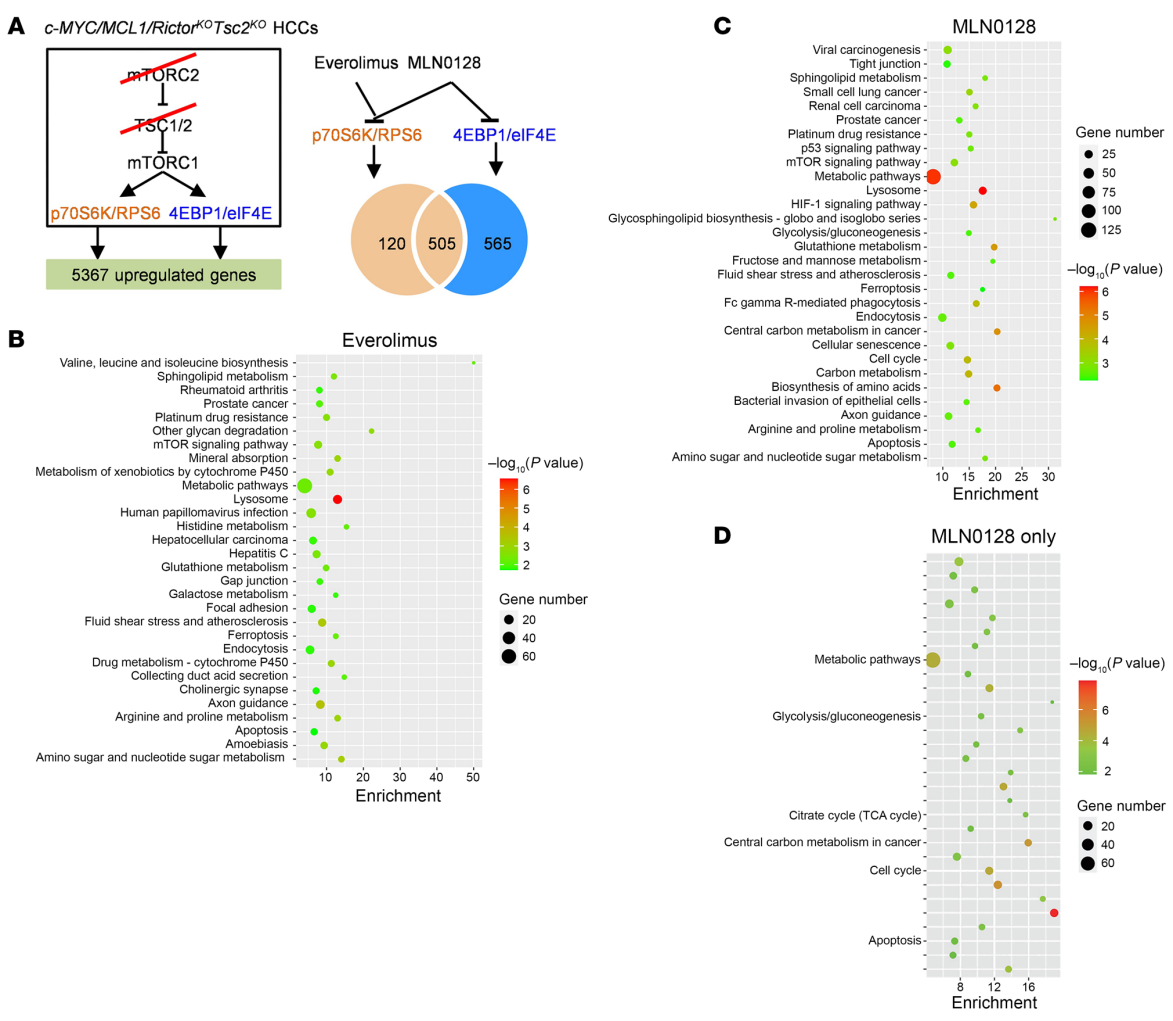
Analysis of p70S6K/RPS6 and 4EBP1/eIF4E downstream target genes. (**A**) Study design. Schematic diagram showing the signaling pathways involved in the c-MYC/MCL1/*Rictor^KO^* *Tsc2^KO^* tumors (left panel). RNA-Seq was performed on the normal liver tissue, and c-MYC/MCL1/*Rictor^KO^* *Tsc2^KO^* tumors were treated with vehicle, everolimus, or MLN0128 (*n* = 3, 3, 3). Venn diagram shows the number of DEGs. (**B**) KEGG analysis of the DEGs downregulated by MLN0128. (**C**) KEGG analysis of the DEGs downregulated by everolimus. (**D**) KEGG analysis of the DEGs downregulated by MLN0128, but not everolimus.

To identify the gene expression patterns regulated by the p70S6K/RPS6 and 4EBP1/eIF4E cascades, we treated *c-MYC/MCL1/Rictor^KO^* *Tsc2^KO^* mouse HCCs with everolimus (to inhibit p70S6K/RPS6) or MLN0128 (to inhibit both p70S6K/RPS6 and 4EBP1/eIF4E) for 3 days (Figure 5A). We performed RNA-Seq experiments. Normal liver tissues and vehicle-treated tumors were used as the controls. We focused on genes upregulated in mouse HCC samples and downregulated by everolimus and/or MLN0128. Specifically, we found that 5,367 genes were upregulated (fold change >1.5; adjusted *P* value [*P* adj] < 0.05) in tumor tissues compared with normal livers. Among them, 625 genes were downregulated upon everolimus and MLN0128 treatment, implying that these genes are presumably downstream molecules regulated by the p70S6K/RPS6 pathway. In addition, 565 genes were downregulated by MLN0128 but not by everolimus, indicating that these genes are likely regulated by the 4EBP1/eIF4E cascade (Figure 5A).

Kyoto Encyclopedia of Genes and Genomes (KEGG) enrichment analysis revealed that p70S6K/RPS6 or 4EBP1/eIF4E regulated different biological processes (Figure 5, B and C). It has been previously established that p70S6K/RPS6 is a pivotal regulator of tumor cell metabolism (26). On the other hand, the genes or pathways regulated by 4EBP1/eIF4E are not well defined. Therefore, we focused on analyzing the differentially downregulated genes affected by MLN0128 treatment only, i.e., 4EBP1/eIF4E pathway genes. These genes were enriched in the cell cycle and metabolic pathways required for cell proliferation, including glycolysis, the citrate cycle, and carbon metabolism (Figure 5D and Supplemental Figure 9A). Western blot analysis confirmed the decreased expression of the glycolysis-related proteins LDHA/C and PKM1 in MLN0128-treated tumors (Supplemental Figure 9B).

As a primary downstream target of mTORC1 signaling, the 4EBP1/eIF4E cascade promotes c-MYC–driven HCC development by regulating tumor cell proliferation.

### CENPM is the pivotal target gene downstream of mTORC1 in c-MYC HCC.

**Figure 6:**
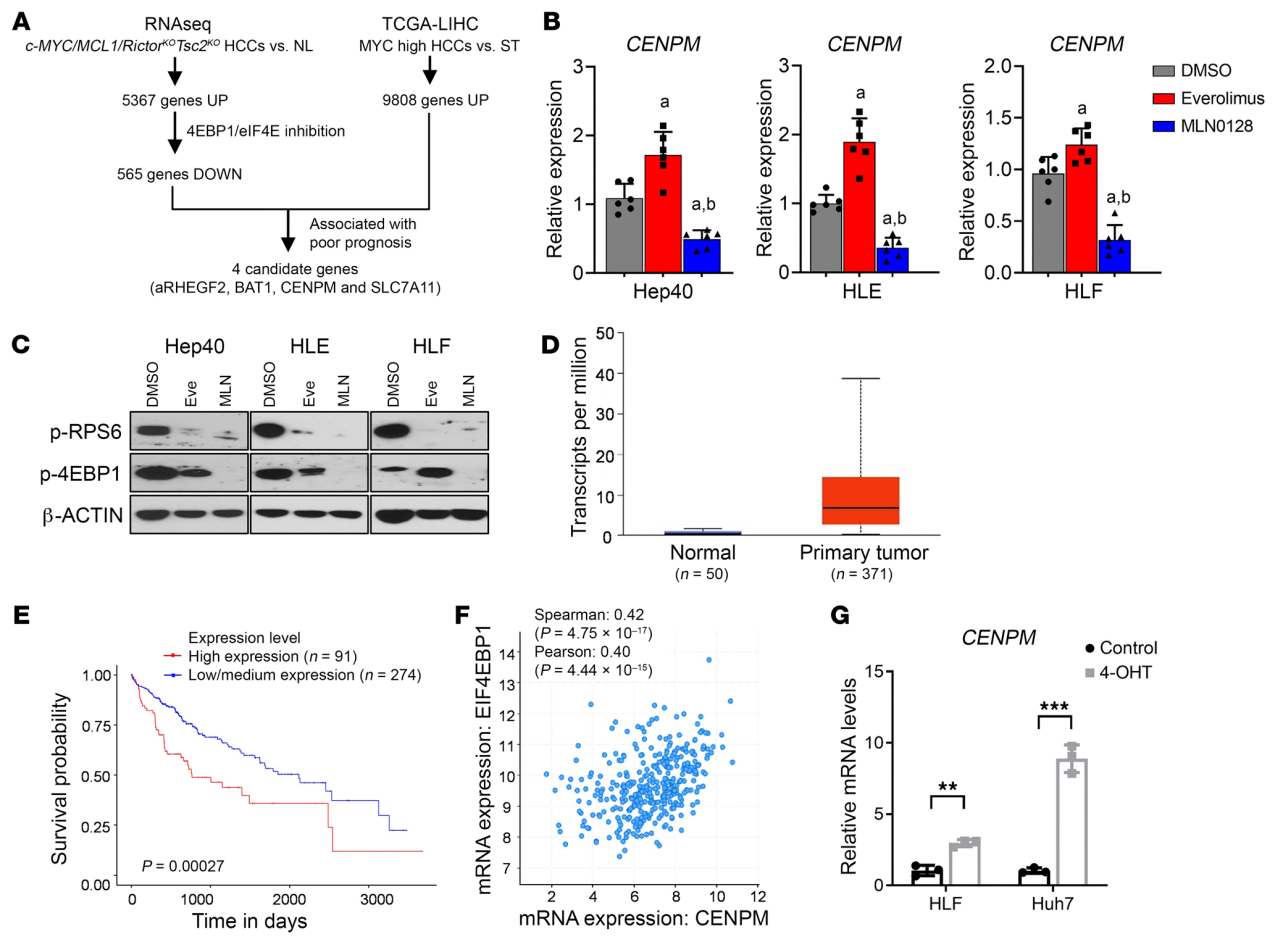
CENPM is a central effector downstream of 4EBP1/eIF4E signaling in c-MYC HCCs. (**A**) Schematic diagram illustrating the identification of target genes regulated by 4EBP1/eIF4E signaling in c-MYC HCCs. UP, upregulated; DOWN, downregulated. (**B**) qPCR results showing *CENPM* mRNA levels in the 3 HCC cell lines (Hep40, HLE, and HLF) treated with DMSO, everolimus, or MLN0128 (*n* = 6, 6, 6). Data are presented as the mean ± SD. At least *P* < 0.05, by Tukey-Kramer test. a, versus DMSO; b, versus everolimus. (**C**) Western blot analysis showing the levels of p-RPS6 and p-4EBP1 in HCC cells treated with DMSO, MLN0128, or everolimus. β-Actin was used as the loading control. (**D**) Expression of CENPM in the human HCC samples and normal liver based on the TCGA-LIHC dataset. Data are presented as the mean ± SD. *P* < 1 × 10^–12^, by 2-tailed Student’s *t* test. (**E**) Survival curve for the patients with HCC with high *CENPM* expression compared with those with low/medium *CENPM* expression (from https://ualcan.path.uab.edu/). Samples were categorized into 2 groups: high expression (with TPM values above the upper quartile) and low/medium expression (with TPM values below the upper quartile). A Kaplan-Meier comparison was performed; *P* = 0.00027. (**F**) Correlation between *CENPM* and *EIF4EBP1* mRNA levels in human HCCs. (**G**) qPCR results showing *CENPM* mRNA levels in the MYC-ER–transfected HCC cell lines (Hep40 and HLE) after treatment with DMSO or 4OHT (*n* = 3, 3). Data are presented as the mean ± SD. **P < 0.01 and ***P < 0.001, by 2-tailed Student’s *t* test.

Next, we searched for potential downstream effectors of the mTORC1 cascade, especially those downstream of the 4EBP1/eIF4E pathway, in c-MYC–induced liver lesions. We identified the genes upregulated in tumor tissues and downregulated by MLN0128 treatment but not by everolimus treatment (based on RNA-Seq data). Among them, 30 genes were also overexpressed in c-MYC mouse liver tumors based on our previous microarray analysis data (16). Each gene was searched in the TCGA-LIHC dataset for any possible link to HCC. Four genes were upregulated in human HCCs and are associated with poor prognosis of the patients: ARHEGF2, BAT1, CENPM, and SLC7A11 (Figure 6A). Subsequently, we analyzed their expression in human HCC cell lines upon Everolimus or MLN0128 treatment. Of note, only CENPM was consistently downregulated by MLN0128 (Figure 6B and Supplemental Figure 15A). Western blot results showed that everolimus only inhibited the activation of RPS6 signaling, while MLN0128, a pan-mTOR inhibitor, inhibited both RPS6 and 4EBP1 pathways downstream of mTORC1 (Figure 6C). These results suggested that CENPM is a 4EBP1/eIF4E target downstream of mTORC1. CENPM is one of the critical components of a complex that allows kinetochore protein assembly, mitotic progression, and chromosome segregation. In TCGA database, *CENPM* mRNA expression was substantially upregulated in human HCC tissues compared with surrounding tissues (Figure 6D). In addition, low expression levels of *CENPM* were linked to a better prognosis in human HCC cohorts (Figure 6E). *CENPM* expression was also positively correlated with *EIF4EBP1*, the major downstream effector of mTORC1 (Figure 6F). In addition, we also found that *CENPM* levels positively correlated with levels of MYC activation (Supplemental Figure 15B). Thus, we hypothesized that *CENPM* might be a candidate target gene downstream of mTORC1/4EBP1 in c-MYC–induced HCC.

To investigate the role of CENPM in c-MYC HCC progression, we infected 2 HCC cell lines (HLF and Huh7) with 4-hydroxytamoxifen–inducible (4OHT-inducible) c-MYC lentivirus (p-Lenti-4OHT-cMyc-ER). As our previous studies revealed (15, 16), c-MYC is expressed at low levels in HLF and Huh7 cell lines. Upon 4OHT treatment, c-MYC expression was induced, and *CENPM* mRNA levels were concomitantly upregulated in HLF and Huh7 cells (Figure 6G).

**Figure 7:**
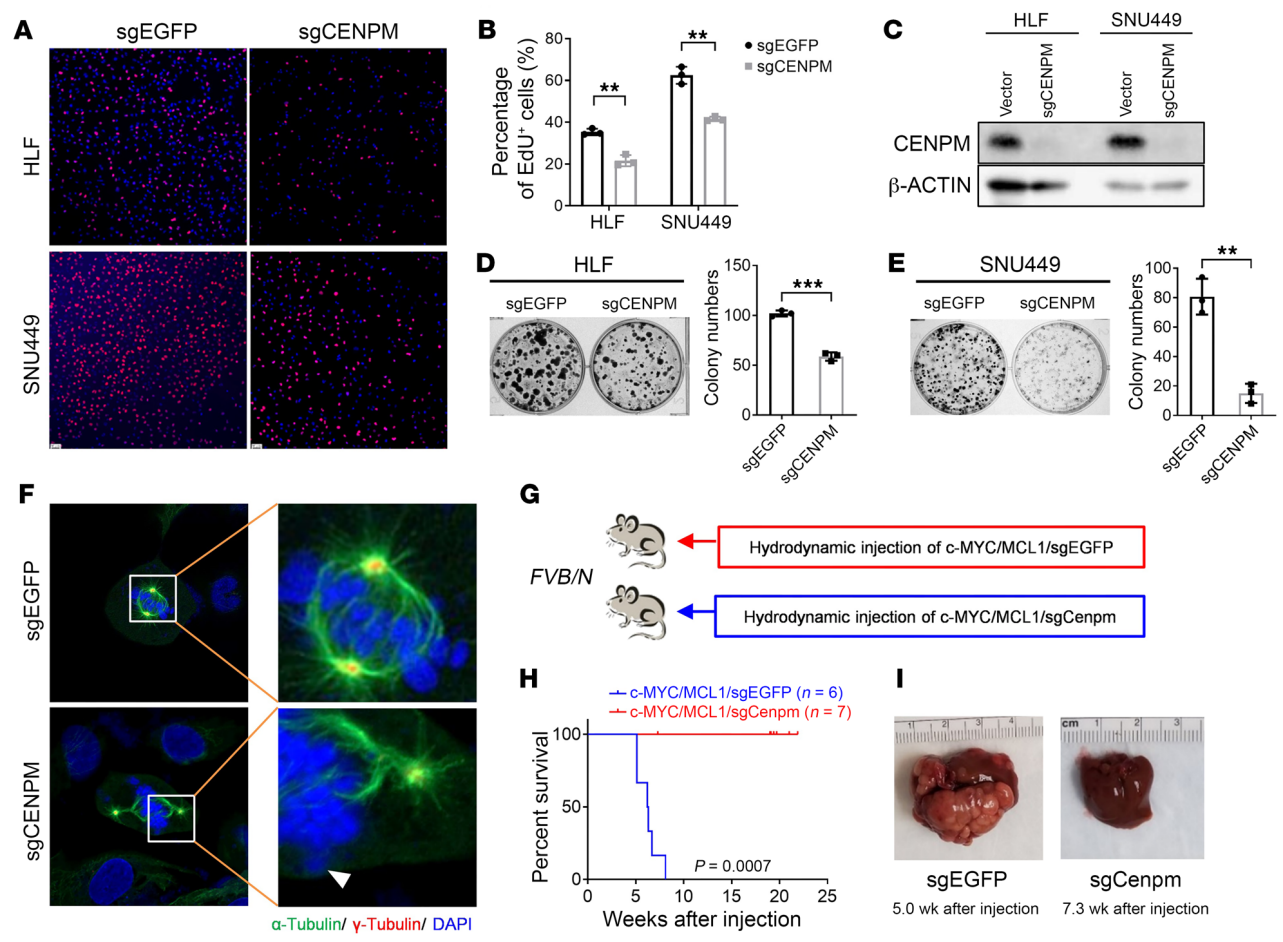
Targeting CENPM suppresses HCC cell proliferation and c-MYC–induced hepatocarcinogenesis. (**A** and **B**) Representative images (**A**) and quantification (**B**) of EdU staining in HLF and SNU449 cells transfected with sgEGFP or sgCENPM (*n* = 3, 3). Scale bars: 50 μm. (**C**) Western blot analysis confirming the knockout of CENPM in the HCC cells. β-Actin was used as the loading control. (**D** and **E**) Representative images and quantification of the colony formation assay in sgEGFP- or sgCENPM-transfected HLF (**D**) and SNU449 (**E**) cells. (**F**) Representative images of immunofluorescence staining for α-tubulin (indicating microtubule, kinetochore, or spindle fibers), γ-tubulin (centrosome), and DAPI (indicating chromosomes) in CENPM-KO cells and control cells during mitosis. Lagging chromosomes (indicated by the white arrowhead) or mis-segregations were observed in almost all the CENPM-KO cells. Original magnification, ×1,000 and ×4,300 (enlarged insets). (**G**) Study design. *FVB/N* mice were hydrodynamically injected with plasmid mixtures of c-MYC/MCL1 and CRISPR plasmid with gRNA targeting the mouse *Cenpm* genome (c-MYC/MCL1/sgCenpm, *n* = 7). Control mice were hydrodynamically injected with c-MYC/MCL1 and sgEGFP (c-MYC/MCL1/sgEGFP, *n* = 6). Mice were monitored for tumor development and euthanized when moribund tumors developed or until the end of the observation period. (**H**) Survival curve for mice in both groups. A Kaplan-Meier comparison was performed; *P* = 0.0007. (**I**) Representative macroscopic images of livers from both groups. Data are presented as the mean ± SD. **P < 0.01 and ***P < 0.001, by 2-tailed Student’s *t* test (**B**, **D**, and **E**).

To further characterize the effect of CENPM on c-MYC HCC tumor development, we silenced CENPM in human HCC cell lines using siRNA (siCENPM). The cell viability was strongly inhibited by siCENPM, as revealed by EdU staining (Supplemental Figure 16). Consistently, based on Dependency Map (DepMap; https://depmap.org/portal/) studies, sgRNA against CENPM also led to considerable HCC cell growth inhibition in all 22 human HCC cells tested (Supplemental Figure 17). To validate this observation, 2 human HCC cell lines, HLF and Hep40, were also transfected with sgCENPM (human) lentivirus. Of note, human HCC cell proliferation was significantly inhibited, as EdU and colony formation assays showed (Figure 7, A, B, D, and E). In addition, CENPM protein expression was remarkably reduced (Figure 7C). Moreover, immunofluorescence-based microscopy analysis of CENPM-KO single cells indicated the influence of this gene on chromosome segregation in HCC cells. Specifically, lagging chromosomes or mis-segregations were observed in the CENPM-KO cells during mitosis, likely due to abnormal kinetochore proteins caused by CENPM loss (Figure 7F and Supplemental Figure 18).

Next, to investigate the role of CENPM in c-MYC–induced hepatocarcinogenesis in vivo, we induced coexpression of c-MYC, MCL1, and CRISPR-Cas9–mediated KO plasmid (sgCenpm) in mouse liver by hydrodynamic injection, whereas c-MYC, MCL1, and sgEGFP plasmids were injected into additional mice as controls (Figure 7G). Strikingly, *Cenpm* deletion completely suppressed c-MYC HCC formation in mice (Figure 7, H and I). Indeed, 20 weeks after injection, none of the c-MYC/MCL1/sgCenpm–injected mice developed liver tumors. As an additional control for this experiment, sgRNA against 3 genes previously selected as candidate mTORC1 targets, *Arhegf2*, *Bat1*, and *Scl7a11*, were coinjected with c-MYC and MCL1. However, none of them delayed c-MYC–driven HCC development, indicating the importance of targeting Cenpm to effectively blunt c-MYC–dependent hepatocarcinogenesis (Supplemental Figure 19). To further substantiate the effective deletion of the *Cenpm* gene, we transfected a sgCenpm lentivirus construct into HCC3-4 cells, a mouse HCC cell line with c-MYC activation. The Tracking of Indels by Decomposition (TIDE) assay result confirmed the effectiveness of our CRISPR/Cas9 system (Supplemental Figure 20A). Consistent results were also found in the c-MYC/sg*Cenpm* liver tissues that were harvested at an early stage when c-MYC^+^ cells still existed (Supplemental Figure 20B).

In summary, our study strongly suggests that CENPM is a critical downstream target of mTORC1 signaling in c-MYC HCC initiation.

## Discussion

The present study systematically dissected the signaling pathways downstream of mTORC2/AKT1 in c-MYC–dependent hepatocarcinogenesis. The first key conclusion from our in vivo genetic investigation is that FOXOs, including FOXO1, the major FOXO family member in the liver, have limited relevance in regulating AKT1 signaling–mediated, c-MYC–driven HCC in mice. This finding contrasts with previous studies suggesting that AKT regulates liver physiology and pathophysiology by predominantly controlling FOXO1. For instance, it has been shown that liver-specific deletion of *Akt1* and *Akt2* led to glucose intolerance and insulin resistance. These defects could be normalized by codeleting *Foxo1* in the liver (13). Loss of *Akt1/Akt2* led to liver regeneration defects. In *Akt1/Akt2/Foxo1* triple–liver-specific–KO mice, liver regeneration was restored, as seen in the WT mice (11). Similarly, mice with liver-specific deletion of *Akt1/Akt2* developed HCC over the long term. Liver tumor development could be abolished entirely in the *Akt1/Akt2/Foxo1* triple–liver-specific–KO mice (12). All these studies suggest that FOXO1 is a key molecule downstream of AKT signaling in the liver. In striking contrast to these data, our current study shows that loss of FOXO1 failed to restore c-MYC–driven HCC development in the absence of the mTORC2/AKT cascade. The results highlight that FOXO1 or FOXO family members may have distinct functions depending on different pathological stimuli. The present findings also indicate that suppressing FOXO family members may have limited value for treating c-MYC–dependent hepatocarcinogenesis.

Our current and previous studies demonstrate that the TSC/mTORC1 cascade is necessary and sufficient for HCC development in c-MYC mice. The data also underline the critical role of the TSC complex in c-MYC–driven hepatocarcinogenesis. This conclusion is further supported by the fact that *TSC1/2*-mutant human HCC samples were enriched in c-MYC–activated tumors (Supplemental Figure 6). In mice, loss of TSC2 significantly accelerated c-MYC–induced liver tumor formation (Supplemental Figure 7). These results also support the possible use of mTOR inhibitors for HCC treatment. However, our RNA-Seq studies indicated that genes downregulated by either MLN0128 or everolimus treatments were enriched in the apoptosis pathway (Figure 4). Specifically, expression levels of several proapoptotic genes (*Bak1*, *Bcl10*, *Card19*, *Casp3*, *Ltbr*, *Noxa*) in these treatment groups were reduced compared with the expression in the vehicle-treated group (Supplemental Figure 21). Therefore, mTORC1 inhibitors might even cause adverse effects by inducing apoptosis in the c-MYC/MCL1 HCC model and in corresponding human tumors. Combining mTORC1 inhibitors with drugs targeting the apoptosis process may be needed for patients with HCC who harbor c-Myc activation.

mTORC1 is known to function through the p70S6K/RPS6 and 4EBP1/eIF4E cascades. It is important to note that both p70S6K/RPS6 and 4EBP1/eIF4E cascades regulate protein translation as their primary function. Nevertheless, eventually, the deregulated protein translation affects different targets. It has been established that p70S6K/RPS6 regulates tumor metabolism, such as de novo lipogenesis. Studies have shown that targeting the deregulated metabolic pathway, such as through deletion of FASN, a major enzyme in de novo lipogenesis, could substantially delay c-MYC HCC development (27). However, the genes or pathways regulated by the 4EBP1/eIF4E cascade in HCC are not well characterized. This is mainly because the 4EBP1/eIF4E cascade predominantly regulates protein translation, which is much more difficult to analyze technically. Here, we chose to use RNA-Seq in combination with everolimus or MLN0128 treatment of *c-MYC/* *MCL1/Ricto^KO^Tsc2^KO^* mouse liver tumors to identify genes or pathways that are indirectly regulated by the 4EBP1/eIF4E cascade. Our study indicates that 4EBP1/eIF4E is the major signaling that modulates tumor cell proliferation and metabolic pathways, such as PKM1 and LDHA/C, that directly contribute to tumor proliferation (Supplemental Figure 14B).

Finally, we identified CENPM as a downstream target of 4EBP1/eIF4E, as its expression was downregulated in c-MYC HCC by MLN0128, but not everolimus (Figure 6B and Supplemental Figure 15A). Nonetheless, the regulation of CENPM by 4EBP1/eIF4E might be indirect. The eIF4E protein plays a crucial role in binding to the 5′ cap structure of mRNA, and its availability often limits translation initiation. 4EBP1 binds to eIF4E and inhibits its function, thereby preventing translation initiation. Activation of the mTOR pathway leads to phosphorylation of 4EBP1, causing its dissociation from eIF4E and promoting translation initiation. Previous evidence indicates that upregulation of CENPM promotes cancer progression through the mTOR signaling pathway (28). In addition, several microRNAs, including miR-214-3p (29), have been implicated in the posttranscriptional regulation of CENPM. Interestingly, miR-214-3p is also involved in regulating 4EBP1 signaling (30). Therefore, the 4EBP1-eIF4E complex might regulate CENPM translation by interacting with microRNAs. However, the precise mechanisms underlying this regulation require further investigation. Furthermore, additional mechanisms might lead to the upregulation of CENPM in c-MYC–driven HCC. Indeed, our bioinformatics analysis and experimental studies demonstrated that CENPM was also a direct transcriptional target of c-MYC (Supplemental Figures 22 and 23), supporting the idea that c-MYC regulates CENPM via multiple mechanisms. Finally, we show that CENPM was an important mediator of c-MYC–induced HCC. Of note, it is unlikely that CENPM is the only key molecule in driving c-MYC–induced HCC formation. Several other proteins, such as TAZ (17), SLC1A5 (16), etc., were also shown to be crucial for c-MYC–driven hepatocarcinogenesis. Nevertheless, the current study suggests that CENPM might be a valuable target for treating c-MYC–driven HCC.

## Methods

### Sex as a biological variable.

Sex was not considered as a biological variable. Both male and females animals were used in this study.

### Constructs and reagents.

The plasmids used in the study, including pT3-elongation factor 1 α (EF1α), MCL1, pT3-EF1α-c-MYC, pT3-EF1α-4EBP1A4, p-CMV, cyclization recombination (Cre), and pCMV/sleeping beauty transposase (SB) have been described in our previous publications (25, 31). The pT3-EF1α-FoxO1AAA plasmid was constructed from pCMV5-Myc-FoxO1AAA (no. 17547, Addgene, deposited by Domenico Accili, College of Physicians and Surgeons of Columbia University, New York, New York, USA). The pT3-TTRpro-CreERT2 plasmid was constructed from pCAG-CreERT2 (no. 14797, Addgene, deposited by Connie Cepko, Harvard Medical School, Boston, Massachusetts, USA) using a standard molecular cloning approach. The pCMV4a-Flag-c-Myc construct was purchased from Addgene (no. 102625, Addgene, deposited by Hening Lin, Cornell University, Ithaca, New York, USA). The pGL3 firefly luciferase reporter vector plasmid (catalog E1751) and the pRL-CMV *Renilla* luciferase control reporter vector plasmid (catalog E2231) were purchased from Promega. To generate the pGL3-CENPM promoter plasmid, a 2000bp DNA fragment of the human CNEPM promoter containing the predicted c-MYC–binding site was subcloned into the pGL3 vector. To generate the pGL3-Motif-Mut plasmid, a 1988bp truncated DNA fragment deleting the putative c-MYC–binding site (CACCACGTGTTC) in the CENPM genome was subcloned into the pGL3 vector. For CRISPR/Cas9-mediated gene deletion, the sgRNA guide sequence was cloned into LentiCRISPRv2 puro (no. 98290, Addgene, deposited by Brett Stringer Laboratory, Griffith University, Brisbane, Australia) or PX330 (no. 42230, Addgene, deposited by Feng Zhang at Massachusetts Institute of Technology, Cambridge, Massachusetts, USA) according to the published protocol (32). The guide RNAs used in the study are listed in Supplemental Table 2. All plasmids were purified using the Endotoxin-free Maxi Prep Kit (MilliporeSigma). MLN0128 (I-3344) and everolimus (E-4040) were from LC Laboratories,, and tamoxifen was purchased from MilliporeSigma.

### Hydrodynamic injection and mouse treatment.

WT *FVB/N* mice were obtained from Charles River Laboratories. *Tsc2^fl/fl^* mice (on the C57BL/6J background), *Rictor^fl/fl^* mice (on the C57BL/6J background), *Foxo1^fl/fl^* mice (on the FVB/NJ background), and *Raptor ^fl/fl^* mice (on the C57BL/6J+N mixed background) were purchased from The Jackson Laboratory. *Rictor^fl/fl^* *Foxo1^fl/fl^* and *Rictor^fl/fl^* *Tsc2^fl/fl^* mice were generated by crossing *Rictor^fl/fl^* mice with *Tsc2^fl/fl^* mice or *Foxo1^fl/fl^* mice, respectively. Hydrodynamic tail vein injection was performed as described previously (33, 34). The plasmid mixture compounds, which induced mouse c-MYC/MCL1 HCC, are provided in Supplemental Table 3.

MLN0128 (I-3344) and everolimus (E-4040) were purchased from LC Laboratories. MLN0128 was first dissolved in 1-methyl-2-pyrrolidinone (NMP) (328634, MilliporeSigma) to make a stock solution of 20 mg/mL, and then 1:100 diluted into 15% polyvinylpyrrolidone (PVP) (81420, MilliporeSigma) to H_2_O. The diluted solution was stored at 4°C in the dark before administration. Everolimus was dissolved in 100% ethanol to make a stock solution of 50 mg/mL and then mixed with 1% PBS to make a 0.2 mg/mL working solution before administration. Tamoxifen (T5648, MilliporeSigma) was dissolved in corn oil for 1 hour in a roller (hybridization oven) at 65°C to make a stock solution of 20 mg/mL. Tamoxifen was warmed for 10 minutes at 65°C before injection.

MLN0128 (1 mg/kg/day), everolimus (1 mg/kg/day), or vehicle was administered daily (6 days a week) over 3 weeks, via oral gavage, starting 6 days after plasmid injection. Mice were sacrificed 3.7 weeks after hydrodynamic injection (3 weeks after treatment). Two weeks after injection of c-MYC/MCL1/TTR-CreERT2 plasmids (when tumor nodules are visible on the liver surface), a group of mice were harvested as a pretreatment cohort, and additional mice were either intraperitoneally injected with corn oil or tamoxifen (9 mg/40 g body weight), 3 times, once every other day. Tamoxifen administration allows activation of the Cre recombinase and the subsequent deletion of Raptor only in TTR promoter (+) HCC tumor cells. Abdominal girth and signs of morbidity or discomfort were monitored for all mice.

### Cell lines, cell culturing, and in vitro experiments.

Three human HCC cell lines (SNU449, HLE, and HLF) were used in this study. The cell lines were obtained from the American Type Culture Collection (ATCC). All cells were authenticated and confirmed to be free of mycoplasma contamination. SNU449 cells were cultured in RPMI-1640, whereas HLE and HLF cells were cultured in DMEM with 10% FBS at 37°C in a 5% CO_2_ (v/v) humidified incubator.

For lentivirus transduction, a 6-well plate of 50% confluent HEK293FT cells were transfected in OptiMEM with 5 μL Lipofectamine 2000 reagents (Invitrogen, Thermo Fisher Scientific), 2 μg packaging plasmids (equal volumes of pVSV-G, pMDL, and pRSV), and 2 μg lentivirus. Forty-eight hours later, the viral supernatant was harvested and filtered through a 0.45 μm filter (MilliporeSigma). For lentivirus transfection, cells were seeded into 6-well plates. The viral supernatant was added to culture media at an equal volume. After 24 hours, the culture media were supplemented with 2 μg/mL concentrations of puromycin for selection.

For the colony formation assay, cells transfected with pLenti-puro-sgCENPM/EGFP lentivirus were plated in 6-well culture plates at a density of 500 cells per well, in triplicate. Two weeks later, colonies were stained with Crystal Violet and then counted.

For knockdown studies, cells were transfected with scrambled siRNA or siRNA directed against the human *CENPM* gene (stB003419, RiboBio), according to the manufacturer’s recommendations. After incubation for 48 hours, cell proliferation was assessed using the EdU Cell Proliferation kit (Thermo Fisher Scientific). Experiments were repeated at least 3 times in triplicate.

### Analysis of chromosome segregation during mitosis.

CENPM-KO (sg*CENPM*) and control (sg*EGFP*) HLF cells were used. To synchronize the cells in the G_0_/G_1_ phase, cells were incubated in DMEM/F12 growth media containing 0.1% FBS for 24 hours, ensuring a maximum number of cells in the G_0_/G_1_ phase (35). The cells were then incubated in DMEM with 10% FBS for 24 hours at 37°C and 5% CO_2_. After incubation, cells were washed with PBS, fixed with ice-cold methanol, permeabilized with 0.1% Triton X-100, and blocked with 0.1% Triton X-100 and 10% normal goat serum in PBS. The cells were then incubated overnight at 4°C with antibodies against acetylated α-tubulin (1:200,MilliporeSigma) and γ-tubulin (1:500, MilliporeSigma), followed by a 1-hour incubation with fluorescent secondary antibodies (1:200). Nuclei were stained with DAPI (Prolong Gold with DAPI, Invitrogen, Thermo Fisher Scientific), and chromosomal lagging or mis-segregation was analyzed using confocal microscopy.

### Histology and IHC.

Mouse liver tissues were fixed in 4% paraformaldehyde overnight, embedded in paraffin, and sectioned as described previously (15, 36). For IHC, the sections were incubated with the primary antibodies overnight at 4°C. Immunoreactivity was visualized with the Vectastain ABC Elite Kit (Vector Laboratories) and DAB (Vector Laboratories). Slides were then counterstained with hematoxylin. The primary antibodies used are listed in Supplemental Table 4. Quantification was performed using ImageJ 1.8.0 software (NIH).

### Protein extraction and Western blot analysis.

Frozen mouse liver tumors were homogenized, and cultured cell samples were lysed in Mammalian Protein Extraction Reagent (Thermo Fisher Scientific) containing the Complete Protease Inhibitor Cocktail (Thermo Fisher Scientific). Protein concentrations were determined with the Bio-Rad Protein Assay Kit (Bio-Rad). The lysates were denatured by boiling in 2× Laemmli sample buffer (1610737, Bio-Rad). Aliquots of 30 μg protein lysates were separated by SDS-PAGE (M00654, GenScript) and then transferred onto PVDF membranes (Bio-Rad). Membranes were blocked in 10% nonfat milk in Tris-buffered saline containing 0.05% Tween-20 and incubated with primary antibodies at 4°C overnight. Then membranes were incubated with a HRP secondary antibody (Jackson ImmunoResearch Laboratories) for 1 hour at room temperature and developed with Clarity Western ECL Substrate (170-5061, Bio-Rad). The primary antibodies used are listed in Supplemental Table 5.

### RNA extraction, reverse transcription, and real-time PCR.

Total RNA was extracted from frozen mouse liver specimens and cultured cell samples using the Quick-RNA Miniprep Kit (R1055, Zymo Research). cDNA was generated using iScript Reverse Transcription Supermix (1708841, Bio-Rad Laboratories), according to the manufacturer’s instructions. mRNA expression was determined by quantitative PCR (qPCR) using iTaq Universal SYBR Green Supermix (1725124, Bio-Rad Laboratories) in the QuantStudio 6 Flex system (Applied Biosystems). The expression of each specific gene mRNA was normalized with the 18S rRNA. Thermal cycling conditions included an initial hold period at 95°C for 10 minutes, which was followed by a 3-step PCR program of 95°C for 15 seconds, 60°C for 1 minute, and 72°C for 30 seconds for a total of 40 cycles. The primers used in this study are listed in Supplemental Table 6.

### RNA-Seq analysis.

To analyze the role of FOXOs during c-MYC–induced hepatocarcinogenesis, total RNA was extracted from mice injected with c-MYC/pT3 (*n* = 3) or c-MYC/FOXO1AAA (*n* = 3), as well as from *FVB/N* WT normal livers (*n* = 4) using the Quick-RNA Miniprep Kit (Zymo Research, CA,USA). To analyze downstream target genes of p70S6K/RPS6 and 4EBP1/eIF4E, total RNA was extracted from mouse *c-MYC/MCL1/Rictor^KO^* *Tsc2^KO^* HCCs treated with MLN0128 (*n* = 3), everolimus (*n* = 3), or vehicle (*n* = 3), as well as from C57BL/6 WT normal livers (*n* = 3) using the Quick-RNA Miniprep Kit (Zymo Research). The experimental design included 4 groups: MLN0128 (MLN), everolimus (EVE), vehicle (VEH), and normal liver (NL). RNA quality control was determined using the Agilent RNA 6000 Nano Kit (Agilent Technologies) and Bioanalyzer (Agilent Technologies). Novogene performed library preparation and sequencing. All analyses were performed in R. Gene read counts were conducted in Ensembl Gene ID and converted to Entrez Gene ID. Corresponding symbol annotations and full gene names were added using the org.Mm.eg.db library. The R package edgeR and glmTreat function were used to identify differentially expressed genes (DEGs). DEGs were limited by a *P* value of 0.05 and an FDR of 0.05. The Venn diagram was drawn using the VennDiagram package. KEGG pathway enrichment analysis was performed using the ggplot2 package.

### ChIP assay.

SNU449 cells were transfected with pCMV4a–Flag–c-Myc using Lipofectamine 3000 (L3000001, Invitrogen, Thermo Fisher Scientific), and CHIP was performed using the Zymo-Spin ChIP Kit following the manufacturer’s instructions (catalog D5209, Zymo Research). Briefly, 15 μL Lipofectamine 3000 was diluted in 250 μL Opti-MEM and incubated at room temperature for 5 minutes. Separately, 10 μg plasmids and 20 μL P3000 were mixed in 250 μL Opti-MEM. After 5 minutes, the 2 mixtures were combined and incubated for an additional 20 minutes at room temperature. This DNA-lipid complex and 2.5 × 10^6^ cells were then added to the cell culture plate. After 48 hours of transfection, the cells were harvested. DNA and protein in the cell samples were crosslinked with 1% formaldehyde, followed by sonication (30 seconds “ON,” 30 seconds “OFF” for 12 cycles) on ice. Immunoprecipitation was performed using a Flag-tag antibody (Proteintech, 66008-4-Ig) overnight at 4°C, with rabbit anti-IgG (no. 2729, 1:1,000; Cell Signaling Technology) as a negative control. DNA-protein complexes were pulled down using protein A magnetic beads and reverse-crosslinked to release the DNA. The purified DNA was used as a template for PCR. Input samples served as positive controls. The PCR cycling conditions were as follows: 10 minutes at 98°C, followed by 35 cycles of 10 seconds at 98°C, 5 seconds at 55°C, and 20 seconds at 72°C. PCR products were then run on a DNA gel to visualize the target bands. The primers specific for the *CENPM* promoter region are: *CENPM*-ChIP forward, 5′-GAATGAAAGTGAACAAAGGAAT-3′; *CENPM*-ChIP reverse, 5′-CCTCTTAAAGGAACCGAACC-3′.

### CUT&RUN assay.

The CUT&RUN assay was conducted following the manufacturer’s protocol (HD101, Vazyme). Briefly, SNU449 cells were rinsed with PBS and incubated with ConA Beads Pro at room temperature for 10 minutes. c-Myc/N-Myc antibody (13987S, 1:50; Cell Signaling Technology) was added and incubated at 4°C overnight. IgG (2729S, 1:50; Cell Signaling Technology) was used as a negative control. The samples were then washed twice, followed by the addition of pG-MNase enzyme and incubation at 4°C for 1 hour. After washing twice, CaCl_2_ was added, and the samples were incubated for 1.5 hours on ice. Stop buffer was then added, and the samples were incubated at 37°C for 30 minutes. Finally, DNA was extracted and quantified by qPCR. The primers specific for *CENPM* promoter region were: *CENPM*-ChIP forward, 5′-GAATGAAAGTGAACAAAGGAAT-3′; *CENPM*-ChIP reverse, 5′-CTTCTTAAAGGAACCGAACC-3′.

### Dual-luciferase reporter assay.

The pCMV4a–Flag–c-Myc–transfected SNU449 human HCC cells were plated in triplicate in 24-well plates at 70%–80% confluence. Plasmids were transfected using the Lipofectamine 2000 reagents (Invitrogen, Thermo Fisher Scientific). In brief, cells were transfected with 600 ng pLG3-*CENPM* promoter plasmids or pGL3-Motif-Mut plasmids. The pGL3 empty vector plasmid was applied as a control. Meanwhile, HCC cells in each group were also transfected with 16 ng pRL-CMV plasmids. Cells were harvested 48 hours after transfection. According to the manufacturer’s protocol, we assessed the luciferase activity using the Dual-Luciferase Reporter Assay System (catalog 1910, Promega). The Synergy HT microplate read firefly and *Renilla* luciferase. Normalization to *Renilla* luciferase was performed in all samples. Experiments were repeated at least 3 times in triplicate.

### Retrieval and analysis of TCGA human HCC data.

To investigate the relationship between MYC activation and *TSC* mutation status in human HCC samples, TGCA datasets were retrieved from the cBioPortal for Cancer Genomics (http://www.cbioportal.org). The overall sample size was 374 HCCs from TCGA-LIHC database. The mutation data were extracted from the cBioPortal for Cancer Genomics. The data were analyzed and visualized in R using multiple packages. For the MYC activation status, we extracted expression data on 30 well-characterized c-MYC target genes from TCGA-LIHC database. In order to reflect the c-MYC activation trends, all 30 of the c-MYC target genes expression levels were used for clustering analysis of transcription abundance. All genes were clustered into 3 expression profiles (MYC-high, MYC-low and MYC-medium) using the K-means clustering method. The data objects with similar characteristics of c-MYC activation were grouped into the same clusters. Heatmaps were generated using the pheatmap package in R.

### Statistics.

The GraphPad Prism 7.0 (GraphPad Software) was used to analyze the data. The data are presented as the mean ± SD. Statistical analyses were conducted using a 2-tailed Student’s *t* test, χ^2^ test, and 1-way ANOVA. Survival curves were estimated using the Kaplan-Meier method and compared using the log-rank test. A *P* value of less than 0.05 was considered statistically significant.

### Study approval.

All mouse experiments were performed in accordance with protocols approved by the IACUCs at UCSF (protocol no. AN185770) and the University of Hawaii Cancer Center.

### Data availability.

The RNA-Seq data for this study were deposited in the Gene Expression Omnibus (GEO) database (GSE275889, GSE276215). All datasets generated and analyzed for the current study are available from the corresponding author upon reasonable request, and values underlying the data presented in each graph and as means are provided in the Supporting Data Value files.

## Author contributions

SL, HW, and XC conceived of and designed the study. YZ, SZ, GQ, XW, AY, JW, ZX, ME, and GC performed the experiments. YZ, SZ, and HW drafted the manuscript. JC, NC, and MX provided technical and material support. XW, HX, and SD performed data analysis and interpretation of the sequencing data. SL, DFC, XC, XW, YD, XS, AY, and HW reviewed and revised the manuscript. All authors read and approved the final version of the manuscript.

## Supplementary Material

Supplemental data

Unedited blot and gel images

Supplemental table 1

Supporting data values

## Version 1 09/26/2024

In-Press Preview

## Version 2 11/15/2024

Electronic publication
