# Supplementary material for: TSC/mTORC1 mediates mTORC2/AKT1 signaling in c-MYC–induced murine hepatocarcinogenesis via centromere protein M
Source: J Clin Invest. 2024 Sep 26;134(22):e174415. doi: 10.1172/JCI174415 (PMC11563669; doi:10.1172/JCI174415)
Supplement: Supplemental data [file jci-134-174415-s205.pdf]

TSC/mTORC1 mediates mTORC2/AKT1 signaling in c-MYC-induced murine hepatocarcinogenesis via centromere protein M

Yi Zhou[1, 2*], Shu Zhang[3,4*], Guoteng Qiu[5,6], Xue Wang[7], Andrew Yonemura[7], Hongwei Xu[2,5], Guofei Cui[2,7], Shanshan Deng[2,7], Joanne Chun[7], Nianyong Chen[3,4,8], Meng Xu[9], Xinhua Song[10], Jingwen Wang[10], Zijing Xu[10], Youping Deng[7,11], Matthias Evert[12], Diego F. Calvisi[12], Shumei Lin[1], Haichuan Wang[5,6] and Xin Chen[2,7]

Supplementary Fig. 1

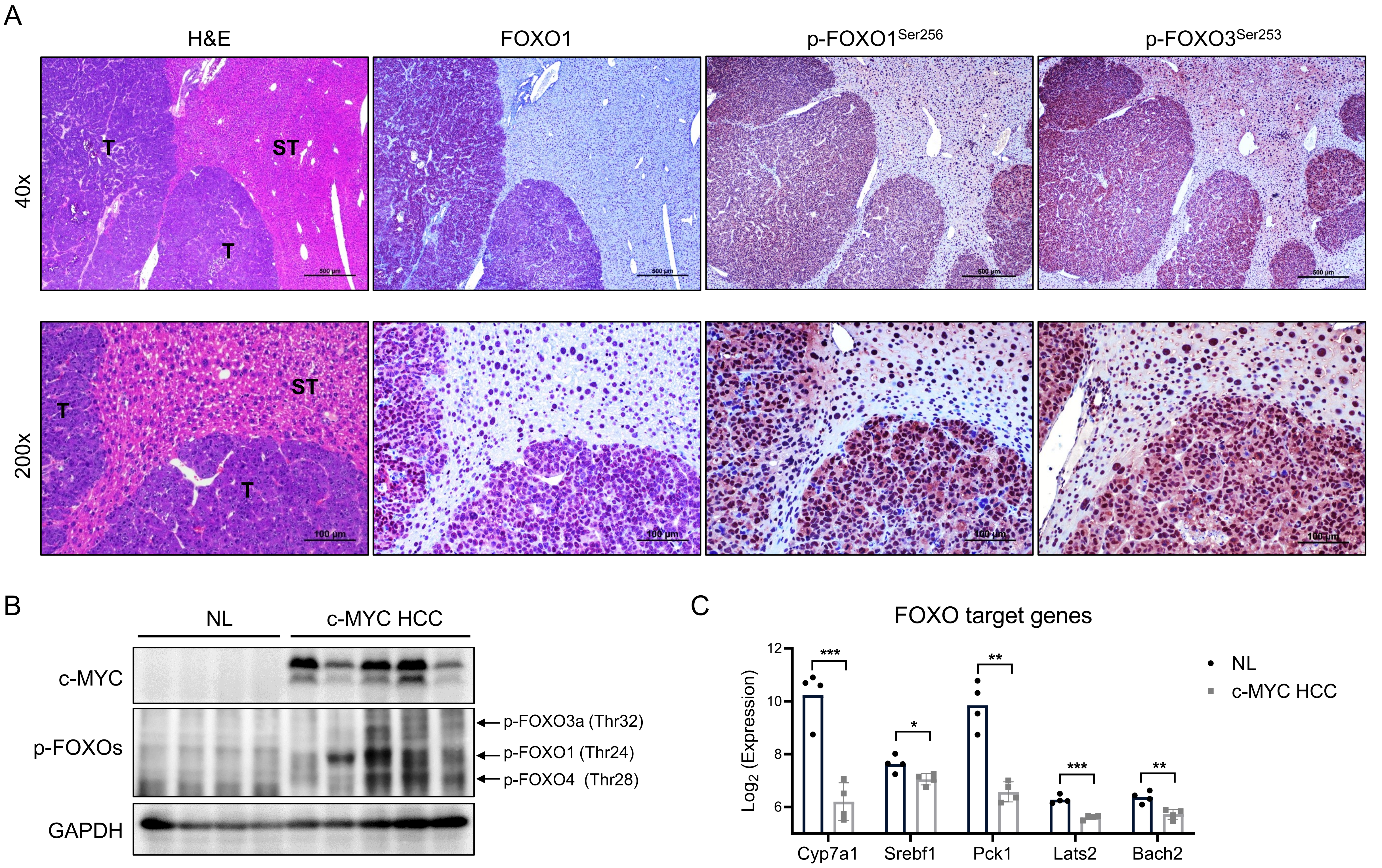

Supplementary Fig. 1. Activation status of FOXO1 in murine c-MYC HCC. (A) Representative images: H&E staining, and immunohistochemical staining of FOXO1, p-FOXO1^{Ser256}, and p-FOXO3^{Ser253}. Scale bars: 500 μm for 40× images and 100 μm for 200× images. (B) Western blot results show the expression of c-MYC and p-FOXOs in normal livers (NL) and c-Myc HCCs. GAPDH was used as the loading control. (C) Microarray analysis of FOXO1 downstream genes in normal livers (NL) and c-Myc HCCs (n = 4, 4). Student's t-test. *, *p*<0.05. **, *p*<0.01. ***, *p*<0.001.

Supplementary Fig. 2

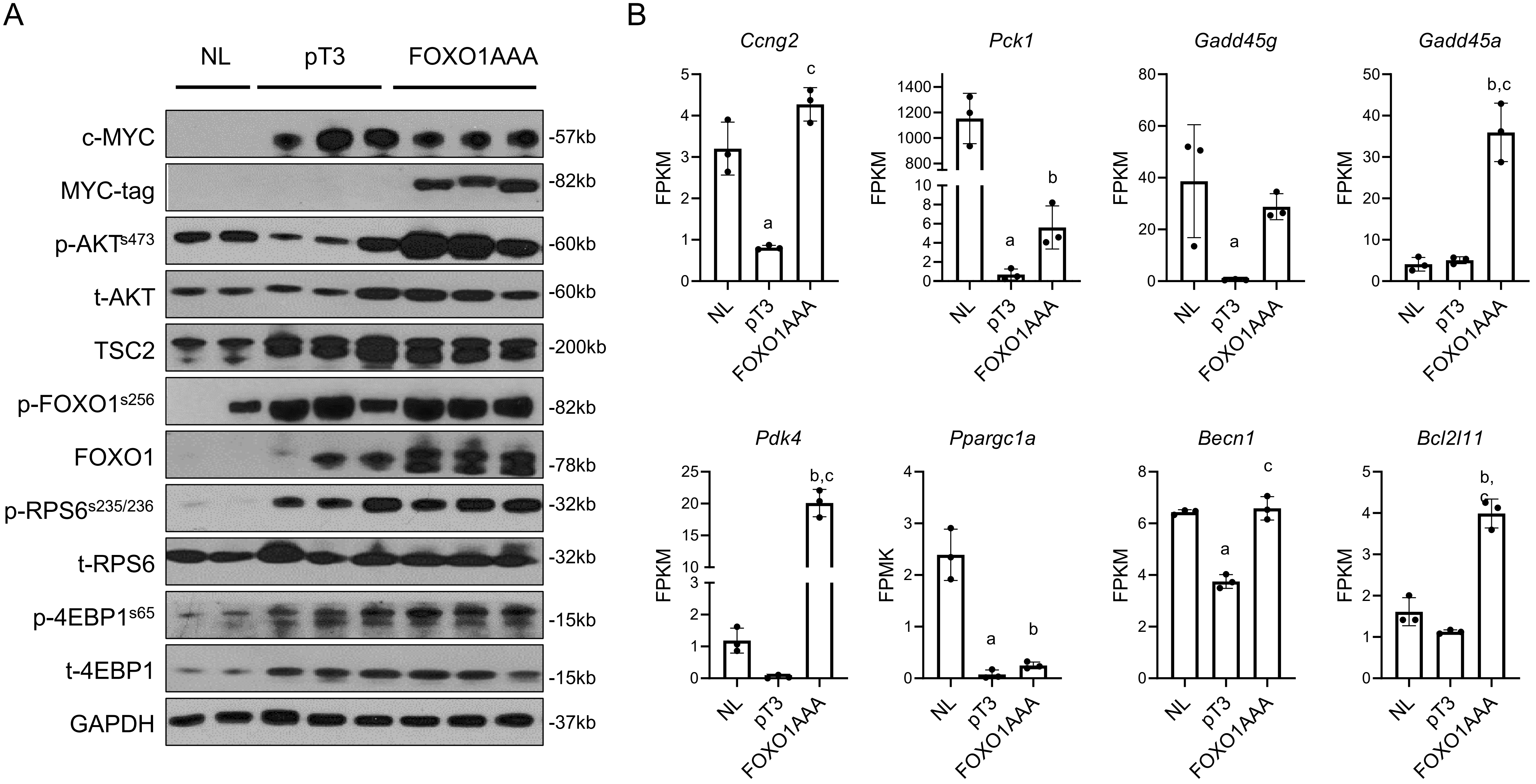

Supplementary Fig. 2. Effects of FOXO1 activation on c-MYC liver tumors. **(A)** Western blot results show the levels of c-MYC, MYC-tagged FOXO1AAA, and key molecules downstream of mTORC2 signaling. GAPDH was used as the loading control. **(B)** RNAseq results of FOXO1 target genes in the c-MYC/pT3 and c-MYC/FOXO1AAA HCCs (n = 3, 3). Data are presented as mean ± SD. Tukey–Kramer test. At least *p*<0.05. a, versus NL; b, versus pT3; c, versus NL. Abbreviations: NL, normal liver. pT3, pT3-EF1α.

Supplementary Fig. 3

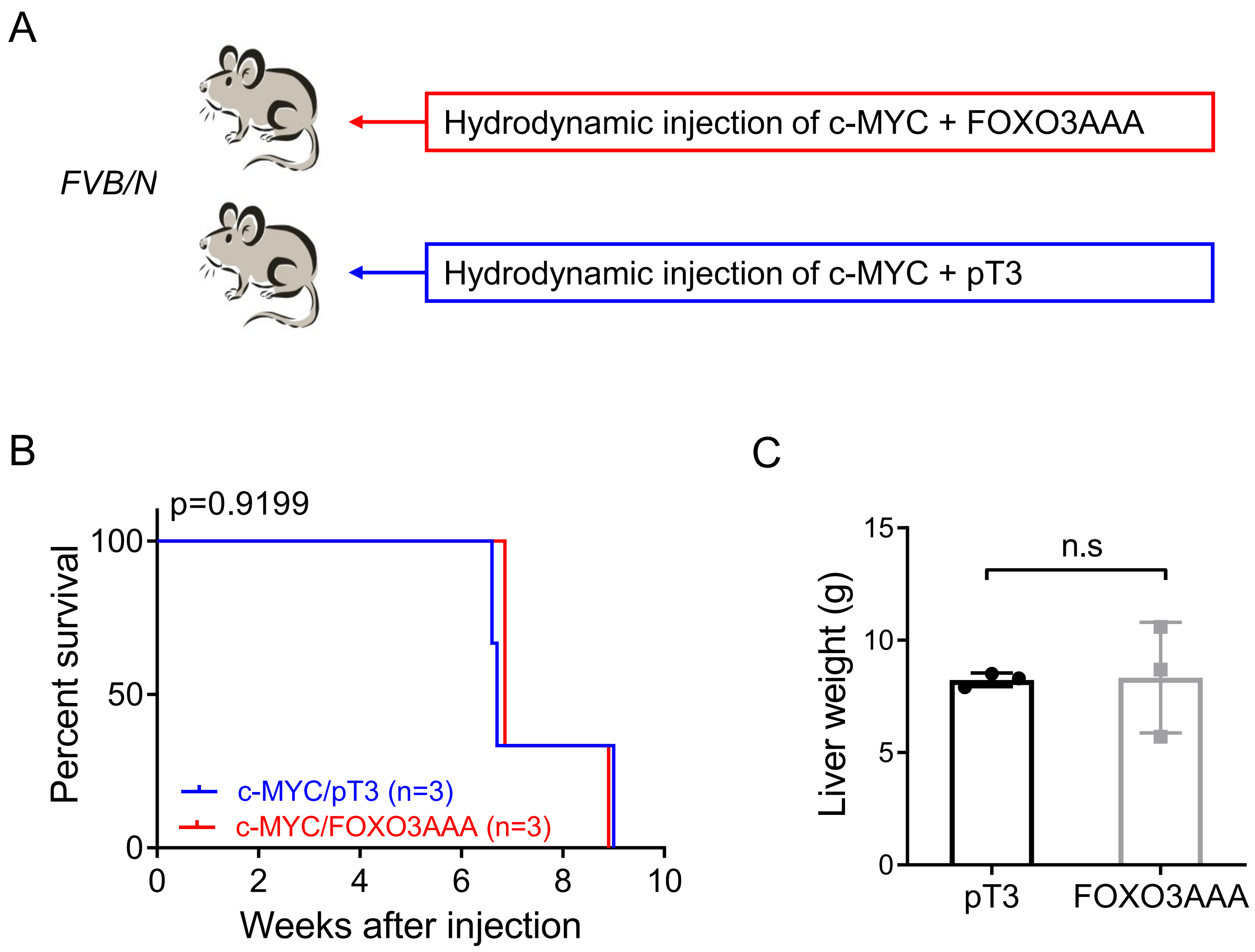

Supplementary Fig. 3. Effect of FOXO3 activation on c-MYC-induced hepatocarcinogenesis. (A) Study design. FVB/N mice were hydrodynamically injected with plasmid mixtures of c-MYC and a constitutively active mutant form of FOXO3 (FOXO3AAA) in pT3-EF1α backbone (c-MYC/FOXO3AAA, n = 3). The control mice were hydrodynamically injected with c-MYC and pT3-EF1α empty vector (c-MYC/pT3, n = 3). Mice were monitored for tumor development and were euthanized when moribund tumors developed or till the end of the observation period. **(B)** Survival curve of mice in two groups. The Kaplan-Meier comparison was performed, p = 0.9199. **(C)** Comparison of liver weight between the two groups. Data are presented as mean ± SD. Student's t-test. ns, no significant. Abbreviations: pT3, pT3-EF1α.

Supplementary Fig. 4

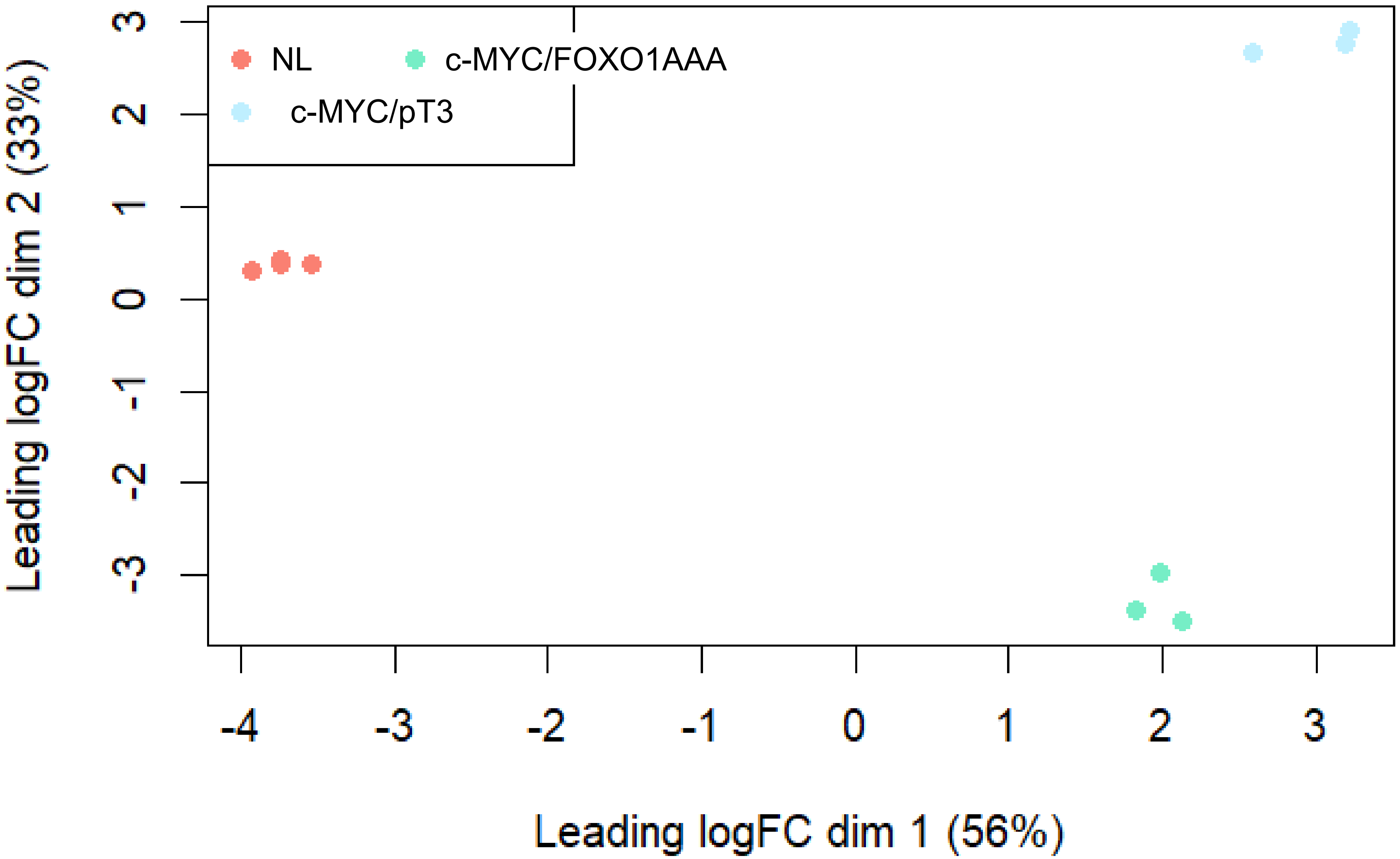

Supplementary Fig. 4. Principal component analysis (PCA) of RNA-seq data for normal liver (NL), c-MYC/pT3, and c-MYC/FoxO1AAA in mouse model (n = 3, 3, 3).

Supplementary Fig. 5

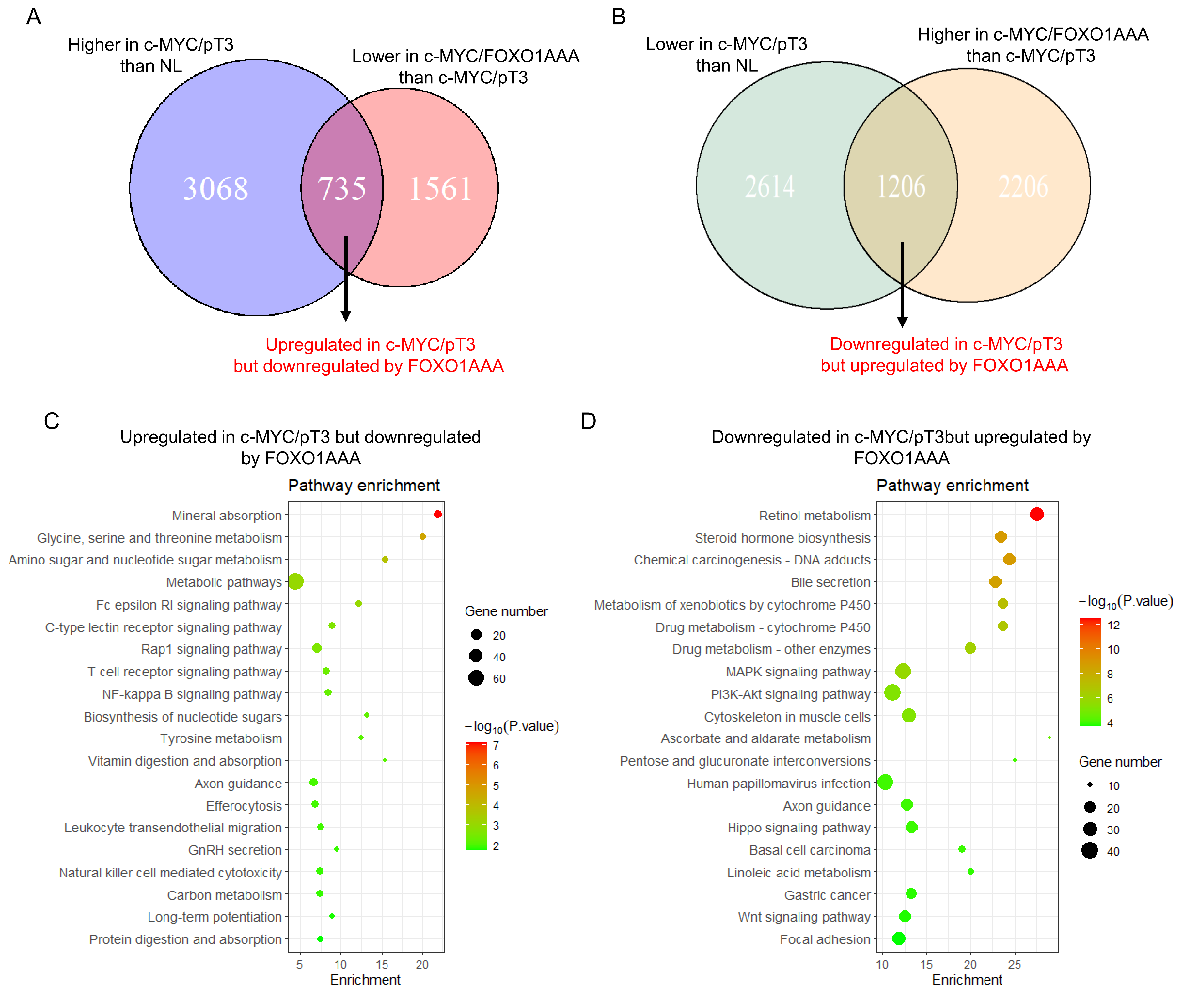

Supplementary Fig. 5. Analysis of RNA-seq data for NL, c-MYC/pT3, and c-MYC/FoxO1AAA. (A) Venn diagram displaying the genes that are upregulated in c-MYC/pT3 but downregulated by FOXO1AAA. **(B)** Venn diagram displaying the genes downregulated in c-MYC/pT3 but upregulated by FOXO1AAA. **(C)** KEGG analysis of the genes upregulated in c-MYC/pT3 but downregulated by FOXO1AAA. **(D)** KEGG analysis of the genes downregulated in c-MYC/pT3 but upregulated by FOXO1AAA. Abbreviations: NL, normal liver. pT3, pT3-EF1α.

Supplementary Fig. 6

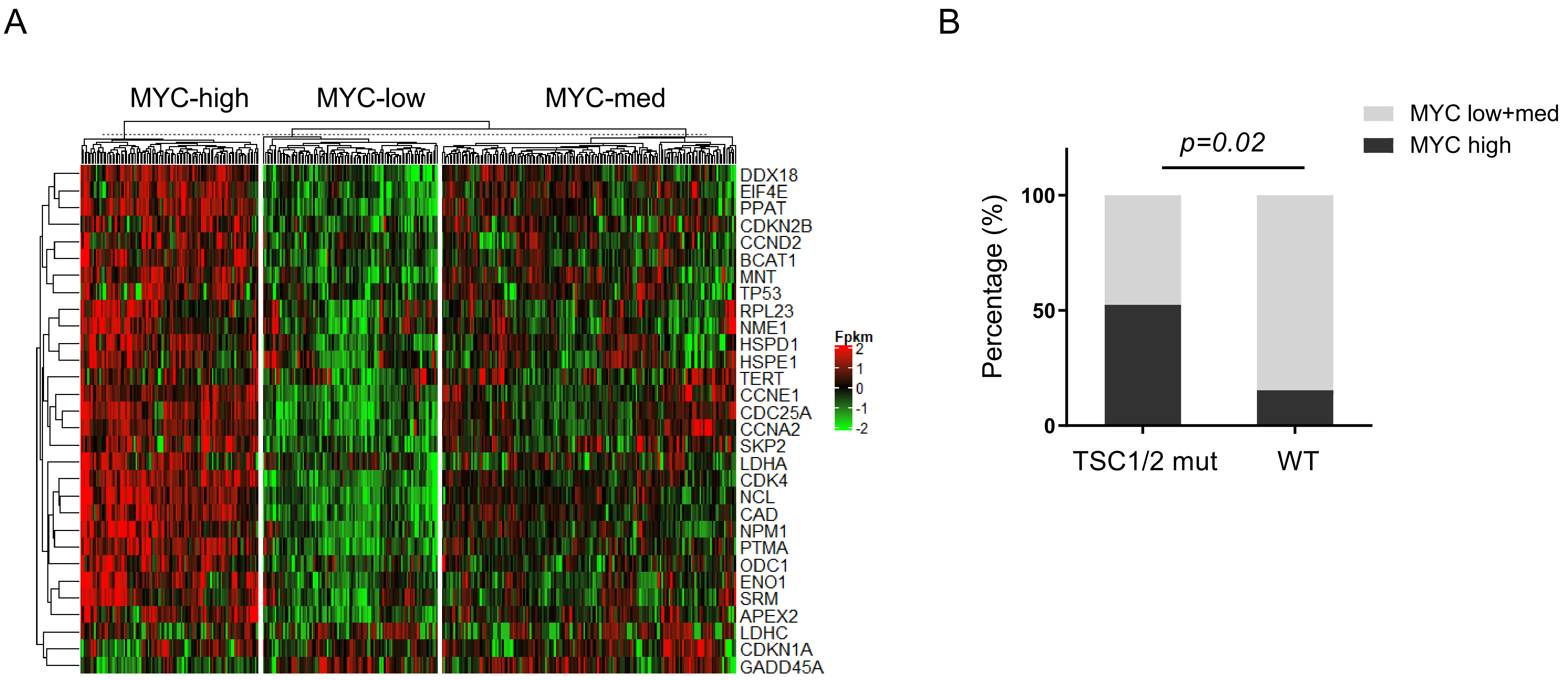

Supplementary Fig. 6. TSC mutations correlate with c-MYC activation status in human HCCs. (A) Heatmap displaying expression levels of c-MYC signature genes among the HCC samples from the TCGA-LIHC dataset. The samples were grouped into three categories based on their c-MYC activation status: high (MYC-high), low (MYC-low), and medium (MYC-med). **(B)** Comparison of the percentages of HCC samples with high c-MYC activation status between the TSC1/2 mutated HCCs and the TSC1/2 wild-type HCCs. Data were analyzed using Chi-square test. Abbreviations: WT, wild-type.

Supplementary Fig. 7

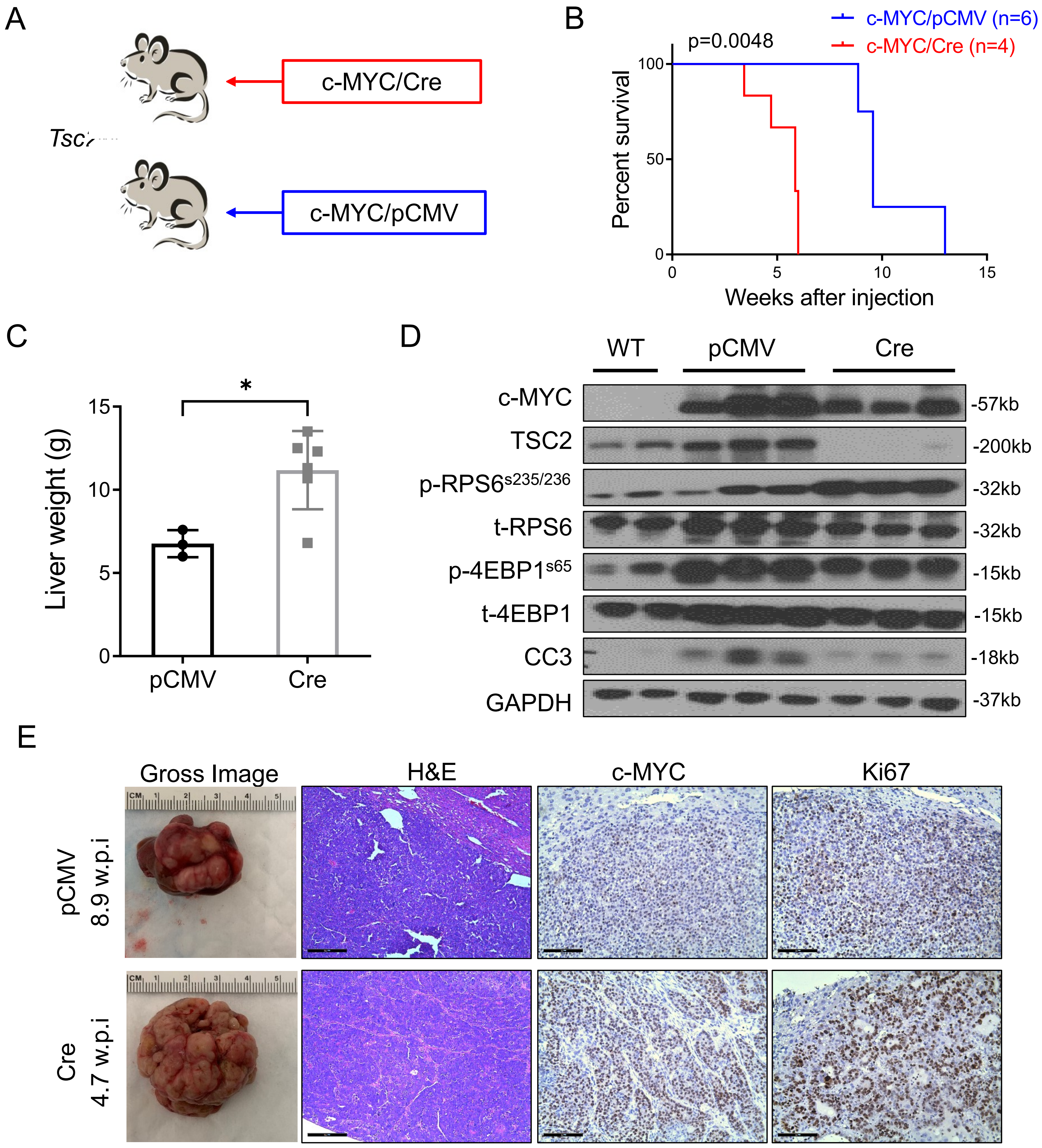

Supplementary Fig. 7. Deletion of *Tsc2* accelerates c-Myc-induced hepatocarcinogenesis. (A) Study design. *TSC2*$^{fl/fl}$ conditional knockout mice in *FVB/N* genetic background were hydrodynamically injected with plasmid mixtures of c-MYC and Cre recombinase in the pCMV backbone (c-MYC/Cre, n = 4). Control mice were hydrodynamically injected with c-MYC and pCMV empty vector (c-MYC/pCMV, n = 6). Mice were monitored for tumor development and euthanized either upon the development of large tumors or at the end of the observation period. **(B)** Survival curve of mice in both groups. The Kaplan-Meier comparison was performed, p = 0.0006. **(C)** Comparison of liver weight between the two groups. Data are presented as mean ± SD. Student's t-test. *, $p<0.05$. **(D)** Western blot results showing expressions of c-MYC, TSC2, cleaved caspase 3 (CC3), and key molecules in the p70S6K/RPS6 and 4EBP1/eIF4E cascades. GAPDH was used as the loading control. **(E)** Representative images: gross views of the liver, H&E staining, and immunohistochemical stainings of c-MYC and Ki67. Scale bars: 500μm for H&E, 100μm for c-MYC and Ki67.

Supplementary Fig. 8

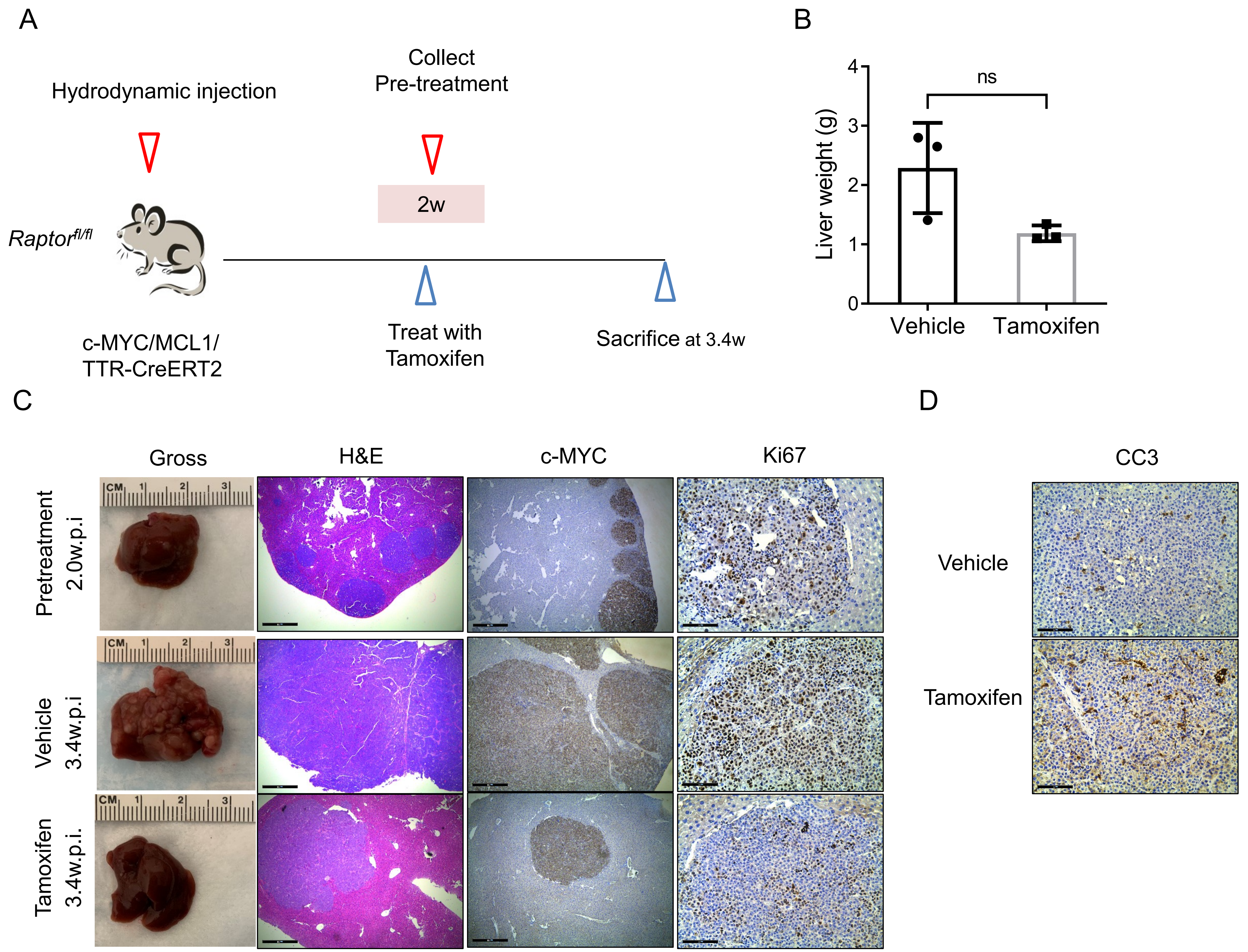

Supplementary Fig. 8. mTOCR1 impairment induces regression of c-MYC HCC. Tamoxifen-treated mice displayed tumor regression upon dissection, leading to a significantly lower liver weight, reduced proliferation, and increased apoptosis. **(A)** Study design. *Raptor*$^{fl/fl}$ conditional knockout mice were hydrodynamically injected with plasmid mixtures of c-MYC/MCL1 and tamoxifen-inducible CreERT2 recombinase under the liver-specific TTR promoter (c-Myc/MCL1/TTR-CreERT2). Two weeks post-injection (when tumor nodules were observed), one group of mice was sacrificed, and liver analysis was performed as the pre-treatment group. The remaining mice were treated with tamoxifen or vehicle for 1.4 weeks, followed by analysis after sacrifice. **(B)** Comparison of liver weight between the two groups (n = 3, 3). Data are presented as mean ± SD. Student's t-test. ns, no significant.**(C)** Representative images: gross views of the liver, H&E staining, and immunohistochemical staining of Ki67 and c-MYC. **(D)** Representative images of cleaved caspase 3 (CC3) staining in vehicle- and tamoxifen- treated mice. Scale bars: 500µm for H&E and c-MYC, 100µm for Ki67 and CC3.

Supplementary Fig. 9

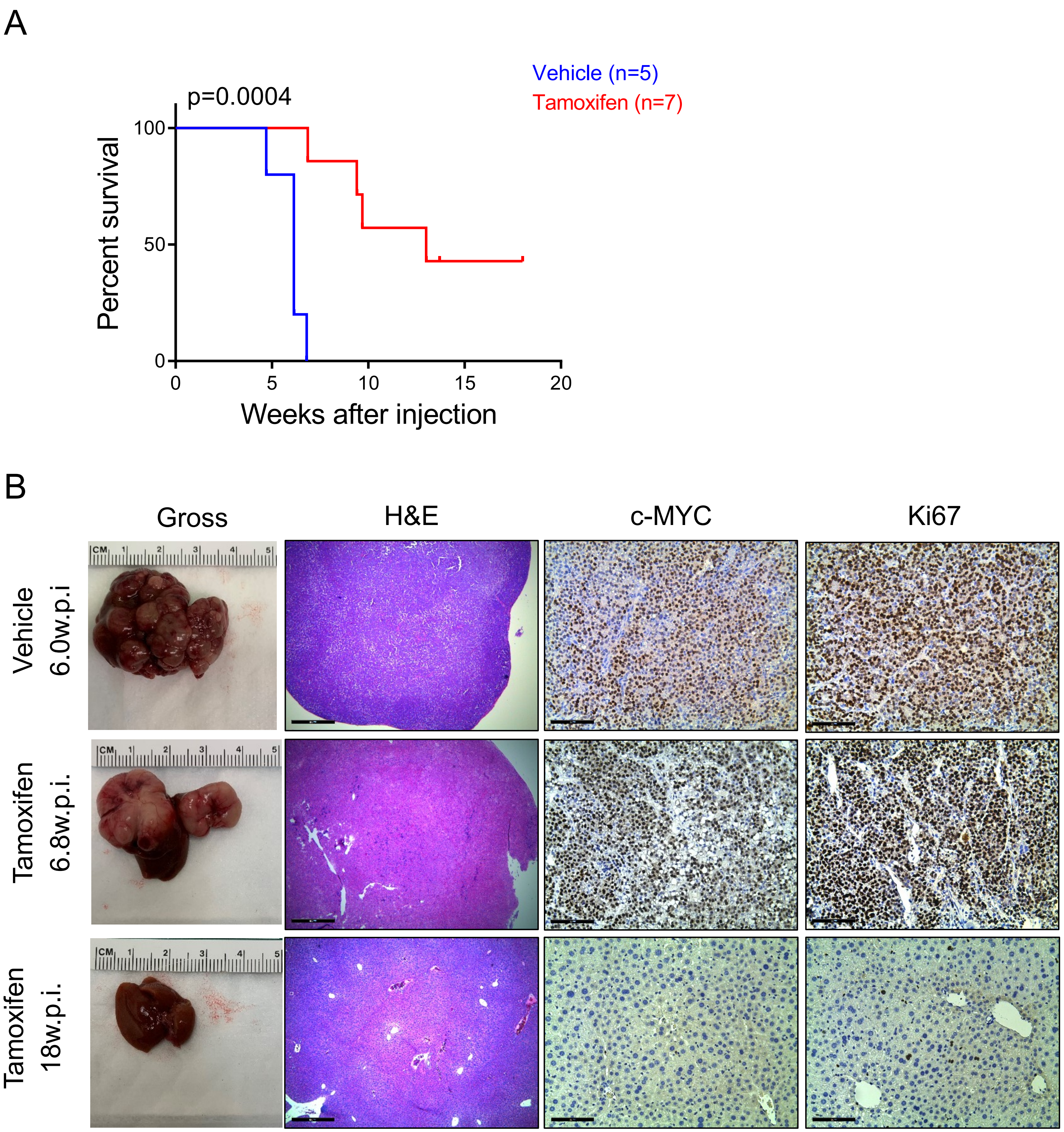

Supplementary Fig. 9. mTOCR1 impairment improves overall survival of c-MYC mice. (A) Survival curve of mice in both groups (n = 5, 7). The Kaplan-Meier comparison was performed, p = 0.0004. **(B)** Six of seven tamoxifen-treated mice developed a few, separate tumor nodules (ranging from 1 to 3) between 6.8 and 13.7 weeks post-injection. One mouse liver collected at 18 weeks appeared completely normal, whereas vehicle-treated mice exhibited a high tumor burden with numerous nodules between 4.7 and 8.9 weeks post-injection. Representative images: gross views of the liver, H&E staining, and immunohistochemical staining of c-MYC and Ki67. Scale bars: 500 μm for H&E, 100 μm for c-MYC, and Ki67.

Supplementary Fig. 10

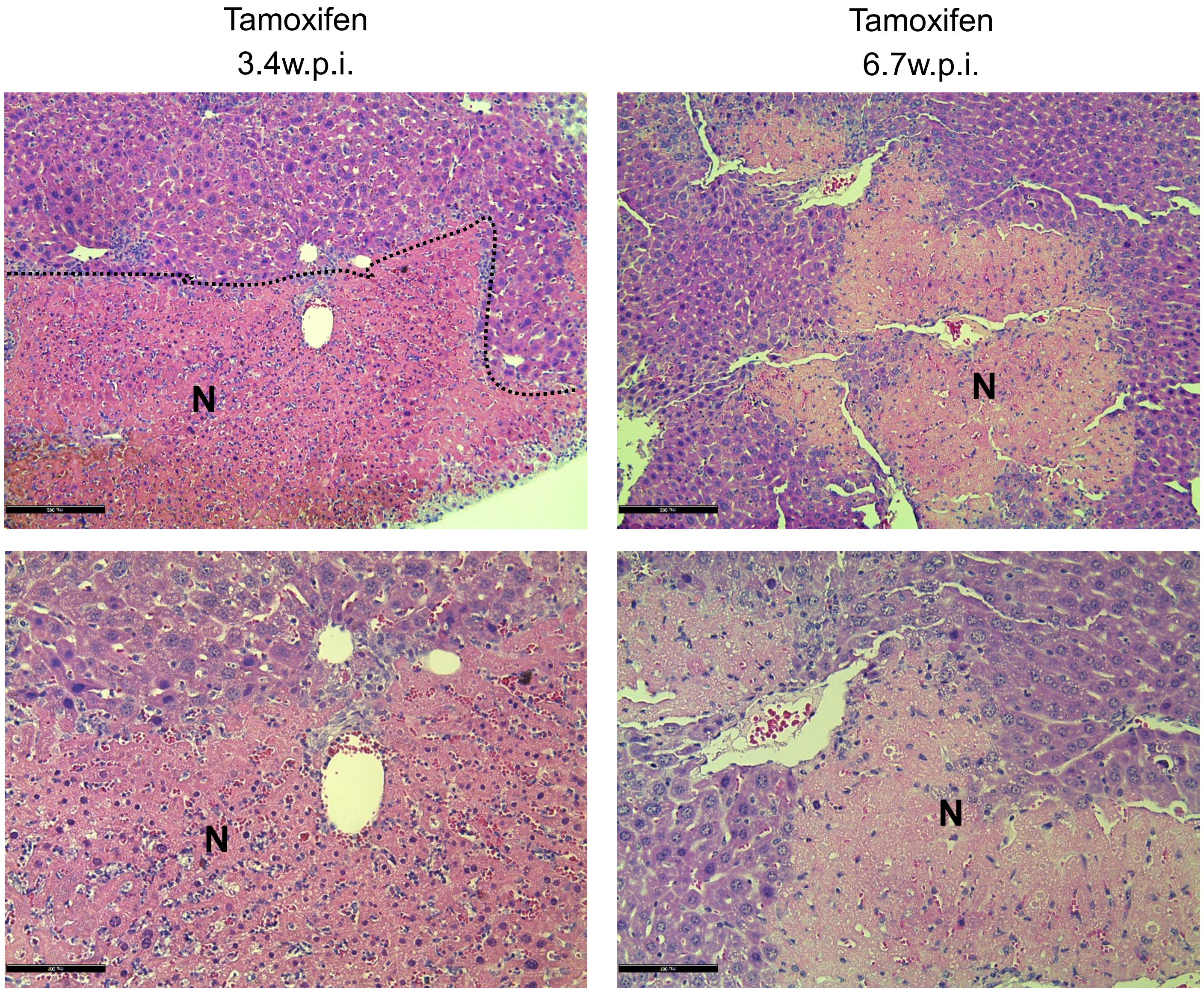

Supplementary Fig. 10. Representative images of H&E staining of c-MYC HCCs at different time points after tamoxifen treatment showed increased necrotic areas (N). Scale bars: 200μm.

Supplementary Fig. 11

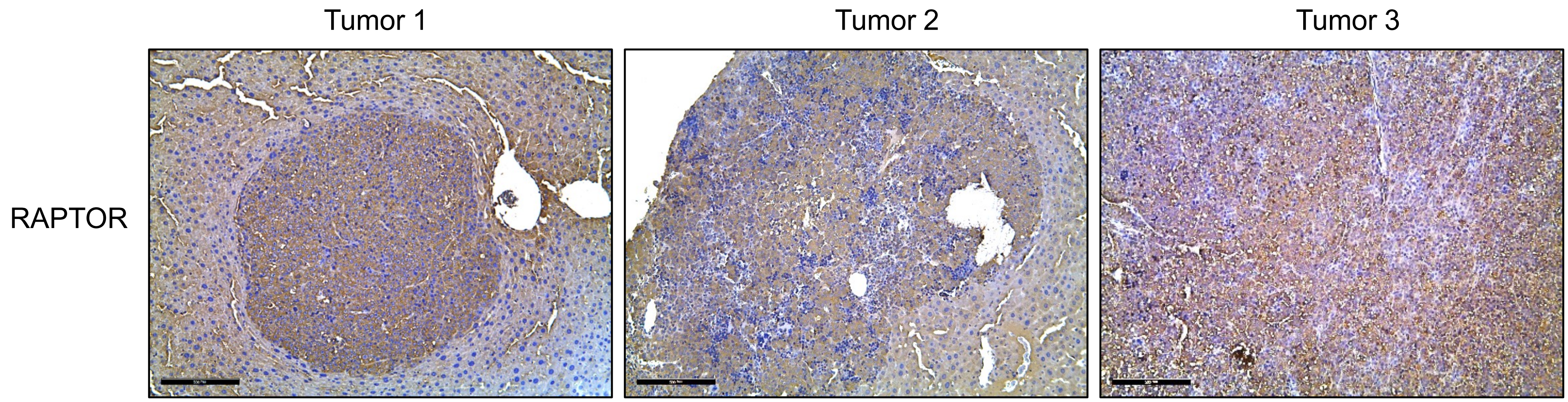

Supplementary Fig. 11. Raptor staining revealed failed Cre recombinase activity in the tumor area of the tamoxifen-treated group. Large areas of necrosis were frequently observed in tamoxifen-treated livers. Thus, we speculate that the observed failure of tumor regression was due to off-target effects of the tamoxifen-inducible Cre transgene system.

Supplementary Fig. 12

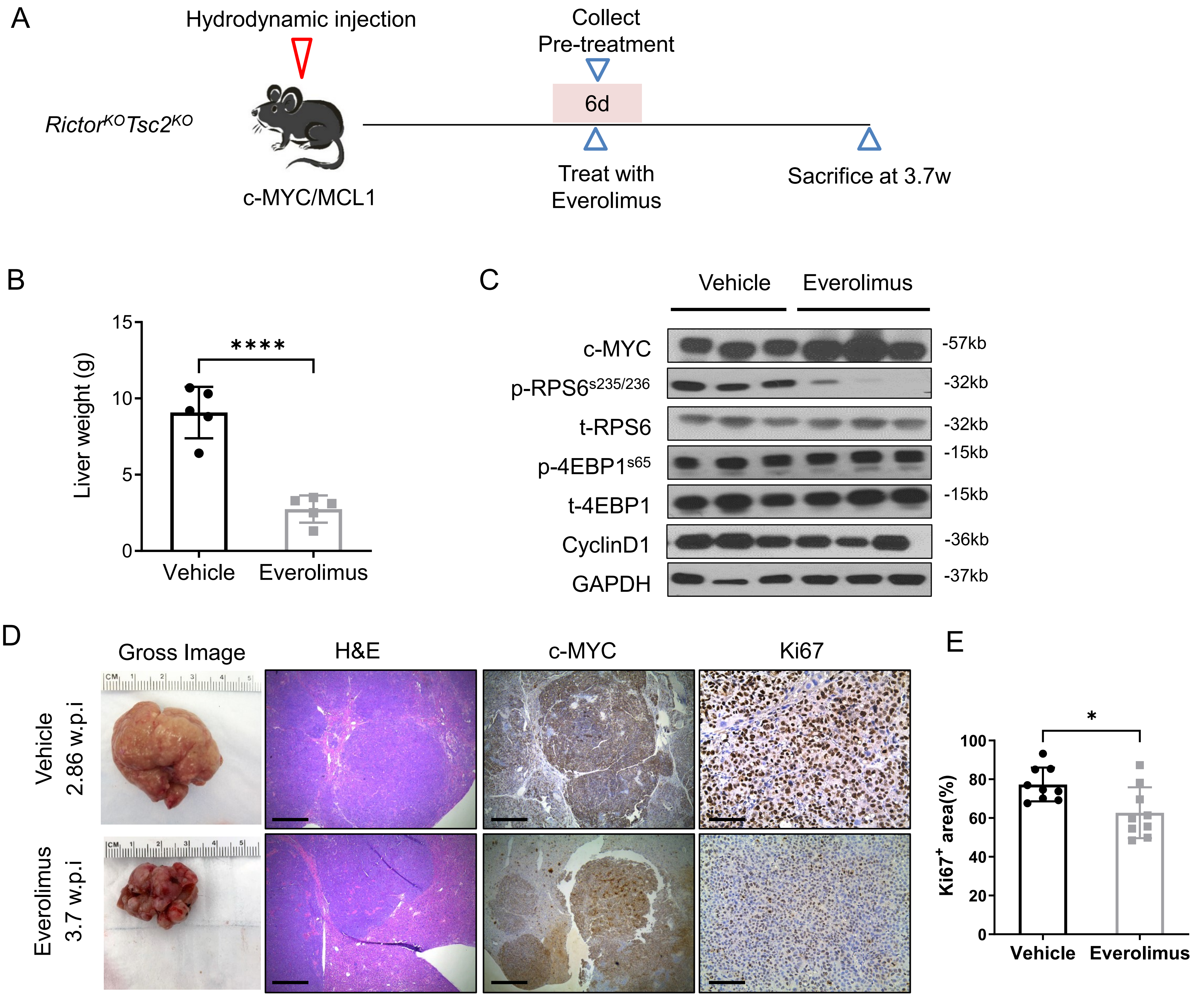

Supplementary Fig. 12. mTORC1 inhibitor demonstrates moderate antitumor activity in the c-Myc/MCL1/*Rictor*^{KO}*Tsc2*^{KO} HCCs. (A) Study design. c-MYC/MCL1/*Rictor*^{KO}*Tsc2*^{KO} murine tumor model was established through hydrodynamic injection. Six days post-injection, one group of mice (n = 3) was sacrificed, and liver analysis was performed as the pre-treatment group. The remaining mice were treated with the mTORC1 inhibitor everolimus (n = 5) or vehicle (n = 5) for 3 weeks, followed by analysis after sacrifice. **(B)** Comparison of liver weight between the everolimus and vehicle-treated groups. **(C)** Western blot results showing expressions of c-MYC, Cyclin D1, and key molecules in the p70S6K/RPS6 and 4EBP1/eIF4E cascades. GAPDH was used as the loading control. **(D)** Representative images: gross views of the liver (This figure shared a control group with MLN0128 in Figure 4E). H&E staining, and immunohistochemical staining of c-MYC and Ki67. Scale bars: 500μm for H&E and c-MYC, 100μm for Ki67. **(E)** Quantification results of the percentage of Ki67-positive cells in the two groups. Data are presented as mean ± SD. Student's t-test. *, p<0.05. ****, p<0.0001.

Supplementary Fig. 13

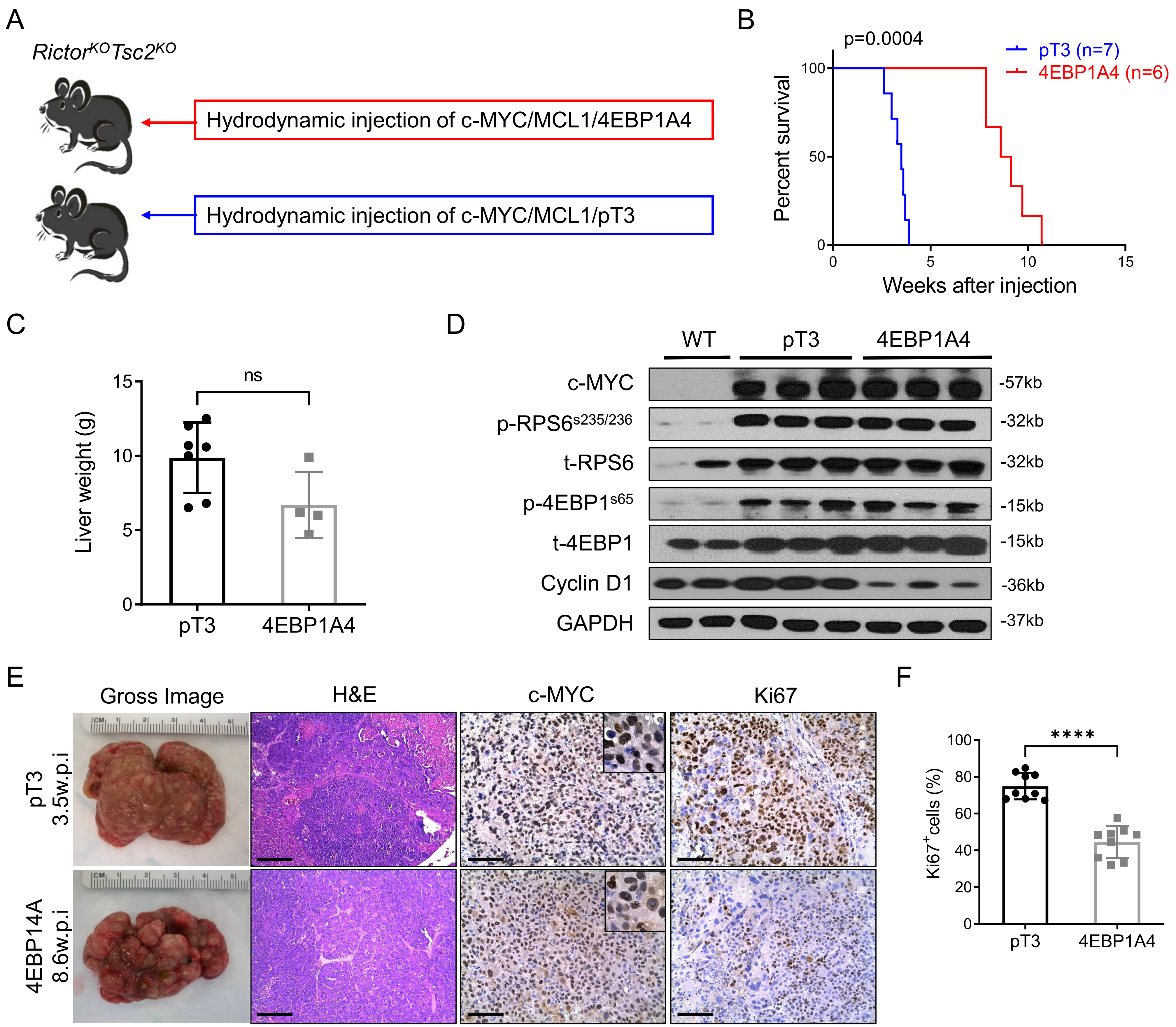

Supplementary Fig. 13. Inhibition of 4EBP1/eIF4E delays tumorigenesis of c-Myc/MCL1/*$Rictor^{KO}Tsc2^{KO}$* HCCs. **(A)** Study design. *$Rictor^{fl/fl}Tsc2^{fl/fl}$* mice were hydrodynamically injected with c-MYC/MCL1/Cre and the unphosphorylatable form of 4EBP1 (c-MYC/MCL1/4EBP1A4/*$Ricto^{KO}Tsc2^{KO}$*, n = 7). Control mice were injected with c-MYC/MCL1/Cre and pT3 empty vector (c-MYC/MCL1/pT3/*$Ricto^{KO}Tsc2^{KO}$*, n = 6). **(B)** Survival curve of mice in both groups. The Kaplan-Meier comparison was performed, p = 0.0006. **(C)** Comparison of liver weight between the two groups. **(D)** Western blot results show the levels of c-MYC, cyclin D1, and key molecules in the p70S6K/RPS6 and 4EBP1/eIF4E cascades. GAPDH was used as the loading control. **(E)** Representative images: gross views of the liver, H&E staining, and immunohistochemical staining of c-MYC and Ki67. Scale bars: 500μm for H&E, 100μm for c-MYC, and Ki67. **(F)** Quantification results of percentage of Ki67 positive cells in the two groups. Data are presented as mean ± SD. Student's t-test. ns, no significant. ****, p<0.0001.

Supplementary Fig. 14

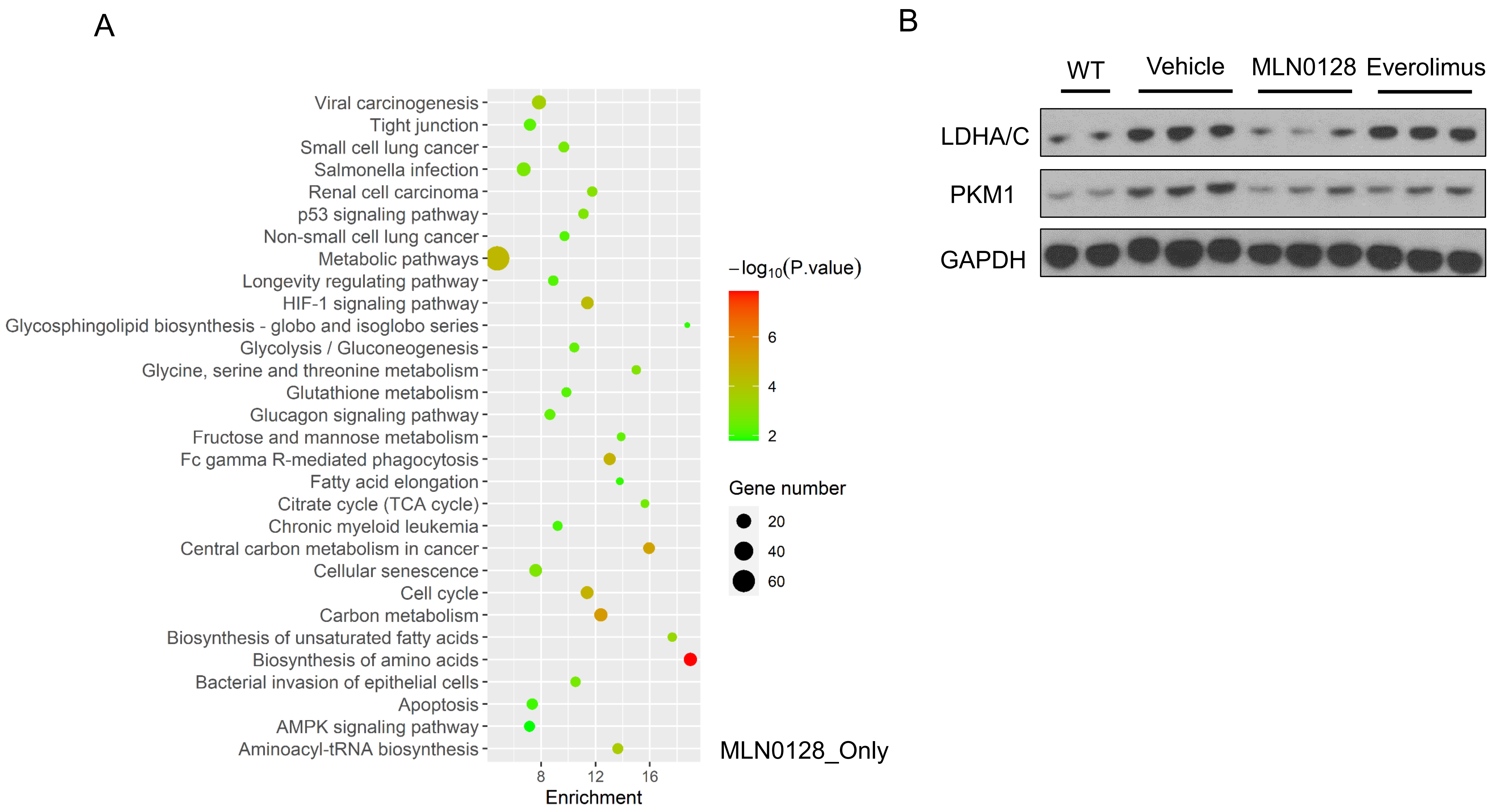

This figure is related to Figure 5D

Supplementary Fig. 14. Analysis of p70S6K/RPS6 and 4EBP1/eIF4E downstream target genes. (A) KEGG analysis of the differentially expressed genes downregulated by MLN0128, but not by everolimus. **(B)** Western blot results showing levels of LDHA/C and PKM2 in wild-type livers (WT), Vehicle-treated, MLN0128-treated, or Everolimus-treated c-MYC/MCL1/*Rictor*^{KO}*Tsc2*^{KO} HCCs. GAPDH was used as the loading control.

Supplementary Fig. 15

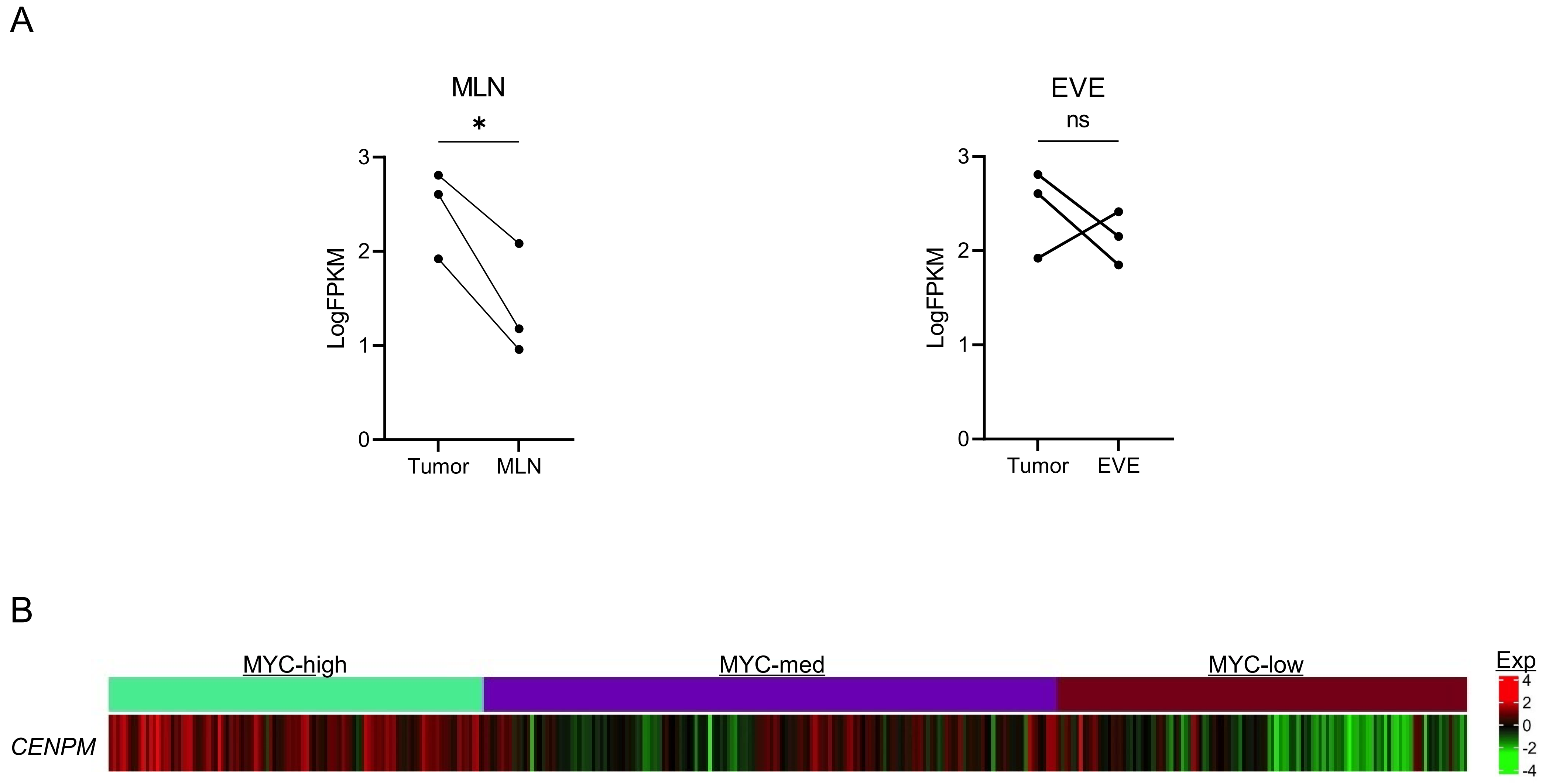

Supplementary Fig. 15. Analysis of the correlation between CENPM expression and MYC activation. **(A)** The CENPM expression in mouse c-MYC HCC upon MLN0128 (MLN) or Everolimus (EVE) treatment (n = 3, 3). Data are presented as mean ± SD. paired t test. ns, no significant. *, $p<0.05$. **(B)** The correlation between CENPM expression and the MYC activation levels based on the TCGA-LIHC database.

Supplementary Fig. 16

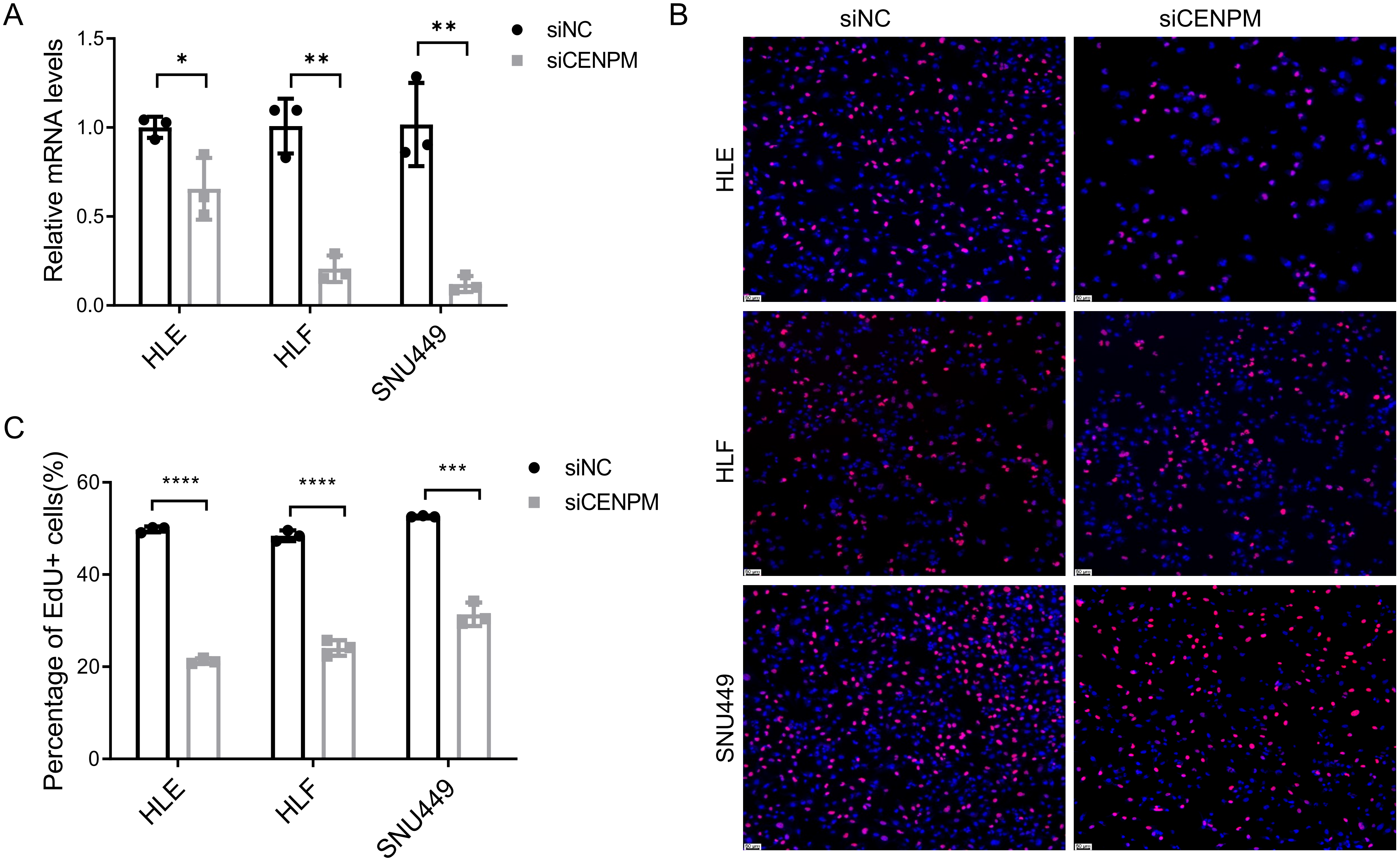

Supplementary Fig. 16. Silencing of CENPM inhibits human HCC cell proliferation. (A) qPCR results showing *CENPM* mRNA expression in siCENPM and control siRNA (siNC) transfected human HCC cells. **(B, C)** Representative images (B) and quantification (C) of EdU staining in the siNC, or siCENPM transfected human HCC cells. Data are presented as mean ± SD. Student's t-test. *, p<0.05, **, p<0.01, ***, p<0.001, ****, p<0.0001.

Supplementary Fig. 17

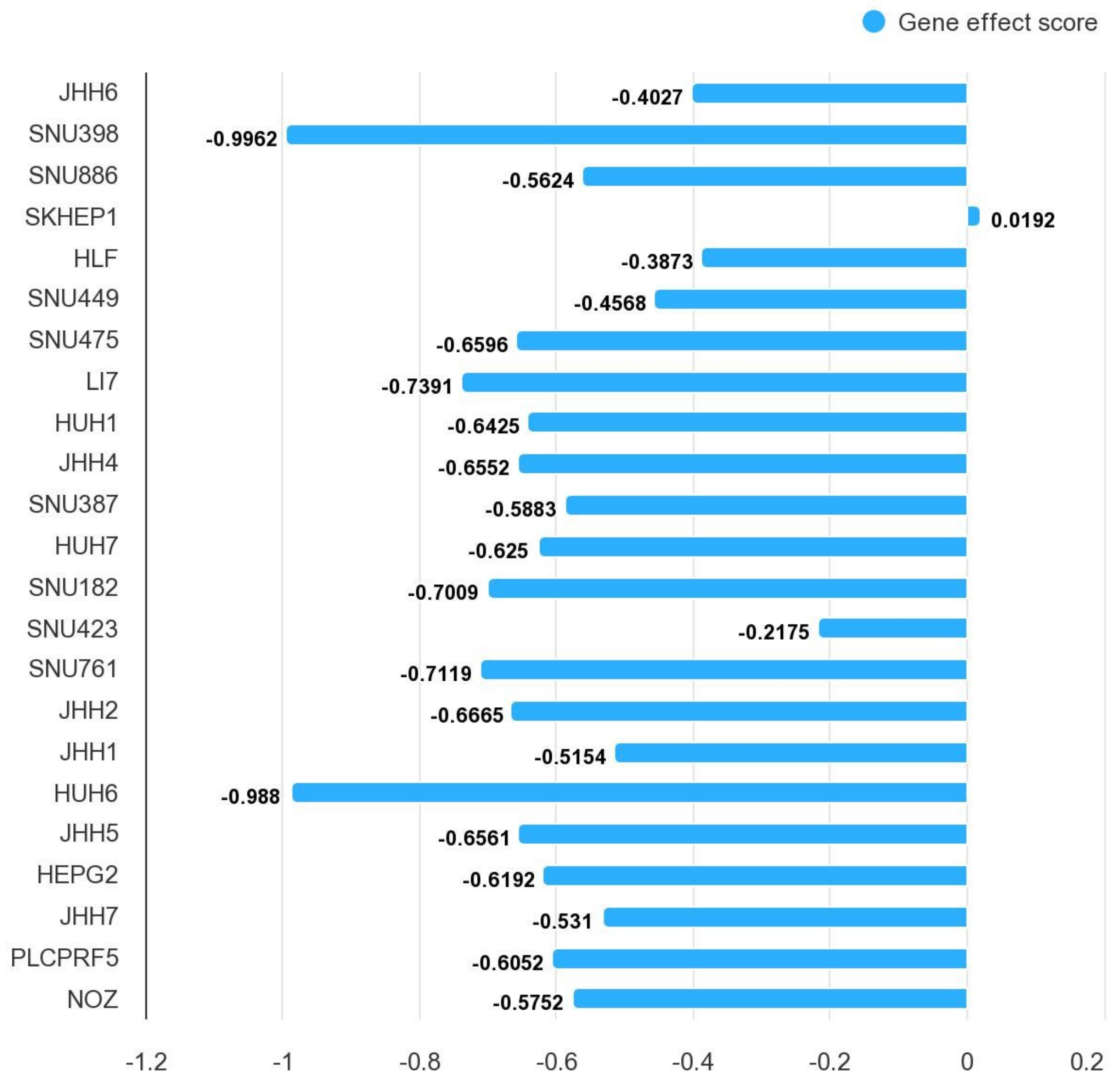

Supplementary Fig. 17. CENPM is critical for HCC cell growth. Depmap results show the dependency of CENPM in a panel of HCC cell lines. Gene Effect scores derived from CRISPR knockout screens published by Broad's Achilles and Sanger's SCORE projects. Negative scores imply cell growth inhibition and/or death following gene knockout. Scores are normalized such that nonessential genes have a median score of 0 and independently identified common essentials have a median score of -1. (Website: https://ualcan.path.uab.edu/cgi-bin/ualcan).

Supplementary Fig. 18

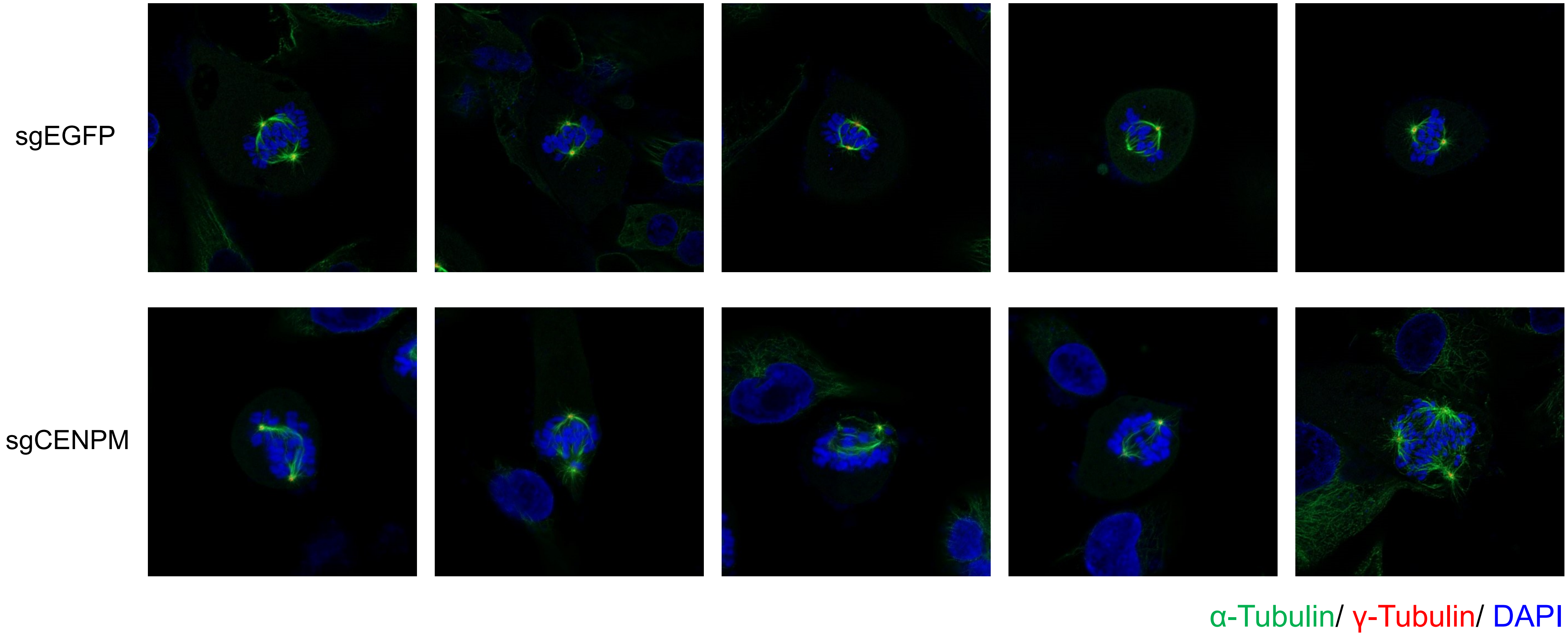

Supplementary Fig. 18. The immunofluorescence-based microscopy analysis of CENPM KO cells. Representative images of immunofluorescence staining of α-tubulin (indicating microtubule, kinetochore or spindle fibers), γ-tubulin (centrosome) and DAPI (indicating chromosomes) in the CENPM KO cells and the control cells during mitosis. Lagging chromosomes or mis-segregation were observed in almost all the CENPM KO cells.

Supplementary Fig. 19

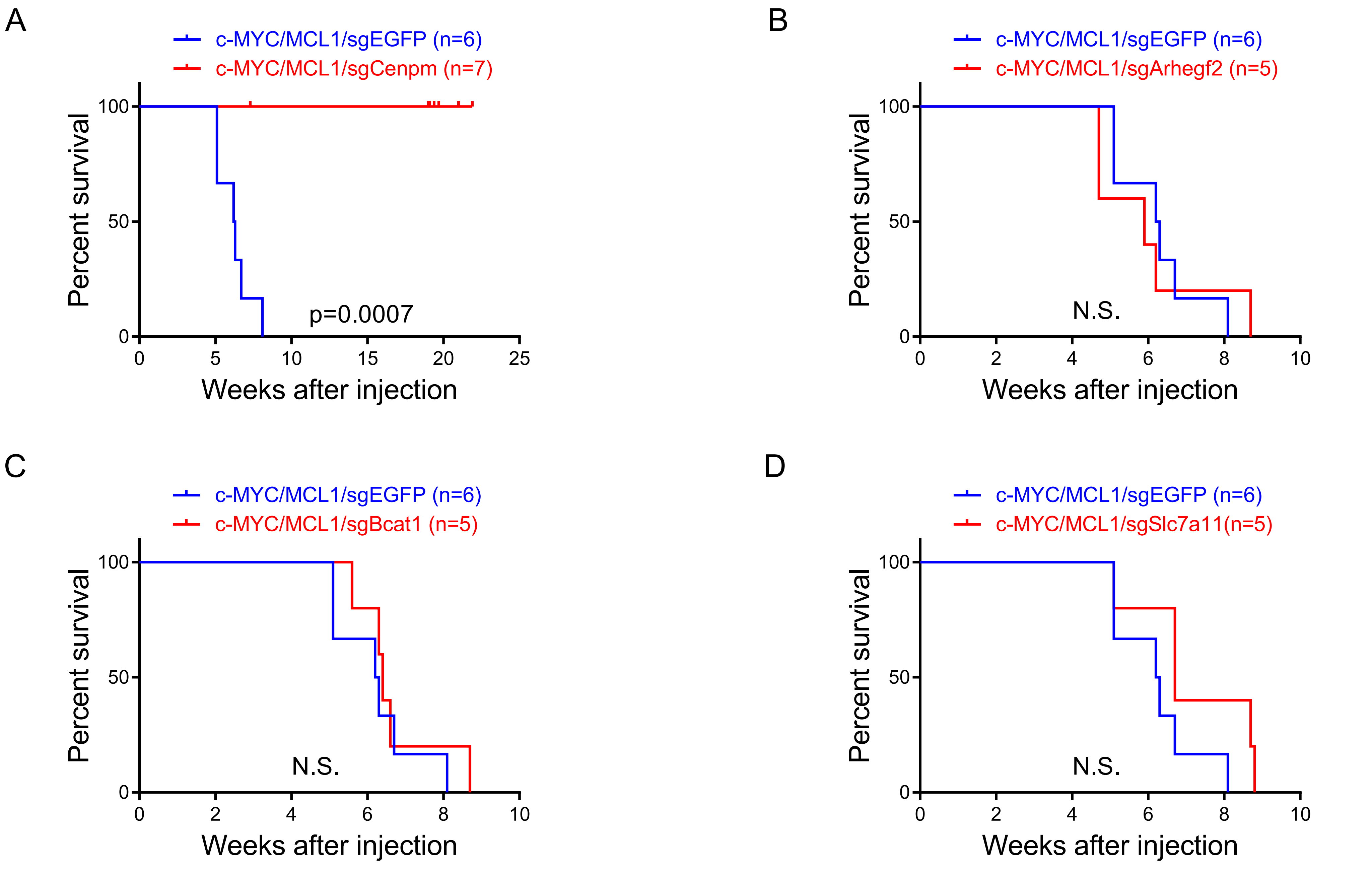

Supplementary Fig. 19. CENPM is a critical target of c-MYC-induced hepatocarcinogenesis. (A-D) Survival curves of mice in the c-MYC/MCL1 HCCs with deletion of *Cenpm* (A), *Arhegf2* (B), *Bcat1* (C), and *Slc7a11* (D). The Kaplan-Meier analysis was performed, and *p*-values are indicated in the graphs.

Supplementary Fig. 20

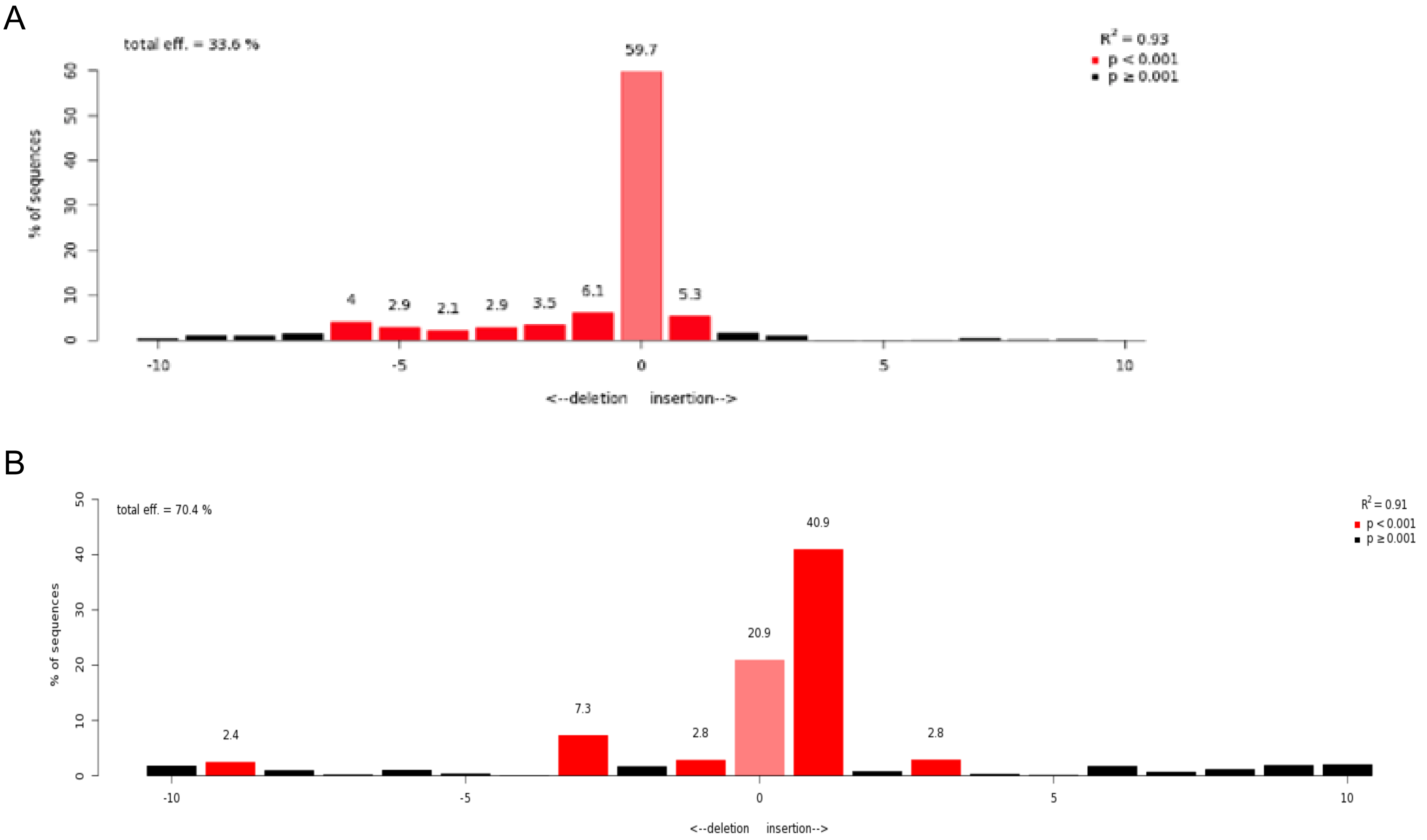

Supplementary Fig. 20. Confirmation of CRISPR-mediated *Cenpm* editing efficiency in the mouse c-MYC HCC cell line (HCC3-4) (A) and the c-MYC mouse model (B) by direct Sanger sequencing.

Supplementary Fig. 21

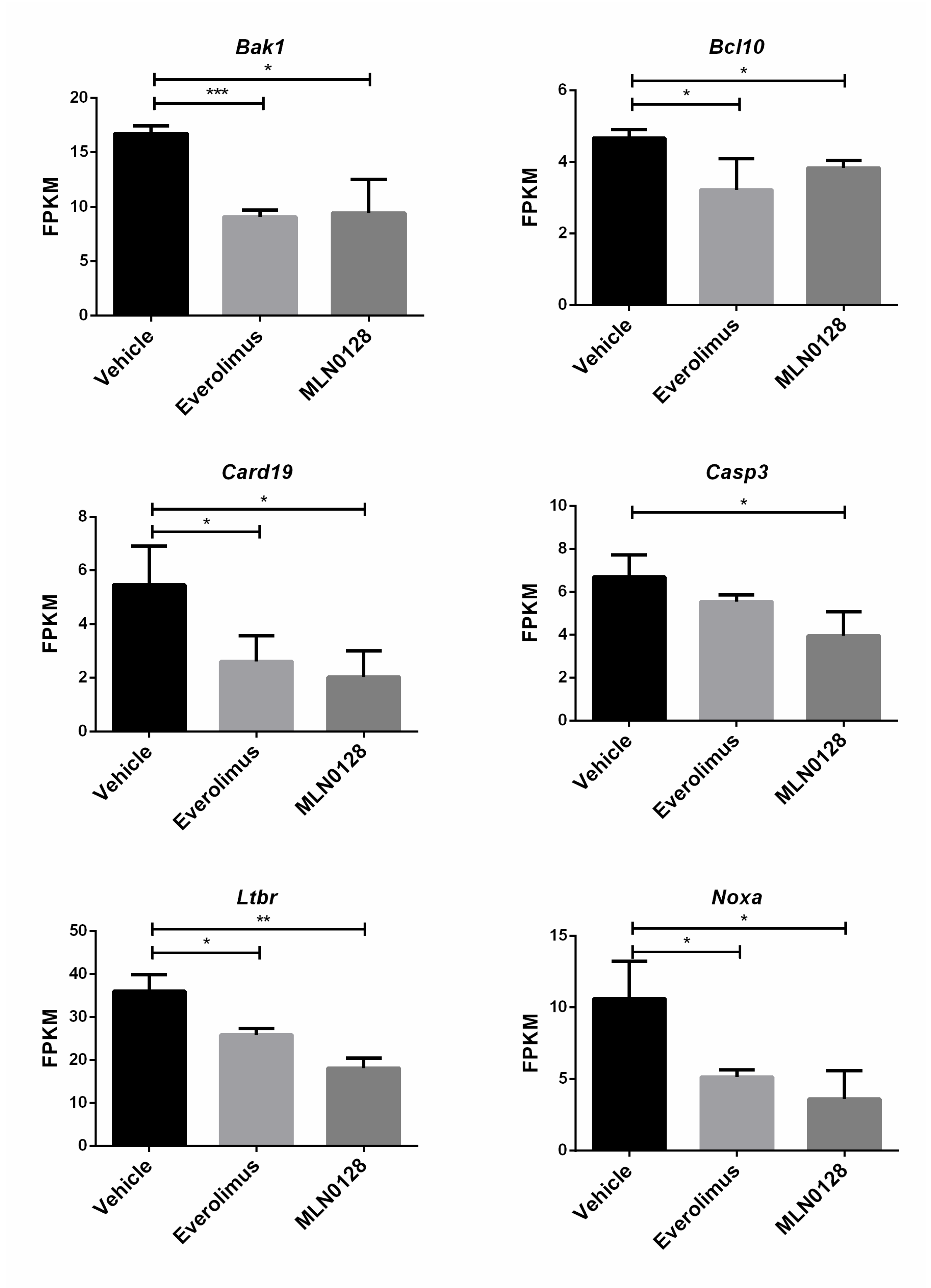

Supplementary Fig. 21. Effects of mTOR inhibitors on apoptosis-related genes in c-MYC/MCL1/*Rictor*^{KO}*Tsc2*^{KO} HCCs. Comparison of the FPKM values for apoptotic genes among vehicle-treated, MLN0128-treated, and Everolimus-treated mouse c-MYC/MCL1/*Rictor*^{KO}*Tsc2*^{KO} HCCs (n = 3, 3, 3). Data are presented as mean ± SD. Tukey–Kramer test. *, $p<0.05$, **, $p<0.01$, ***, $p<0.001$.

Supplementary Fig. 22

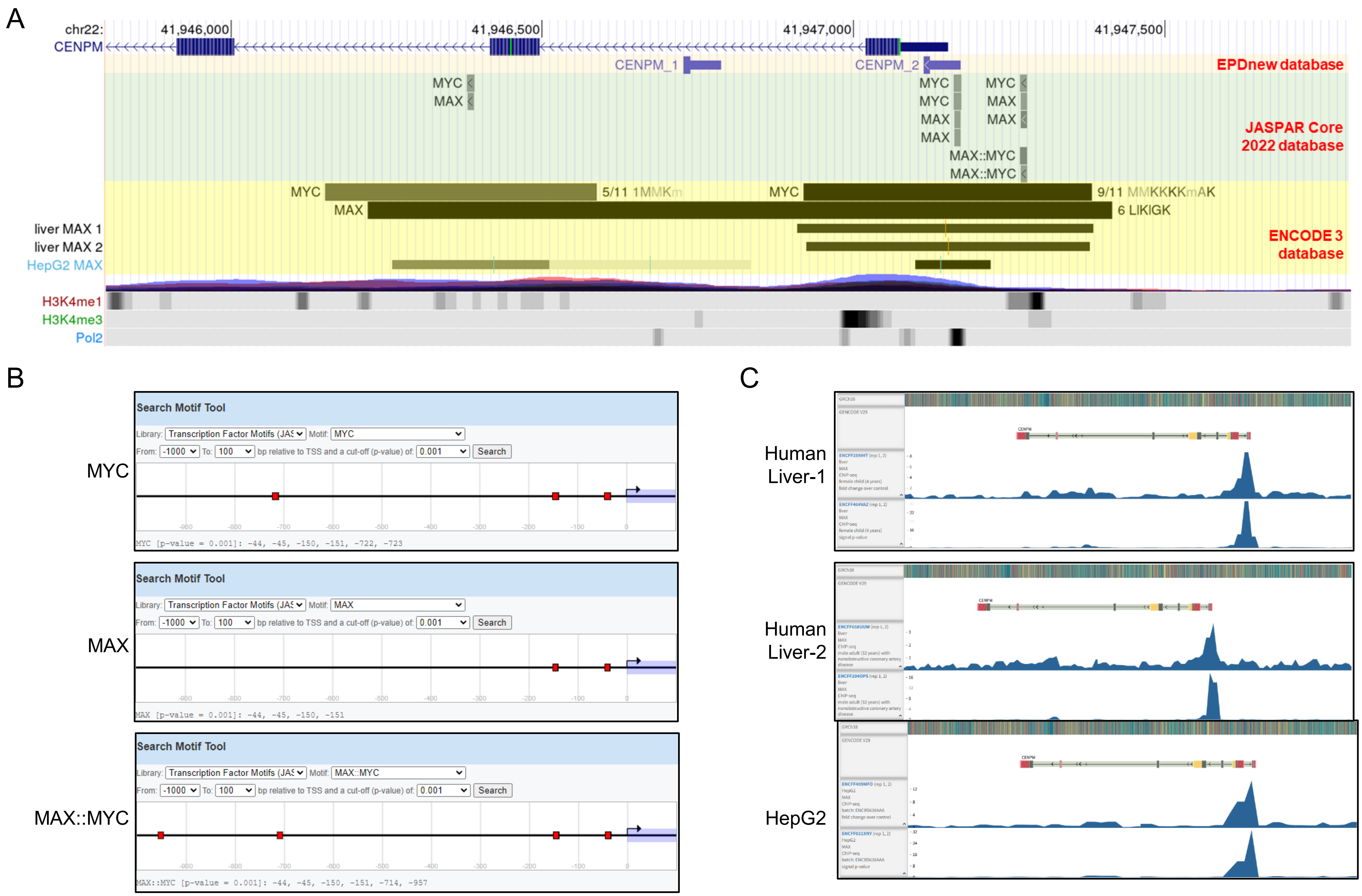

Supplementary Fig. 22. Promoter analysis of CENPM gene. (A) Integration analysis of MYC and MYC::MAX transcription factors with experimentally validated CENPM promoters was performed using various publicly accessible databases. Multiple conserved MYC and MYC::MAX binding sites were identified using the EPDnew database (https://epd.expasy.org/epd/EPDnew_database.php) and the JASPAR core 2022 database (https://jaspar2022.genereg.net/).ChIP-seq data on human liver and HepG2 liver cancer cells from the ENCODE3 database (https://www.encodeproject.org/) also indicated MYC and MAX binding sites on the CENPM promoter region. These sites showed abundant methylation or trimethylation of histone H3 at lysine 4 (H3K4me1 or H3K4me3) and RNA polymerase II (Pol2), which are active marks associated with transcriptional activation. (B) Representative images show the binding sites of MYC, MAX, and the MYC::MAX complex at -1000bp to +100bp relative to the CENPM transcriptional start site, with a cut-off p-value of 0.001. Images were generated from the EPDnew database. (C) Representative images show the bigWig peaks of ChIP-seq analysis for MAX transcription factor using liver tissue from a 4-year-old female child, 32-year-old male adult, and HepG2 cells with CRISPR targeting MYC.

Supplementary Fig. 23

A

Predicted MYC binding site at CENPM promoter region

Score	Relative score	Start	End	Strand	Predicted sequence
12.364184	0.92597599	1874	1885	+	CACCACGTGTTC
12.308522	0.924813923	1980	1991	+	AGGCACGTGCCC
12.217053	0.922904317	1980	1991	-	GGGCACGTGCCT
10.374247	0.88443179	1874	1885	-	GAACACGTGGTG
8.3263645	0.841677879	1302	1313	+	ACCCACCTGGGC
7.2563725	0.81933951	1302	1313	-	GCCCAGGTGGGT

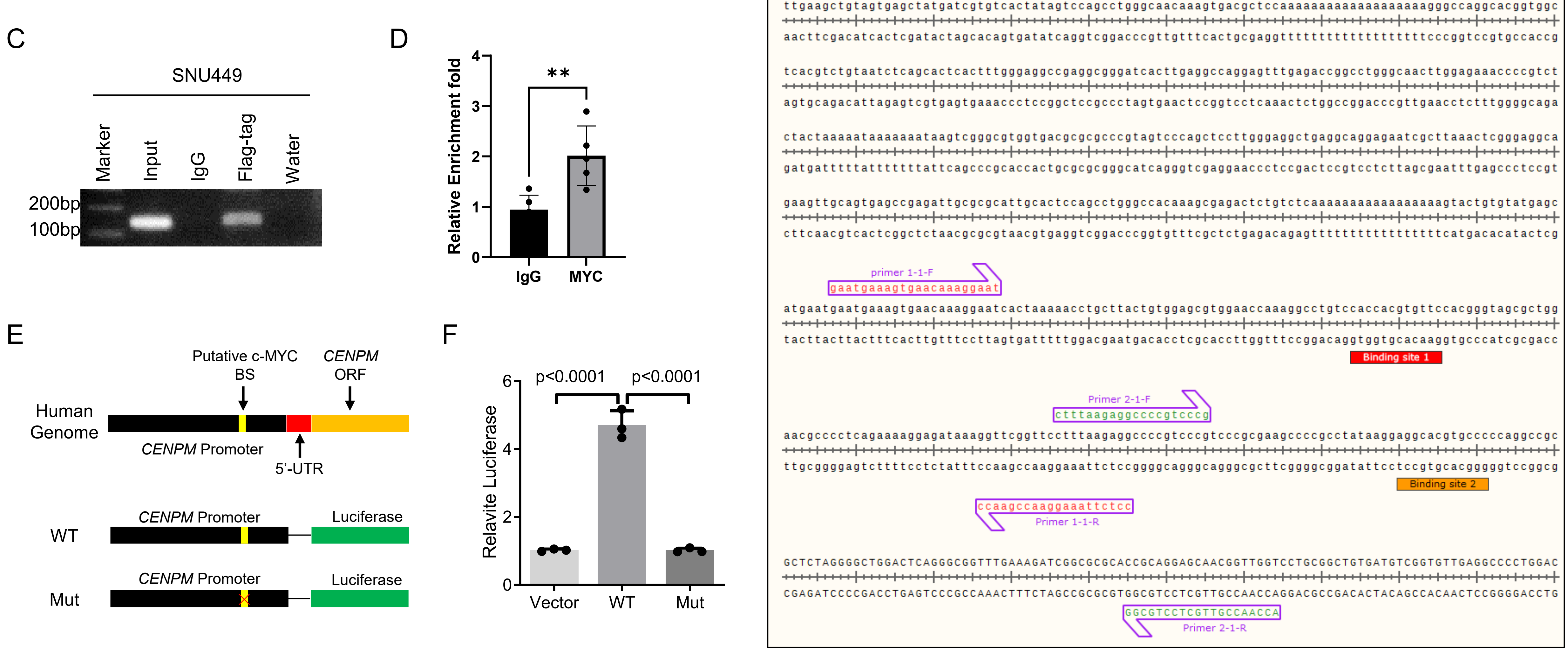

Supplementary Fig. 23. CENPM is a direct transcriptional target of c-MYC. (A) Predicted MYC binding sites at CENPM promoter region. **(B)** Primer design for CHIP and CUT & RUN assay on the CENPM promoter region. **(C)** ChIP assay on the promoter of CENPM. pCMV4a-Flag-c-Myc transfected SNU449 cells were used. **(D)** CUT & RUN assay for CENPM promoter in SNU449 cells. Data are presented as mean ± SD. Student's t-test. ns, no significant. **, *p*<0.01. **(E)** Experimental design of the luciferase reporter assay. **(F)** Luciferase reporter assay for the identification of MYC binding sites in the CENPM gene promoter region (n = 3, 3, 3). Data are presented as mean ± SD. Tukey–Kramer test and *p*-values are indicated in the graphs.

TSC/mTORC1 mediates mTORC2/AKT1 signaling in c-MYC–induced murine hepatocarcinogenesis via centromere protein M

Yi Zhou[1, 2*], Shu Zhang[3,4*], Guoteng Qiu[5,6], Xue Wang[7], Andrew Yonemura[7], Hongwei Xu[2,5], Guofei Cui[2,7], Shanshan Deng[2,7], Joanne Chun[7], Nianyong Chen[3,4,8], Meng Xu[9], Xinhua Song[10], Jingwen Wang[10], Zijing Xu[10], Youping Deng[7,11], Matthias Evert[12], Diego F. Calvisi[12], Shumei Lin[1], Haichuan Wang[5,6] and Xin Chen[2,7]

Supplementary Tables

Supplementary Table 1. LogFPKM data for NL, c-MYC/pT3, and c-MYC/FoxO1AAA mouse models. List of genes that are upregulated in c-MYC/pT3 but downregulated by FOXO1AAA, and list of genes that are downregulated in c-MYC/pT3 but upregulated by FOXO1AAA. Please refer to the excel file for the detailed information.

Supplementary Table 2. List of guide RNA used for CRISPR-Cas9 mediated gene deletion

Guide RNA	Sequence
Human *CENPM*	AACACGATCAGGTCAATTCG
Mouse *Cenpm*	AACACAATCAGGTCAATTCG
Mouse *Arhgef2*	CCACAGACTCCCTCAACATG
Mouse *Bcat1*	TGGTGTGATGATGAGATCTT
Mouse *Slc7a11*	GCACAACTGGCTTTCTGACC

Supplementary Table 3. List of mice used for the experiments

Directory	Mice breed	Plasmid injected (μg)	Sample size (n)
Figure 1	*Rictor*$^{fl/fl}$;*Foxo1*$^{fl/fl}$	c-MYC(10)+MCL1(10)+Cre(20)+SB(1.6)	6
		c-MYC(10)+MCL1(10)+pCMV(20)+SB(1.6)	6
Figure 2	FVB/N	c-MYC(10)+Foxo1AAA(40)+SB(2)	5
		c-MYC(10)+ pT3(40)+SB(2)	5
Figure 3	*Rictor*$^{fl/fl}$;*Tsc2*$^{fl/fl}$	c-MYC(10)+MCL1(10)+Cre(20)+SB(1.6)	6
		c-MYC(10)+MCL1(10)+pCMV(20)+SB(1.6)	4
Figure 4	*Rictor*$^{fl/fl}$;*Tsc2*$^{fl/fl}$	c-MYC(10)+MCL1(10)+Cre(20)+SB(1.6) treated with MLN0128	3
		c-MYC(10)+MCL1(10)+Cre(20)+SB(1.6) treated with vehicle	5
Figure 7	FVB/N	c-MYC(10)+MCL1(10)+SB(0.8)+sgCENPM(40)	7
		c-MYC(10)+MCL1(10)+SB(0.8)+sgEGFP(40)	6
Sup Fig.3	FVB/N	c-MYC(10)+Foxo3AAA(40)+SB(2)	3
		c-MYC(10)+ pT3(40)+SB(2)	3
Sup Fig.7	*Tsc2*$^{fl/fl}$	c-MYC(10)+Cre(20)+SB(1.2)	4
		c-MYC(10)+pCMV(20)+SB(1.2)	6
Sup Fig.4	*Raptor*$^{fl/fl}$	c-MYC(10)+MCL1(10)+TTR-CreERT2(20)+SB(1.6) treated with Tamoxifen	7
		c-MYC(10)+MCL1(10)+TTR-CreERT2(20)+SB(1.6) treated with vehicle	5
		c-MYC(10)+MCL1(10)+TTR-CreERT2(20)+SB(1.6) pretreatment	3
Sup Fig.7	*Rictor*$^{fl/fl}$;*Tsc2*$^{fl/fl}$	c-MYC(10)+MCL1(10)+Cre(20)+SB(1.6) treated with Everolimus	5
		c-MYC(10)+MCL1(10)+Cre(20)+SB(1.6) treated with vehicle	5
Sup Fig.8	*Rictor*$^{fl/fl}$;*Tsc2*$^{fl/fl}$	c-MYC(10)+MCL1(10)+Cre(20)+SB(3.2)+4EBP1A4(40)	7
		c-MYC(10)+MCL1(10)+Cre(20)+SB(3.2)+pT3(40)	6

Sup Fig.12	FVB/N	c-MYC(10)+MCL1(10)+SB(0.8)+sgEGFP(40)	6
		c-MYC(10)+MCL1(10)+SB(0.8)+sgArhegf2(40)	5
		c-MYC(10)+MCL1(10)+SB(0.8)+sgBcat1(40)	5
		c-MYC(10)+MCL1(10)+SB(0.8)+sgSLC7a11(40)	5

Supplementary Table 4. List of antibodies used for immunohistochemistry

Antibody	Catalog Number	Company	Species	Dilution
c-MYC	ab32072	Abcam	Rabbit	1:200
Myc-tag	Vli01	MMCRI	Rabbit	1:200
Ki-67	12202	Cell Signaling Technology	Rabbit	1:150
Cleaved Caspase-3	9664	Cell Signaling Technology	Rabbit	1:150
Raptor	MA5-35742	Thermo Fisher Scientific	Rabbit	1:100
FOXO1	2880	Cell Signaling Technology	Rabbit	1:100
FOXO3	12829	Cell Signaling Technology	Rabbit	1:100
phospho-FOXO1 (Ser 256)	PA5-104977	Thermo Fisher Scientific	Rabbit	1:100
phospho-FOXO3 (Ser 253)	PA5-118528	Thermo Fisher Scientific	Rabbit	1:100

Supplementary Table 5. List of antibodies used for Western blot analysis

Antibody	Catalog Number	Company	Species	Dilution
AKT	9272	Cell Signaling Technology	Rabbit	1:1000
Phospho-AKT (Ser473)	4060	Cell Signaling Technology	Rabbit	1:1000
Rictor	9476	Cell Signaling Technology	Rabbit	1:1000
FOXO1	2880	Cell Signaling Technology	Rabbit	1:1000
Phospho-FOXO1 (Ser256)	84192	Cell Signaling Technology	Rabbit	1:1000
S6 Ribosomal Protein	2217	Cell Signaling Technology	Rabbit	1:1000
Phospho-S6 Ribosomal Protein (Ser235/236)	4858	Cell Signaling Technology	Rabbit	1:1000
4E-BP1	9644	Cell Signaling Technology	Rabbit	1:1000
Phospho-4E-BP1 (Ser65)	9451	Cell Signaling Technology	Rabbit	1:1000
c-MYC	ab32072	Abcam	Rabbit	1:2000
Cleaved Caspase-3	9664	Cell Signaling Technology	Rabbit	1:1000
Cyclin D1	2978	Cell Signaling Technology	Rabbit	1:1000
LDHA/C	3558	Cell Signaling Technology	Rabbit	1:1000
PKM1	7067	Cell Signaling Technology	Rabbit	1:1000
CENPM	ab243820	Abcam	Rabbit	1:1000
GAPDH	5174	Cell Signaling Technology	Rabbit	1:10000

β-actin	4970	Cell Signaling Technology	Rabbit	1:1000

Supplementary Table 6. List of primers used in qRT-PCR analysis

Gene name	Forward	Reverse
18s rRNA	CGGCTACCACATCCAAGGAA	GCTGGAATTACCGCGGCT
Human-CENPM	GCGGACTCGATGCTCAAAGA	TTCTGGAGACTGTATTTGCTGTG
