# Supplementary material for: TSC/mTORC1 mediates mTORC2/AKT1 signaling in c-MYC–induced murine hepatocarcinogenesis via centromere protein M
Source: J Clin Invest. 2024 Sep 26;134(22):e174415. doi: 10.1172/JCI174415 (PMC11563669; doi:10.1172/JCI174415)

Fig. 3D

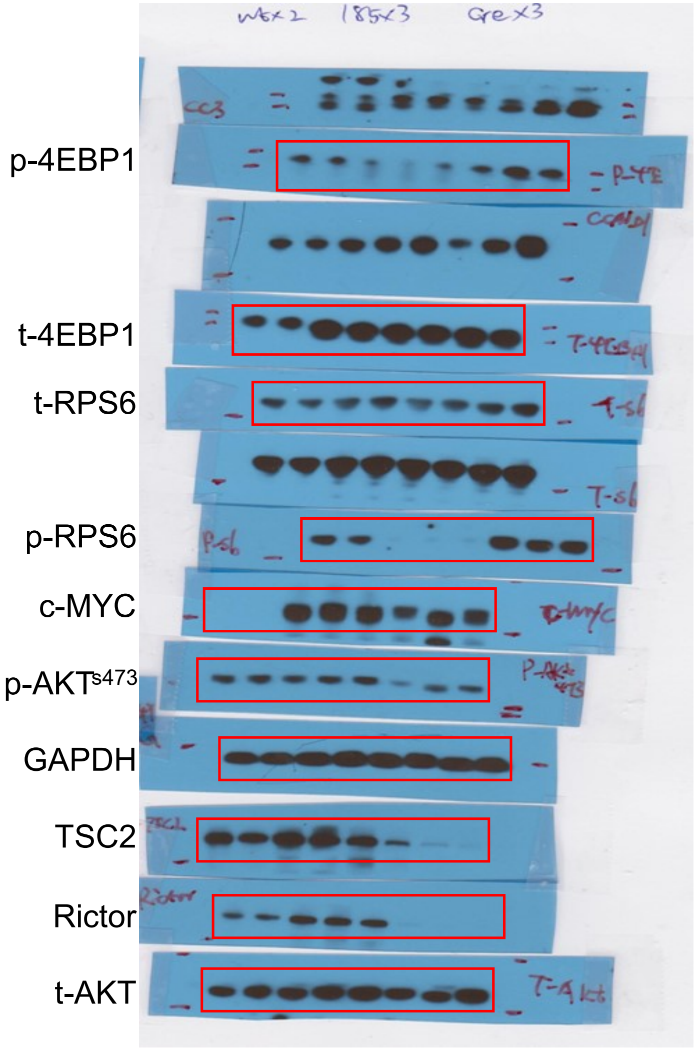
p-4EBP1
t-4EBP1
t-RPS6
p-RPS6
c-MYC
p-AKTs473
GAPDH
TSC2
Rictor
t-AKT

Fig. 4C

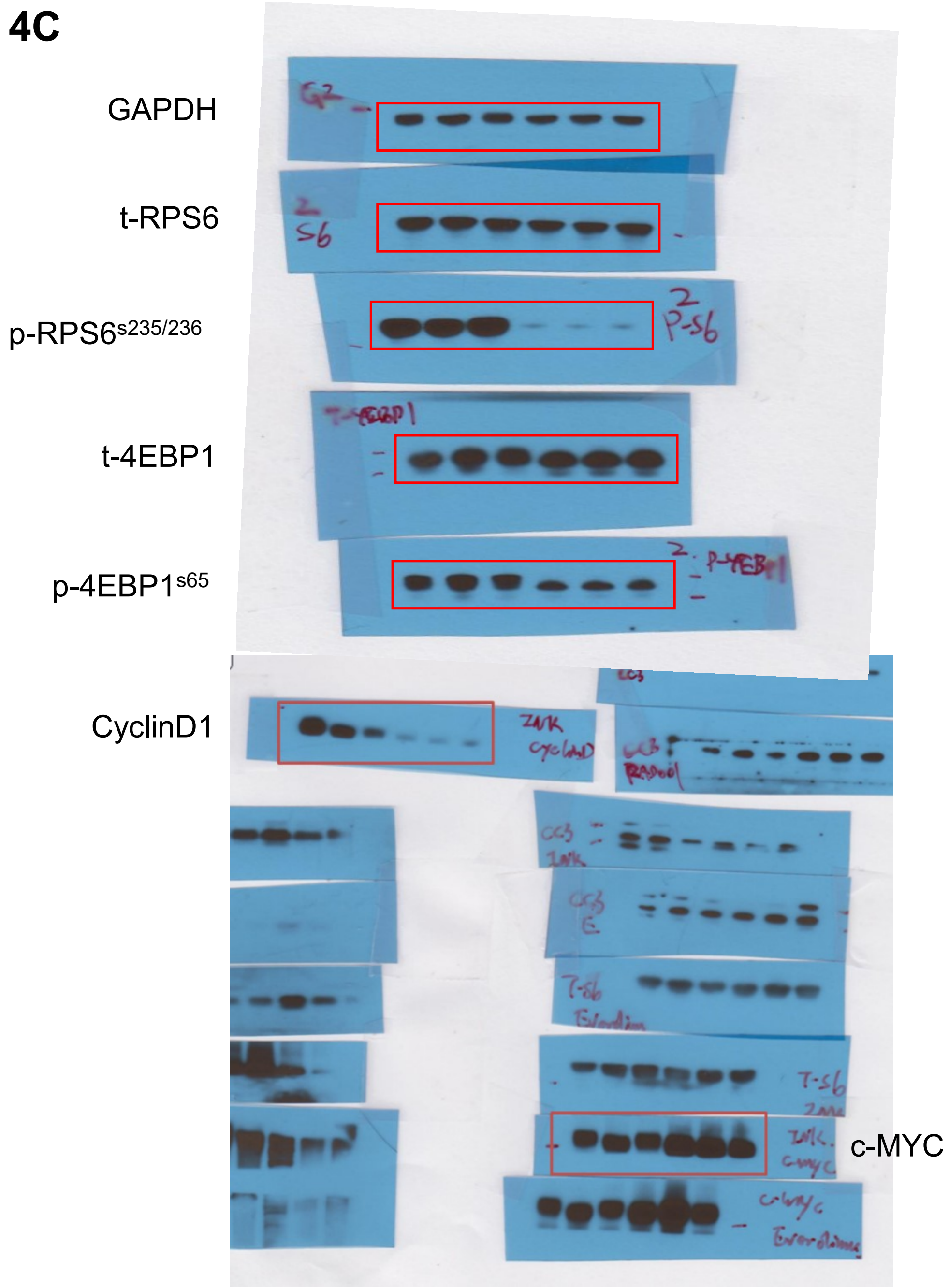

Fig. 6C

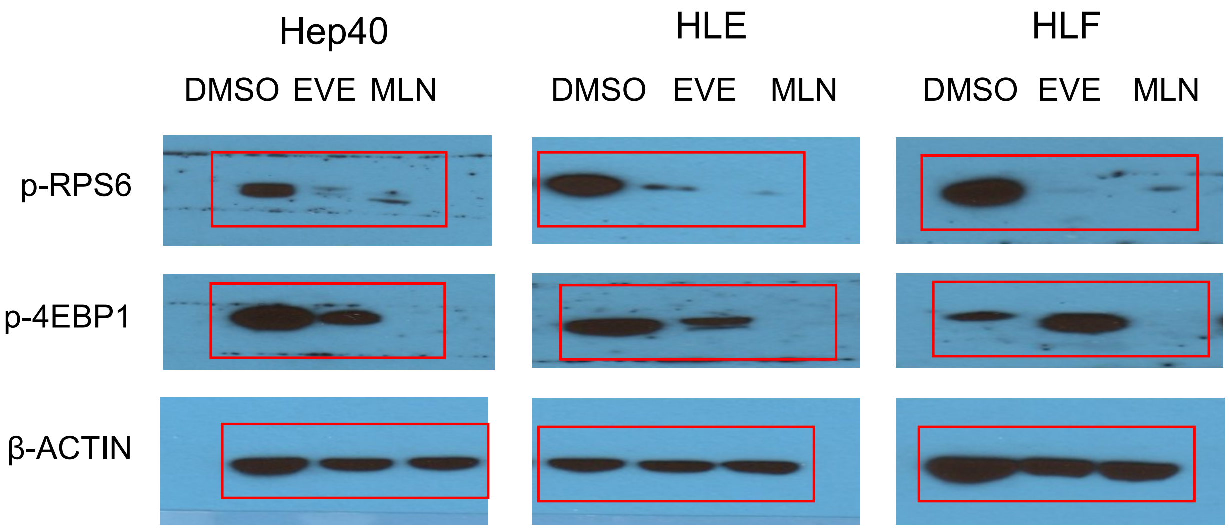
Hep40
HLE
HLF
DMSO EVE MLN
DMSO EVE MLN
DMSO EVE MLN
p-RPS6
p-4EBP1
β-ACTIN

Fig. 7C

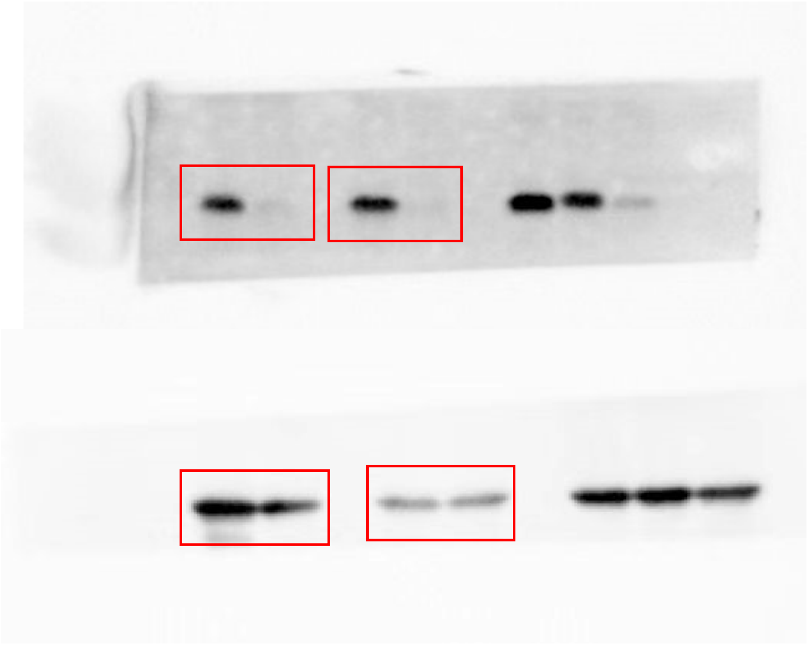

Supplementary Fig. 1B

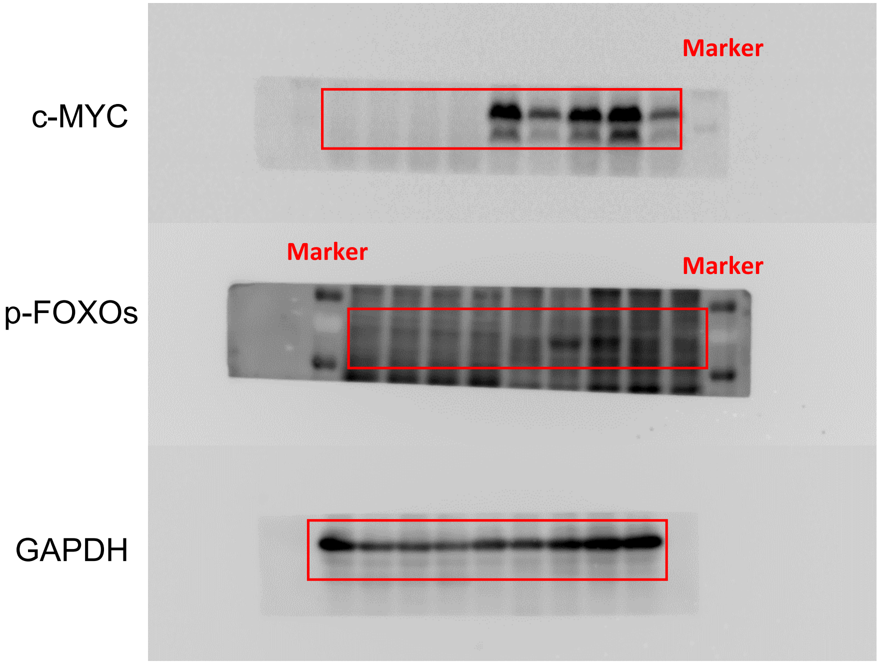

Supplementary Fig. 2A

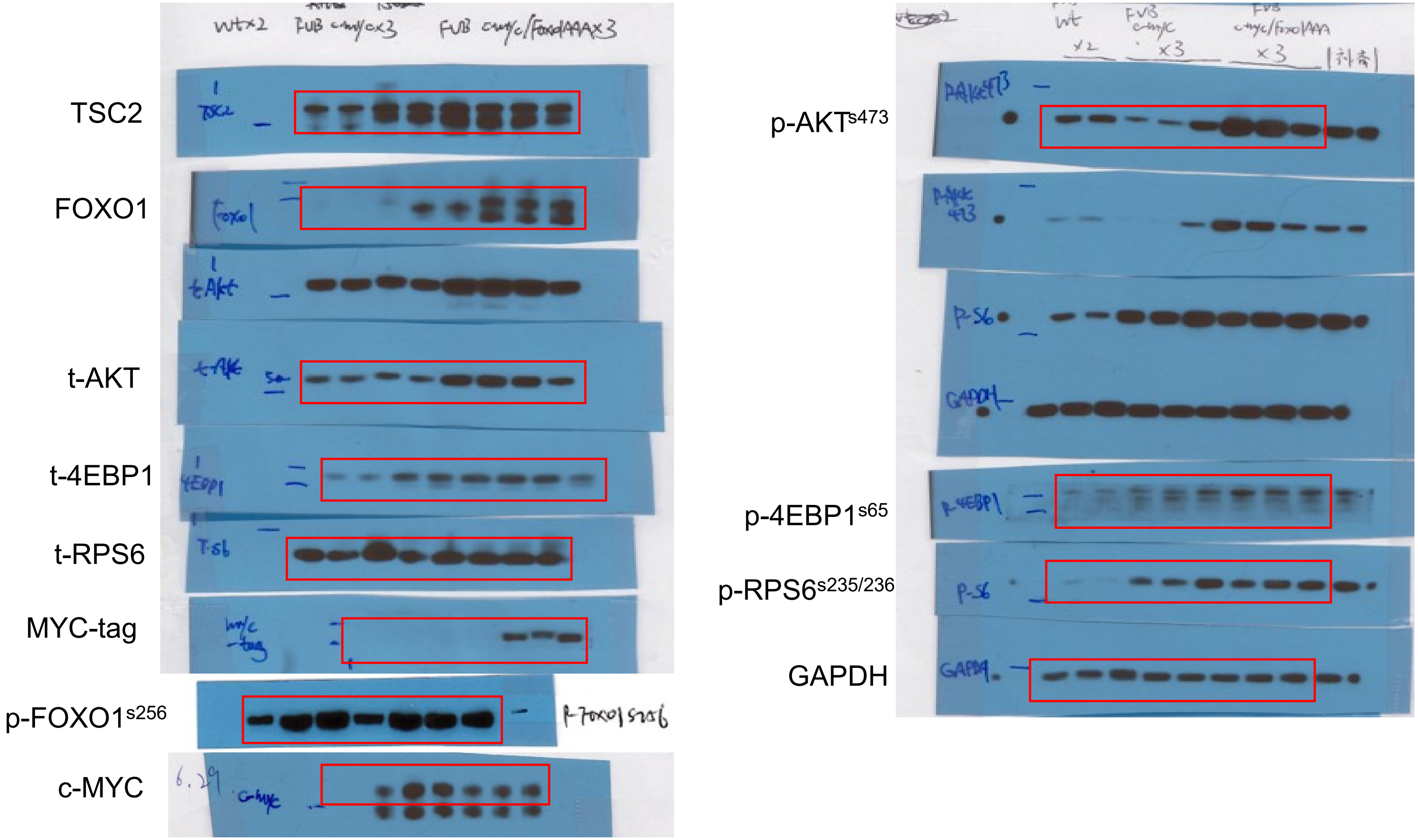

Supplementary Fig. 7D

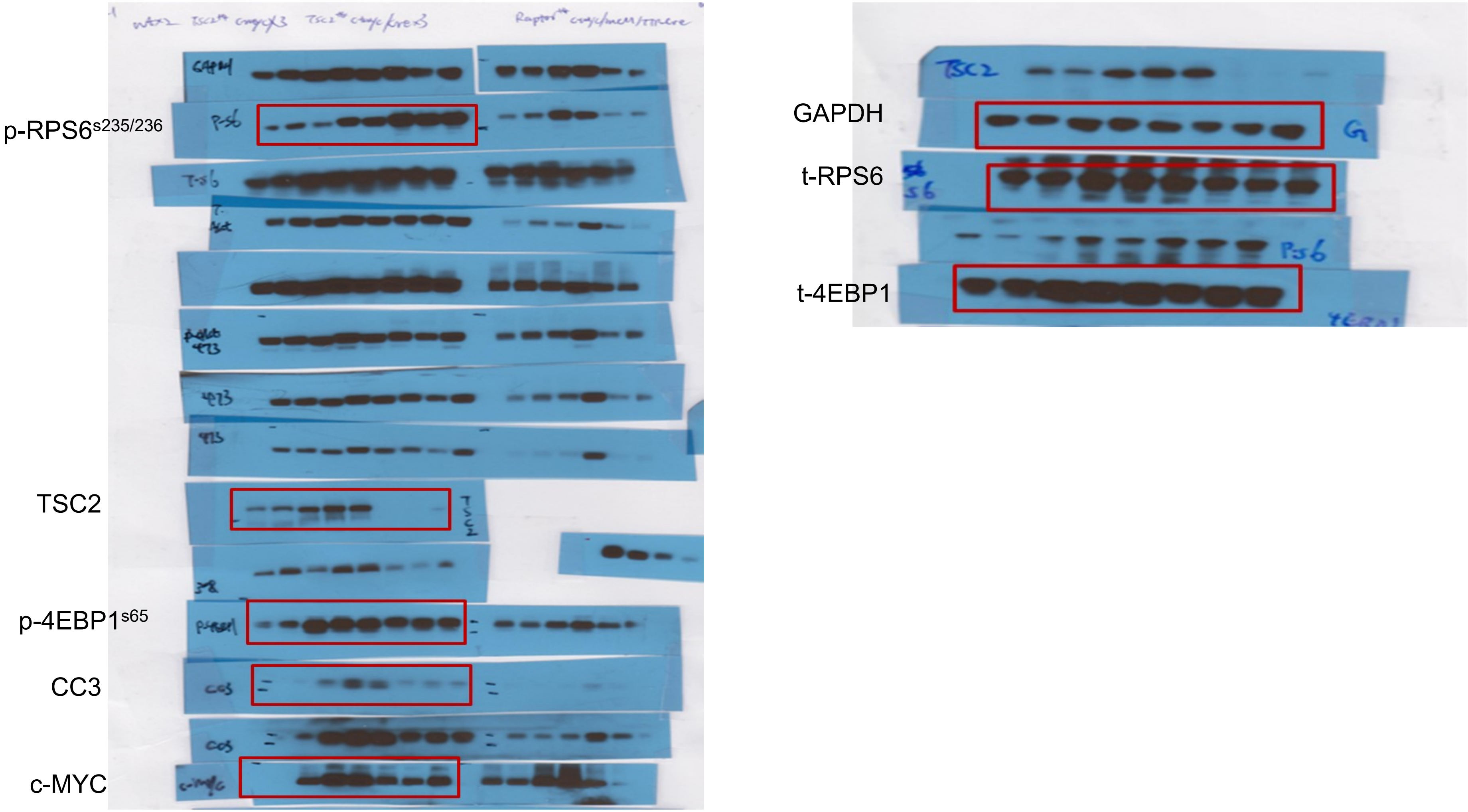

Supplementary Fig. 12C

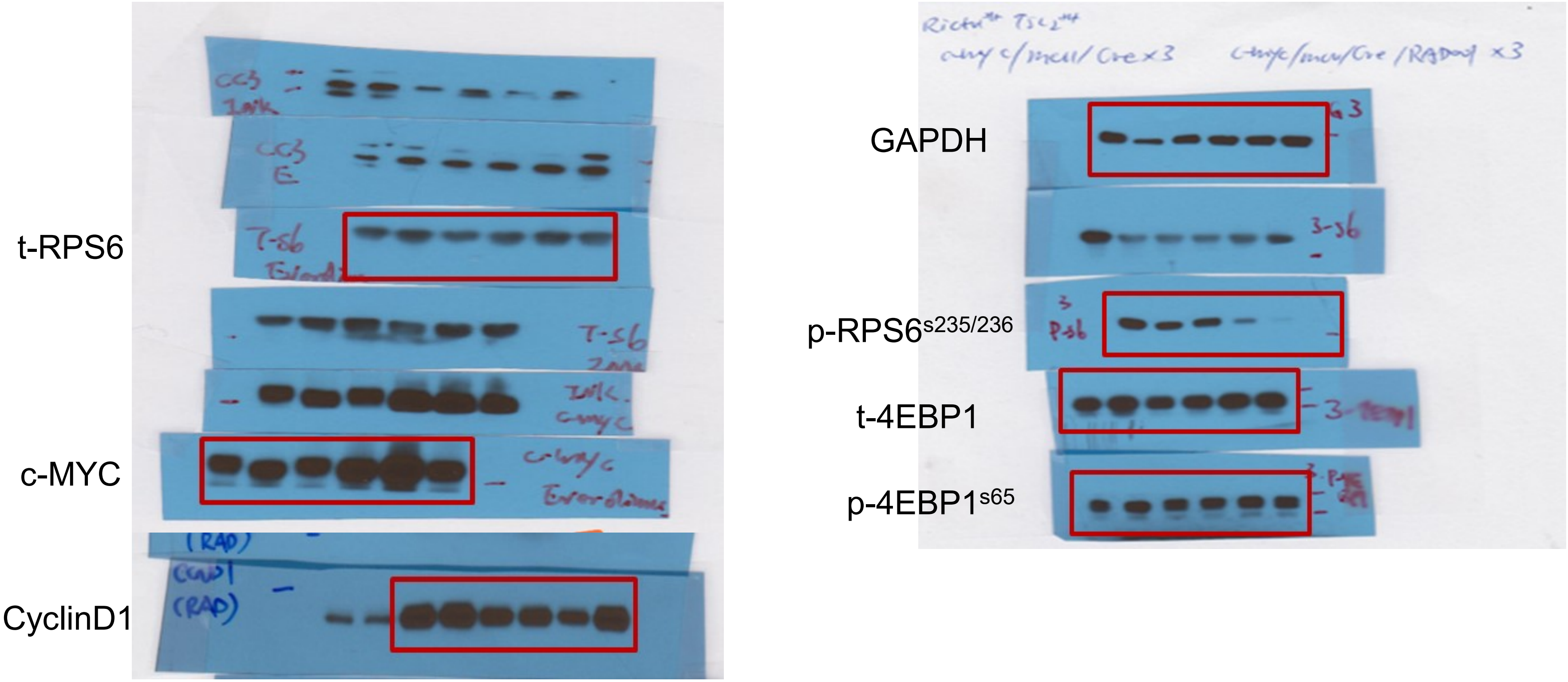

Supplementary Fig. 13D

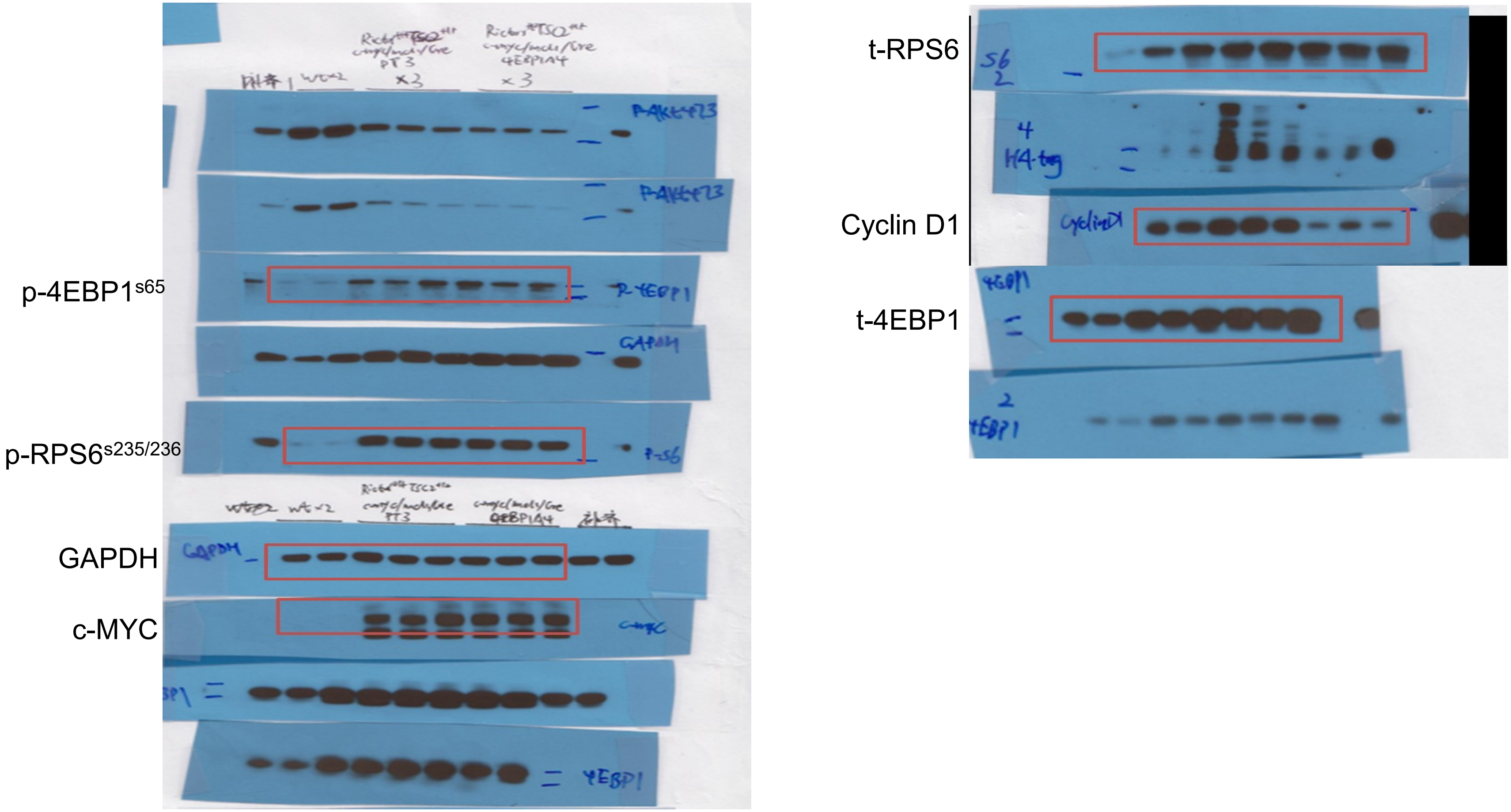

Supplementary Fig. 14B

LDHA/C

PKM1

GAPDH

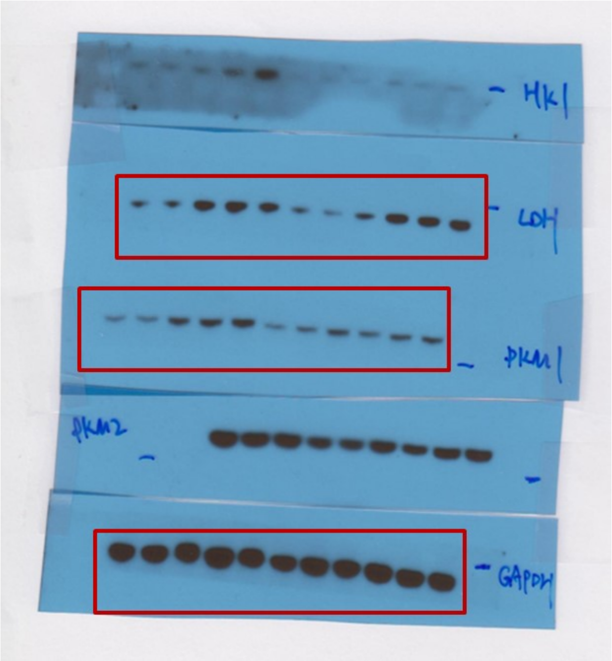

Supplementary Fig. 23C

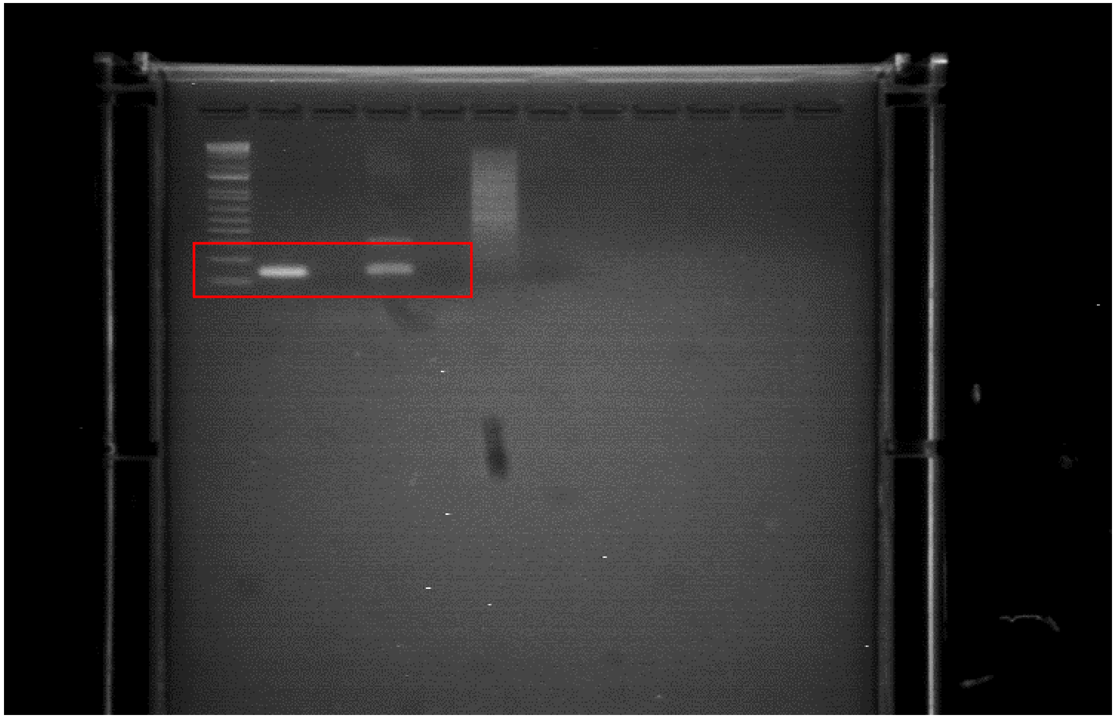

Supplement: Unedited blot and gel images [file jci-134-174415-s206.pdf]
